# Novel Tetrazole Derivatives Targeting Tubulin Endowed with Antiproliferative Activity against Glioblastoma Cells

**DOI:** 10.3390/ijms241311093

**Published:** 2023-07-04

**Authors:** Laura Gallego-Yerga, Andrea Jazmín Chiliquinga, Rafael Peláez

**Affiliations:** 1 Laboratorio de Química Orgánica y Farmacéutica, Departamento de Ciencias Farmacéuticas, Facultad de Farmacia, Universidad de Salamanca, Campus Miguel de Unamuno, 37007 Salamanca, Spain; 2 Instituto de Investigación Biomédica de Salamanca (IBSAL), Facultad de Farmacia, Universidad de Salamanca, Campus Miguel de Unamuno, 37007 Salamanca, Spain; 3 Centro de Investigación de Enfermedades Tropicales de la Universidad de Salamanca (CIETUS), Facultad de Farmacia, Universidad de Salamanca, Campus Miguel de Unamuno, 37007 Salamanca, Spain; 4 Grupo de Investigación de Ciencias en Red, Universidad Técnica del Norte, Ibarra 100105, Ecuador; ajchiliquinga1@utn.edu.ec

**Keywords:** tubulin, glioblastoma, antimitotic drugs, anticancer, colchicine site, tetrazole, indole

## Abstract

Increasing awareness of the structure of microtubules has made tubulin a relevant target for the research of novel chemotherapies. Furthermore, the particularly high sensitivity of glioblastoma multiforme (GBM) cells to microtubule disruption could open new doors in the search for new anti-GBM treatments. However, the difficulties in developing potent anti-tubulin drugs endowed with improved pharmacokinetic properties necessitates the expansion of medicinal chemistry campaigns. The application of an ensemble pharmacophore screening methodology helped to optimize this process, leading to the development of a new tetrazole-based tubulin inhibitor. Considering this scaffold, we have synthesized a new family of tetrazole derivatives that achieved remarkable antimitotic effects against a broad panel of cancer cells, especially against GBM cells, showing high selectivity in comparison with non-tumor cells. The compounds also exerted high aqueous solubility and were demonstrated to not be substrates of efflux pumps, thus overcoming the main limitations that are usually associated with tubulin binding agents. Tubulin polymerization assays, immunofluorescence experiments, and flow cytometry studies demonstrated that the compounds target tubulin and arrest cells at the G2/M phase followed by induction of apoptosis. The docking experiments agreed with the proposed interactions at the colchicine site and explained the structure–activity relationships.

## 1. Introduction

Targeting tubulin is a well-validated strategy for the development of anticancer drugs [1], as demonstrated by the success of the chemotherapy based on taxanes [2] and vinca alkaloids [3]. In this regard, recent studies have shown that glioblastoma multiforme (GBM) cells are particularly sensitive to mitotic destruction compared to healthy cells [4], so the use of therapies that disrupt the microtubule assembly could represent a promising strategy to treat GBM cells, which are the most aggressive and frequent of primary brain tumors [5,6]. Current therapy for GBM is based on the surgical resection of a tumor followed by radiotherapy with concomitant administration of temozolomide (TMZ), a DNA-targeting drug acting via the methylation of guanine bases [7,8]. The difficulties associated with the removal of tumors together with the low efficiency of TMZ and the acquired resistance to alkylating agents, which is usually exhibited by malignant gliomas [9,10], involve a serious impediment in the treatment of these tumors, resulting in a poor prognosis and high mortality rate. Despite efforts being made to develop new therapies for this kind of brain cancer [11,12,13,14,15], none of them have led to significant improvements in patient survival; therefore, the search for new targets, like tubulin, could open new roads in the treatment of GBM.

Tubulin is the main constituent of microtubules, which are part of the cytoske-leton of eukaryotic cells and have an essential role in cell division. The polymerization–depolymerization equilibria of microtubules are the basis of the function of the mitotic spindle, acting during cell mitosis for the accurate segregation of chromosomes between the two daughter cells [16]. Tubulin ligands exert their anticancer effect through the promotion or the inhibition of tubulin polymerization [17,18,19], leading to mitotic arrest and disruption in both cases, which is usually followed by the induction of apoptosis [20]. 

Although several binding sites have been described in tubulin [21,22], only the taxanes and the vinca alkaloids have representatives in clinical use [23,24]; however, they show poor pharmacokinetic properties and have a drug resistance that is associated with multidrug resistance proteins (MDRs) [25,26]. The colchicine binding site in tubulin, which is composed of three interconnected sub-pockets (A, B, and C) [27], represents a good alternative to taxanes and vinca alkaloids since its ligands are small and synthetically accessible molecules, which are not primed to develop MDR. A further advantage is that colchicine ligands can act not only as tubulin polymerization inhibitors but also as vascular disrupting agents [28,29].

**Figure 1:**
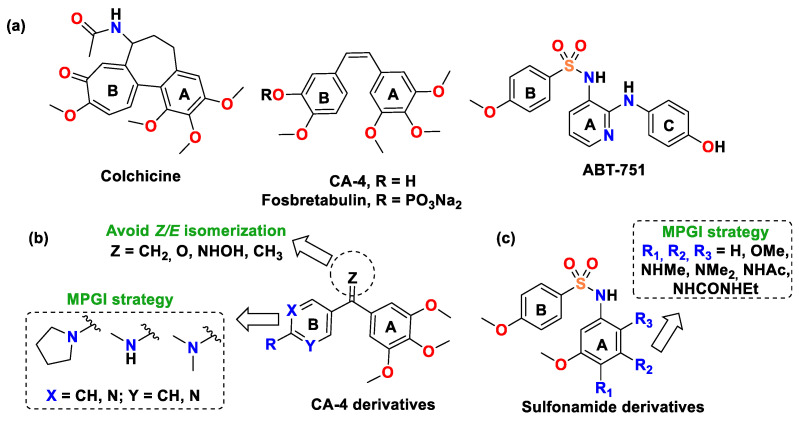
(**a**) Chemical structures of some representative colchicine-site ligands. Rings A, B, and C are indicated; (**b**) chemical modifications of CA-4 derivatives to avoid *Z/E* isomerization and improve aqueous solubility through the masked polar group incorporation (MPGI) strategy applied to ring B; (**c**) MPGI strategy applied to ring A of sulfonamide derivatives.

However, the most promising representatives of colchicine site ligands have not succeeded and have failed to reach clinical applications mainly due to high toxicity (e.g., colchicine) [30], low potency (e.g., ABT-751) [31], or low water solubility, as is the case with combretastatin A4 (CA-4) [32] (Figure 1a). CA-4 also suffers from isomerization from the active *Z* isomer to the less potent *E* isomer. 

The high potency of CA-4 has encouraged researchers to search for non-isomerizable and aqueous soluble analogs of CA-4 [33]. The solubility limitations of CA-4 have been overcome by the phosphate pro-drug fosbretabulin [34] (Figure 1a) and by the substitution of the methoxyphenol B ring of CA-4 by other functionalities, such as a pyridine moiety containing masked polar groups (masked polar group incorporation, MPGI, Figure 1b) [35]. This strategy has also been applied to the A rings of sulfonamide derivatives (Figure 1c) [36]. On the other hand, the configurational stability of CA-4 derivatives has been achieved through the substitution of the olefinic bridge by one atom linker [37,38], as is the case in isocombretastatins [39,40] and phenstatins (Figure 1b) [41].

**Figure 2:**
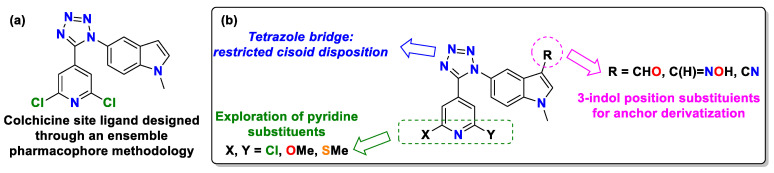
(**a**) Colchicine site ligand reported in previous work; (**b**) design of a novel family of 5-pyridyl-1-tetrazol-1-yl-indole derivatives.

Unfortunately, besides the increasing efforts to develop antimitotics targeting the colchicine site, small percentages of new colchicine ligands with improved pharmacokinetic properties have been shown to maintain high antimitotic potency in the low nanomolar range exerted by CA-4 [42], thus extending the medicinal chemistry campaigns with too many cycles of drug design, synthesis, and biological evaluation [43]. To shorten and optimize the design process, in a previous work, we developed a computational screening methodology that succeeded in designing a new active molecule targeting the colchicine site. The method was based on an ensemble pharmacophore generated from more than eighty published X-ray structures of tubulin in complex with colchicine site ligands. The ensemble pharmacophore was used to search the ZINC database, where more than 8000 structures were selected and evaluated by two different pharmacophore-matching programs. After examining the information we obtained via virtual screening, we identified some structural elements for the design of a new compound family, namely, indole, tetrazole, and halogenated pyridine, and we proposed the synthesis and evaluation of 5-(5-(2,6-dichloropyridin-4-yl)-1*H*-tetrazol-1-yl)-1-methyl-1*H*-indole (Figure 2a). The synthesized compound exhibited high potency as a tubulin polymerization inhibitor and antiproliferative activity in the nanomolar range against human epithelioid carcinoma HeLa cells (IC_50_ of 45 nM), showing the ability to cause mitotic arrest and microtubule network disruption [44]. The substitution pattern of the tetrazole scaffold allowed the compound to adopt a conformation adapted to the A-B subpockets of the colchicine binding site. The aromatic rings lacked hydroxyl groups, which are prompted to be subjected to metabolization, and the endocyclic heteroatoms helped improve the water solubility of the compound. According to docking studies, the compound demonstrated the ability to establish favorable interactions with the aminoacid residues of the colchicine site through the chlorine and nitrogen atoms, and the size of the aromatic rings was suitable, facilitating interactions in the A-B zone. 

The success of the proof of concept study opened new doors for us to further explore the potential of this family of molecules. Considering the skeleton of the reported lead compound (Figure 2a), we synthesized a novel series of 5-pyridyl-tetrazol-1-yl-indole derivatives by introducing different substituents at the indole and pyridine moieties in the hopes of increasing the antiproliferative activity of compounds against cancer cells, as well as aqueous solubility (Figure 2b). We considered leaving the tetrazole scaffold unaltered since it ensures a cisoid conformation of the aromatic rings, which is necessary for interactions at the binding site. Furthermore, the tetrazole moiety has emerged as a promising facilitator of antimitotic colchicine site ligand development [45,46,47,48,49,50]. On the other hand, the indole scaffold has been demonstrated to endow different kinds of compounds with antiproliferative activity [51,52] and could serve as an anchor for further derivation aimed at the development of pro-drugs [53], whereas the replacement of chlorine atoms in the pyridine ring by methoxy or methylsulfanyl groups could modulate the interaction with tubulin as well as the activity on different cancer cell lines, as reported in previous works [54,55]. 

The newly synthesized compounds exhibited high aqueous solubility, which is an important issue regarding colchicine site ligands, and exerted antiproliferative activities in a panel of different cell lines that are representative of some of the kinds of cancer that have the highest incidence worldwide, including the following [56]: cervical, breast, liver, colon, and glioblastoma. The compounds were particularly potent against GBM cell lines and displayed low toxicity in the non-tumor cell line HEK-293, thus demonstrating their high selectivity toward cancer cells. Their mechanism of action was studied through tubulin polymerization inhibition assays, immunofluorescence experiments conducted via the use of confocal microscopy, cell cycle studies, and characterization of cell death by flow cytometry. Computational studies were performed to propose the binding modes of the compounds at the colchicine site of tubulin and explain the observed structure–activity relationships.

## 2. Results and Discussion 

### 2.1. Chemical Synthesis

**Scheme 1:**
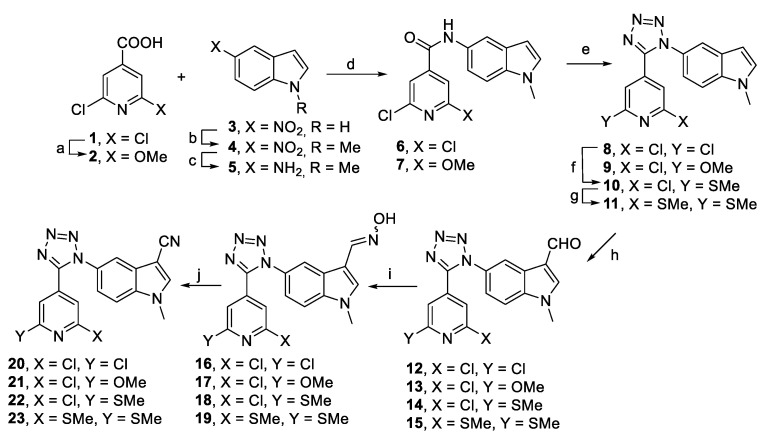
Synthesis of tetrazole derivatives. Reagents and conditions: (a) NaOMe, MeOH, rt, 24 h; (b) MeI, NaOH, n-Bu_4_NHSO_4_, CH_2_Cl_2_, rt, 24 h; (c) H_2_, Pd/C, MeOH, CH_2_Cl_2_, rt, 24 h; (d) EDC, DMAP, dry CH_2_Cl_2_, reflux, 24 h; (e) NaN_3_, SiCl_4_, MeCN, reflux, 48 h; (f) 1 eq NaSMe, KOH, DMF, reflux, 24 h; (g) 2 eq NaSMe, KOH, DMF, reflux, 24 h; (h) POCl_3_, DMF, 0 °C—rt, 24 h; (i) NH_2_OH·HCl, pyridine, MeOH, reflux, 24 h; (j) Ac_2_O, pyridine, reflux, 48 h.

The synthesis of the new tetrazole derivatives was achieved, as depicted in Scheme 1. Compounds **5**, **6**, and **8** were obtained as previously reported [44], and the amide **7** was obtained following the same procedure as compound **6**—through nucleophilic addition of an amino group from **5** to carboxylic acid **2** in the presence of 1-ethyl-3-(3-dimethylaminopropyl)carbodiimide (EDC) and *N*,*N*-dimethylaminopyridine (DMAP). The amido groups of **6** and **7** were transformed into 1,5-disubstituted tetrazoles, compounds **8** and **9**, respectively, via a reaction with sodium azide and tetrachlorosilane [44]. The introduction of one (**8** → **10**) or two (**10** → **11**) methylsulfanyl groups on the pyridine ring was achieved via a reaction with 1 or 2 equivalents of sodium methanethiolate, respectively. Further modifications on the 3-indole position were accomplished via the Vilsmeier–Haack reaction of the 2,6-disubstituted pyridyl derivatives **8**–**11** to afford the carbaldehyde derivatives **12**–**15**, which were transformed in the oximes **16**–**19** through treatment with hydroxylamine. The oximes were obtained as steroisomeric *Z*+*E* mixtures since the isomerization of isolated isomers was observed. Finally, the carbonitrile derivatives **20**–**23** were synthesized from the oximes **16**–**19** and acetic anhydride in the presence of pyridine. Substitution patterns of 2,6-pyridine moieties were selected according to previous work, where combinations of chlorine with methoxy or methylsulfanyl were shown to benefit antiproliferative activity [54,55]. In a previous work, we reported that the incorporation of a 2,6-dimethoxypyridine moiety as a B ring in isocombretastatin, combretastatin, and phenstatin derivatives led to a loss of antiproliferative activity [55]; therefore, the synthesis of 2,6-dimethoxypyridine derivatives was discarded. The synthetic route is very suited to expand the structural modifications of the tetrazole to optimize their biological and physicochemical properties after structure–activity relationship studies.

### 2.2. Aqueous Solubility

The low aqueous solubility of colchicine site ligands is one of the main obstacles encountered when developing pre-clinical or clinical phases. The most potent combretastatin derivatives usually show water solubilities of about 1–4 µg/mL, whereas one of the most soluble reference compounds, ABT-751, reaches a solubility of 40 µg/mL (Table 1), but its antiproliferative potency decreases up to submicromolar IC_50_ values (see Table 2). One of the aims of the present work was to develop new antimitotic compounds whose increase in antiproliferative potency would be accompanied by high water solubility. In this regard, the substitution of the olefin bridge of CA-4 by tetrazole, as well as the replacement of the trimethoxyphenyl ring with a pyridine moiety, should improve the solubility in water.

The aqueous solubility of tetrazole derivatives was determined by the shaking flask methodology in phosphate buffer at pH 7.4, microfiltration, and quantification of the saturated solutions via UV spectroscopy. All of the compounds showed higher solubility than CA-4, and many of them were more soluble than ABT-751 (Table 1). The introduction of polar groups on the 3-indole position was favorable since the most soluble compounds were the oximes **16**–**19** (38–53 µg/mL), followed by the carbaldehyde series **12**–**15** (33–45 µg/mL), and the carbonitrile derivatives (25–40 µg/mL), whereas the unsubstituted indoles **8**–**11** were the least soluble series (19–35 µg/mL). The replacement of the pyridine chlorine atom by methoxy or methylsulfanyl groups also enhanced aqueous solubility as 2,6-dichloropyridines behaved as the least soluble derivatives within each family (e.g., compare **8** with **9**, **10**, and **11**). 2-Chloro-6-methoxy-pyridines exhibited the highest solubility values for all series (e.g., compound **13** in the carbaldehyde series or compound **21** in the carbonitrile series), whereas the introduction of one or two methylsulfanyl groups afforded intermediate values between the dichloropyridines and the 2-chloro-6-methoxypyridines in each series (e.g., compare **22** and **23** with **20** and **21**). Those results demonstrate that the modifications to lead compound **8** successfully improved water solubility, which could enhance the pharmacokinetic properties of the new molecules with respect to the reference ligands CA-4 and ABT-751. 

### 2.3. Biology

#### 2.3.1. Antiproliferative Activity and Sensitivity to MDR Efflux Pumps

The ability of the synthesized compounds to inhibit the cell proliferation of human cancer cell lines was assayed by measuring cell viability through the use of the MTT method 72 h after treatment. For this purpose, representative cell lines of the most frequent kinds of cancer were selected, including the following: HeLa (cervix epithelioid carcinoma), MCF7 (breast adenocarcinoma), U87 MG (glioblastoma), T98G (temozolomide resistant glioblastoma), HepG2 (hepatocellular carcinoma), HCT8 (colorectal carcinoma), and HT-29 (colon adenocarcinoma). Compounds were also evaluated against the non-tumor cell line HEK-293 (embryonic kidney) to study their selectivity toward cancer cells. Four reference compounds were used: three colchicine-site ligands, CA-4, colchicine, and ABT-751, and TMZ, which is the first-line drug for the treatment of glioblastoma multiforme (Table 2).

All the compounds were able to inhibit the growth of the cancer cell lines in the submicromolar range of IC_50_ values, reaching nanomolar potencies for the most active compounds, thus demonstrating the success of developing this pyridyl-tetrazol-1-yl-indole family of compounds.

Structural modifications to the pyridine ring and the 3-indole position of the lead compound **8** affected the antiproliferative activity in different ways. Regarding the substituents at the indole ring, the best-performing compounds were the carbaldehyde derivatives (compounds **12**–**15**), followed by the non-substituted derivatives at the 3-indol position (compounds **8**–**11**), both series with IC_50_ from the nanomolar to the low submicromolar range, followed by the oxime derivatives (compounds **16**–**19**), showing low submicromolar IC_50_ values, all of them with higher potencies than the reference compound ABT-751. The nitrile derivatives (compounds **20**–**23**) constituted the least potent series since their IC_50_ increased up to the high submicromolar range in most cases. 

Regarding the effect of the functional groups at the pyridine ring, the highest antiproliferative potencies were for the 2-chloro-6-methylsulfanylpyridyl derivatives within every series (compound **10** in the unsubstituted indoles series, compound **14** in the formyl series, compound **18** in the oxime series, and compound **22** in nitrile series), followed by the 2,6-dichloropyridine derivatives (compounds **8**, **12**, **16**, and **20**), and the 2-chloro-6-methoxypyridines (compounds **9**, **13**, **17**, and **21**), whereas the 2,6-dimethylsulfanylpyridines (**11**, **15**, **19**, and **23**) were the least potent derivatives in each series (e.g., compare **15** with **12**, **13**, and **14**, or **11** with **8**, **9**, and **10**). The combination of indole-3-carbaldehyde or 3-unsubstituted indoles with the 2-chloro-6-methylsulfanyl, 2,6-dichloropyridine, or 2-chloro-6-methoxy pyridines afforded the most potent compounds **8**, **9**, **10**, **12**, **13**, and **14**, which exerted cell growth inhibition in the double-digit nanomolar range.

The overall results indicate that one chlorine atom on the pyridine moiety is required for high potency, whereas for the second pyridine substituent, the potency is highest for a methylsulfanyl, followed by a second chlorine atom and then by a methoxy group. The replacement of the two chlorine atoms by two methylsulfanyl groups was detrimental to the antiproliferative activity. This rank order for the antiproliferative activity of the pyridine substituents was maintained for the entirety of the indole 3 substituted series, thus indicating that they maintain the same interactions with the target. Interestingly, this rank order is not the same as that found for a related family of isocombretastatins, phenstatins, and related compounds, probably due to the geometric differences between the two scaffolds shown by the structural studies of the ligands (see below) [53]. Regarding the indole substitutions at the 3 position, the antiproliferative activity improved only for formyl groups, whereas potency decreases were progressively observed for oximes and nitrile substituents. These structure–activity relationships could be accounted for by the interactions between these moieties and the colchicine binding site (suggested by the docking results; see later).

Concerning the effects on the different cell lines, each compound displayed similar IC_50_ values for HeLa, HepG2, and HCT8 cells, whereas the HT-29 cells were less sensitive to the synthesized compounds, a feature that has been already observed for other colchicine site ligands and also the reference compounds CA-4, colchicine, and ABT-751 [57]. The most potent compounds, **10**, **12**, and **14**, reached IC_50_ values of 12–22 nM in U87 MG cells and 19–26 nM in the temozolomide-resistant T98G cells, showing similar potency to that of CA-4, higher potency than colchicine, and much higher potency than TMZ, whose IC_50_ values are over 100 µM in both cell lines, thus suggesting that those compounds could be a good alternative to TMZ.

Multidrug resistance (MDR) proteins, especially P-glycoprotein (P-gp), are often involved in resistance mechanisms affecting tubulin-binding drugs that are frequently related to the failure of cancer therapies. To study if the synthesized compounds behaved as substrates of MDR proteins, we evaluated their antiproliferative activity against the HT-29 cells in the presence of verapamil, a P-gp/MDR1 inhibitor [58], at 10 µM (a concentration that does not affect the cell viability). All the compounds showed similar IC_50_ values in the presence or absence of verapamil, thus indicating that they are not affected by resistance mechanisms based on efflux pumps. These pumps are also part of the blood–brain barrier [59].

The compounds were also tested against the non-tumor HEK-293 cells to study their selectivity toward cancer cells. All compounds exhibited lower cytotoxic activities in HEK-293 compared to the rest of the cancer cell lines, with IC_50_ values in the micromolar range and high selectivity indexes (ratio between IC_50_ in HEK-293 and a tumor cell) since most of the compounds were between 30 and 60 times more cytotoxic for the tumor cells compared to HEK-293 (e.g., compounds **15**–**19**), and the most potent compounds reached selectivity indexes of up to 100–200 (e.g., compounds **8**, **9**, **10**, **12**, and **14**, especially for the GBM cell lines). These results confirmed that the new compounds could display a safe profile, indicating that they have the potential to be used as anticancer drugs.

#### 2.3.2. Tubulin Polymerization Inhibition (TPI)

We evaluated the capacity of the new synthesized compounds to inhibit the polymerization of microtubular protein isolated from calf brains. The assays were performed by taking absorbance measurements to quantify the amount of polymer mass formed in the presence (or absence; negative controls) of the compounds. Overall, 75% of the compounds (compounds **8**–**19**) displayed TPI IC_50_ values lower than the IC_50_ values of ABT-751 and values that were similar to or lower than the IC_50_ values of CA-4 and colchicine (Table 3). Only the less cytotoxic nitrile derivatives (compounds **20**–**23**) showed tubulin inhibition potencies lower than ABT-751. The TPI values were strongly correlated with antiproliferative activity against cancer cells, thus suggesting that the mechanism of action is based on interference with tubulin. This is often not the case for microtubule destabilizing drugs [60], and even closely related series show very different relationships between their quantitative TPI and cytotoxicity IC_50_ values. An example is the difference in cytotoxicity observed between the reference compounds CA-4 and ABT-751 (both had rather similar TPI IC_50_ values). Different uptake, efflux, solubility, and cellular contexts have been proposed to justify these disagreements. The compounds described here have good solubilities, are not substrates of MDR proteins, and still showed a good correlation of TPI and cytotoxicity in different cell lines, thus suggesting that the maintenance of the rank order is due to the binding to tubulin. 

Remarkably, the most potent compound that arrested cell growth (compound **14**), showed the lowest TPI IC_50_ (0.8 µM), with antiproliferative IC_50_ in the nanomolar range. For those analogs with the same di-substituted pyridine ring, formyl derivatives evinced the lowest TPI IC_50_ values, followed by 3-unsubstituted indole derivatives, with oximes and nitrile derivatives showing progressively descending potencies (e.g., compare compounds **8**, **12**, **16**, and **20**, or compounds **10**, **14**, **18**, and **22**). When comparing tubulin inhibition abilities within a series of 3-indol position derivatives, 2-chloro-6-methylsulfanylpyridyl derivatives were the best-performing of each series (compounds **10**, **14**, **18**, and **22**), and 2,6-dimethylsulfanylpyridines were the least potent derivatives of each series (compounds **11**, **15**, **19**, and **23**), while 2,6-dichloro- and 2-chloro-6-methoxypyridines exerted intermediate potencies (e.g., compare series of compounds **8**–**11**, **12**–**15**, **16**–**19**, or **20**–**23**), displaying similar trends to those observed in antiproliferative activity. Interestingly, those compounds showing cell growth inhibition activity in the lower nanomolar range achieved TPI IC_50_ near 1 µM (compounds **10**, **12**, and **14**), which is between five- and two-fold lower than the TPI IC_50_ value of ABT-751 and CA-4. The discrepancy in magnitude order between antiproliferative and TPI IC_50_ is justified by the different nature of each experiment, i.e., the inhibition of cell growth is observable at a low (nanomolar) concentration of compounds while the TPI experiments are based on polymer mass changes that require a high concentration of both proteins and compounds (in the micromolar range). The observed structure–activity relationship for TPI depends on fewer parameters than the cytotoxicity values. Their close parallel further reinforces the assessment that they both reflect the interaction with tubulin. Therefore, the binding modes suggested by the docking experiments and the conformational preferences of the substituents should provide a basis for the observed potency rank order (see later).

#### 2.3.3. Effects on Cellular Microtubules

To elucidate if the interaction of the compounds with tubulin inferred from the in vitro TPI experiments has consequences on cellular microtubules, we studied the effect of representative compounds on the microtubule network of HeLa, MCF7, and U87 MG cells 24 h after treatment via the use of immunofluorescence confocal microscopy through α-tubulin and nuclei labeling.

**Figure 3:**
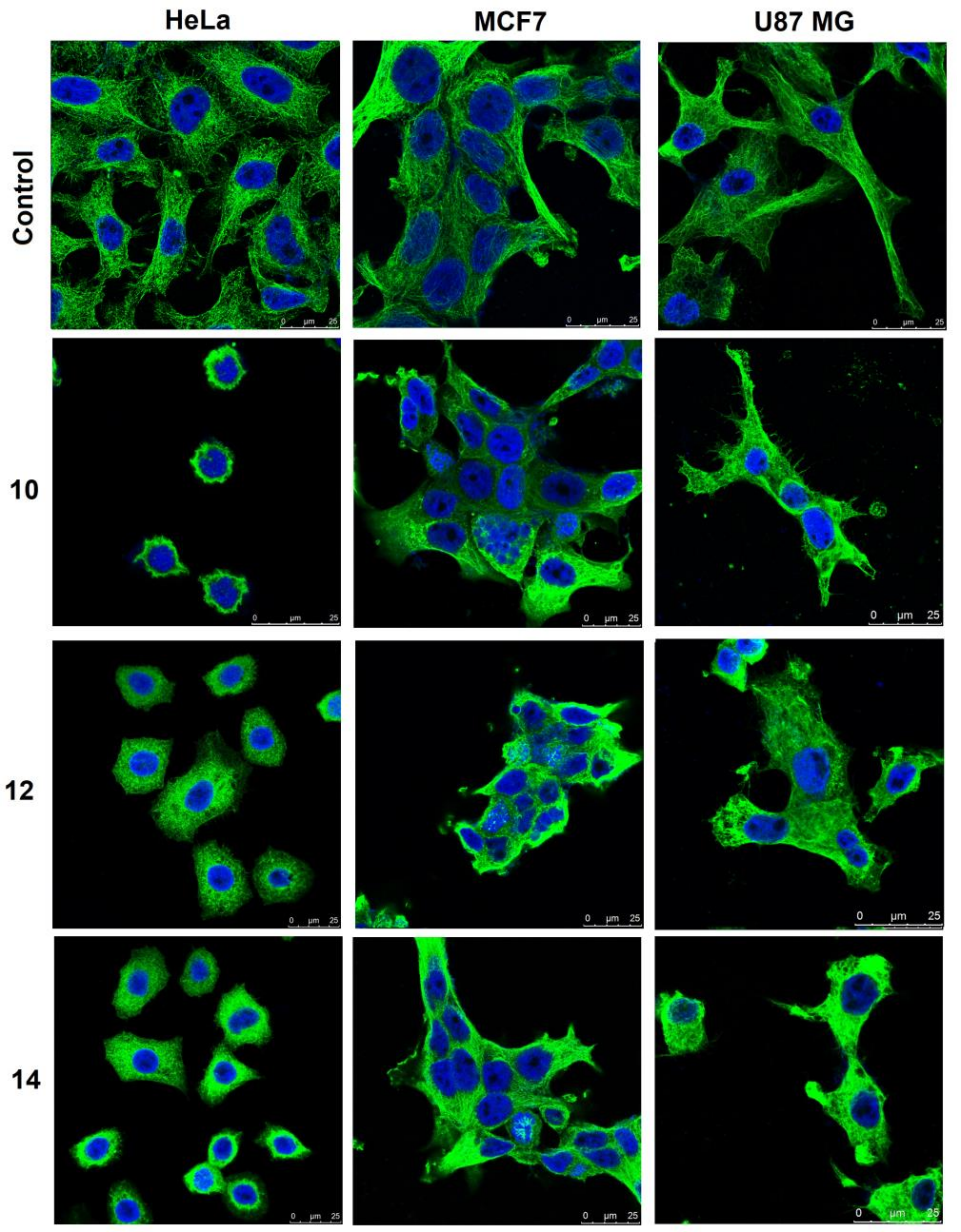
Effects of treatment with compounds **10**, **12**, and **14** on the microtubule network of HeLa, MCF7, and U87 MG cells, as observed via confocal microscopy 24 h after incubations at concentrations of 100 nM for each compound. Untreated cells were used as negative controls. For immunofluorescence labeling, α-tubulin was stained in green, and nuclei were stained in blue. Scale bar: 25 µm.

For these studies, we selected compounds **10**, **12**, and **14** because they exhibited the lowest antiproliferative and TPI IC_50_ values. Both compounds **10** and **14** share the 2-choro-6-methylsulfanylpyridine ring and differ in the substituent at the indole 3 position, whereas compound **12** is an indole-3-carbaldehyde derivative, like **14**, but contains a 2,6-dichloropyridine. In untreated control samples (Figure 3), each kind of tumor cell exhibits a defined morphology with blue-stained nuclei, and well-defined fibers of the microtubule network covering the cytoplasm are shown in green. After HeLa, MCF7, or U87 MG were treated with compounds **10**, **12**, or **14**, at 100 nM, a severe microtubule network disruption was observed in all cases as a green mass lacking structured filaments. Those changes were accompanied by remarkable changes in cell morphology, which were more pronounced in U87 MG cells. Furthermore, multi-lobular nucleated cells were observed for treated MCF7 cells, indicating that mitotic arrest was occurring. These experiments demonstrate that the inhibition of tubulin polymerization observed in vitro also affects cellular microtubules at low concentrations and short time points after treatment.

#### 2.3.4. Effects on the Cell Cycle

To assess how tubulin polymerization inhibition and the effects on the microtubule network affect the cell cycle, flow cytometry experiments were performed on HeLa, MCF7, and U87 MG cells 24, 48, and 72 h after treatments with compounds **10**, **12**, and **14** at 100 nM using propidium iodide (PI) for DNA staining and untreated cells as the negative control.

Untreated HeLa cells showed similar cell cycle profiles at different time points (Table 4, Supplementary Figure S33), with small percentages of cells in the subG0/G1 phase (dead cells), S phase (synthesis), and most of the cells in G0/G1 phase. The three selected compounds were able to induce remarkable mitotic arrest at the early time point of 24 h (around two-fold increases in the number of cells in the G2/M phase with respect to the controls) and also induced cell death, as indicated by the accumulation in the SubG0/G1 region. Mitotic arrest was also observed 48 and 72 h after treatment, but the population corresponding to the G2/M fraction progressively decreased at the same time that the SubG0/G1 population increased until maximal values near 50% were reached. Compounds **10** and **12** exhibited similar profiles, whereas the most potent compound (**14**) induced higher levels of cell death at every time point (in good agreement with its lower antiproliferative IC_50_ value in HeLa cells). 

The histograms of the control MCF7 cells were similar to those of the HeLa cells (Supplementary Figure S34, Table 5). Treatment with compounds **10**, **12**, or **14** also provoked significant mitotic arrest 24 h after treatment, as indicated by the accumulation of cells in the G2/M region, but the induction of cell death was not as high as what was observed for HeLa cells, especially for compounds **10** and **12**, at the same time that the percentage of cells arrested at the mitotic phase was maintained over time. Compound **14** caused a higher accumulation of cells in the subG0/G1 region than the other compounds, but the effect was also below that for HeLa cells. The lower potency of compounds to induce cell death after mitotic arrest in MCF7 cells can be assigned to the deficiency of this cell line in caspase-3, which is essential in the apoptotic cell response [61]. 

The evolution of U87 MG cell cycle profiles after treatment with compounds **10**, **12**, or **14** (Supplementary Figure S35, Table 6) was similar to that observed for HeLa cells in the sense that both mitotic arrest and cell death were induced only 24 h after treatment, and a progressive increase in the SubG0/G1 population accompanied by a decrease in the G2/M fraction occurred at later time points of 48 and 72 h. However, cell death induction was more severe in the case of the U87 MG cells, as indicated by higher SubG0/G1 populations 72 h after treatment (approx. 70%, Table 6) compared to HeLa cells (45–57%, Table 4). These results are in good agreement with the antiproliferative experiments, where U87 MG cells were shown to be more sensitive to the compounds than HeLa and MCF7 cells.

A time-course analysis of the cell cycle confirmed that the inhibition of tubulin polymerization and microtubule network disorganization triggered by the compounds is associated with mitotic arrest, which explains the cell growth inhibition previously quantified via MTT assays. Furthermore, flow cytometry studies allowed us to better understand the meaning of the antiproliferative IC_50_ values since IC_50_ only quantifies the percentage of cell growth inhibition. Conversely, the cell cycle histograms exposed how different levels of mitotic arrest and cell death are present in the different cell lines after treatment with compounds **10**, **12**, or **14**. 

#### 2.3.5. Cell Death Mechanism Studies

To characterize the cell death mechanism induced by the representative compounds **10**, **12**, or **14** at 100 nM on HeLa, MCF7, and U87 MG cells, dual-channel flow cytometry studies were carried out for cells in the absence (negative control) or presence of compounds 72 h after treatment. Fluorescein isothiocyanate-labeled Annexin V (FITC-AnV) and propidium iodide (PI) were used for double staining. Regarding their response to AnV and PI, cells can be identified as viable (PI−, AnV−), early apoptotic (PI−, AnV+), late apoptotic or secondary necrotic (PI+, AnV+), or necrotic (PI+, AnV−). The translocation of the phosphatidylserine from the inner to the outer cell membrane causes the response of apoptotic cells to AnV, whereas only cells suffering some kind of membrane disintegration (late apoptotic or necrotic cells) allow PI permeation.

Untreated HeLa, MCF7, and U87 MG cells exhibited 94–96% of viable cells. High levels of early and late apoptotic death were observed 72 h after HeLa cells were treated with compounds **10**, **12**, or **14** (nearly 73% of apoptosis was induced by compounds **10** and **12**, while 80% was induced by the most potent compound, compound **14**). The percentages of apoptotic cells were higher than those observed in the cell cycle histograms for the SubG0/G1 populations 72 h after treatment, which can be explained by the different nature of the two experiments. Cell cycle analyses do not use a double PI and AnV staining, so there probably were early apoptotic cells that were classified in the G2/M or G0/G1 region due to their DNA content.

**Figure 4:**
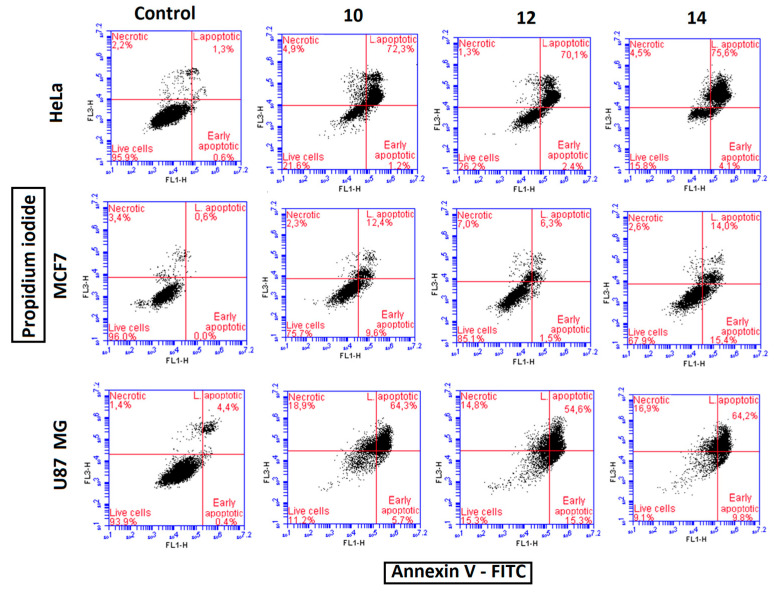
Annexin V-FITC and propidium iodide (PI) profiles in HeLa, MCF7, and U87 MG cells 72 h after treatment with compounds **10**, **12**, or **14** at 100 nM. Untreated cells were used as negative controls.

Apoptotic cell death levels were lower for MCF7 in comparison with HeLa cells, and the values were in good agreement with the subG0/G1 populations observed via cell cycle analysis. A high number of the MCF7 cells encountered at the subG0/G1 region after treatment with compounds **10**, **12**, and **14** (27, 15 and 34%, respectively, Table 5) corresponded to both early and late apoptotic populations (22, 8 and 29%, respectively, Figure 4), whereas the rest of the dead cells suffered necrosis (2.3, 7 and 2.6% for compounds **10**, **12**, and **14**, respectively).

Treated U87 MG cells displayed the highest cell death levels, showing similar profiles for compounds **10**, **12**, and **14**, with 9–15% of live cells and a strong apoptotic response of 70–74%, while necrotic cells accounted for 15–19% of the total population (Figure 4). These results are consistent with the previous findings, indicating the high sensitivity of glioblastoma cells to treatment with antimitotic compounds.

In all cases, the highest potency of compound **14** was evinced since it showed the lowest percentages of live cells in the three cell lines compared to compounds **10** and **12**, which exhibited similar cell death profiles.

### 2.4. Computational Studies

#### 2.4.1. Structural Effects of the A Ring Modifications

**Figure 5:**
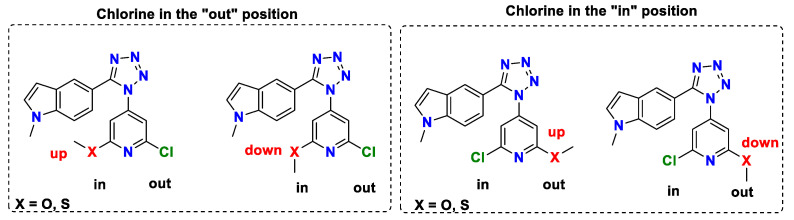
Representation of the different geometries that can be encountered for the 2-chloro-6-methoxy and 2-chloro-6-methylsulfanyl derivatives.

**Figure 6:**
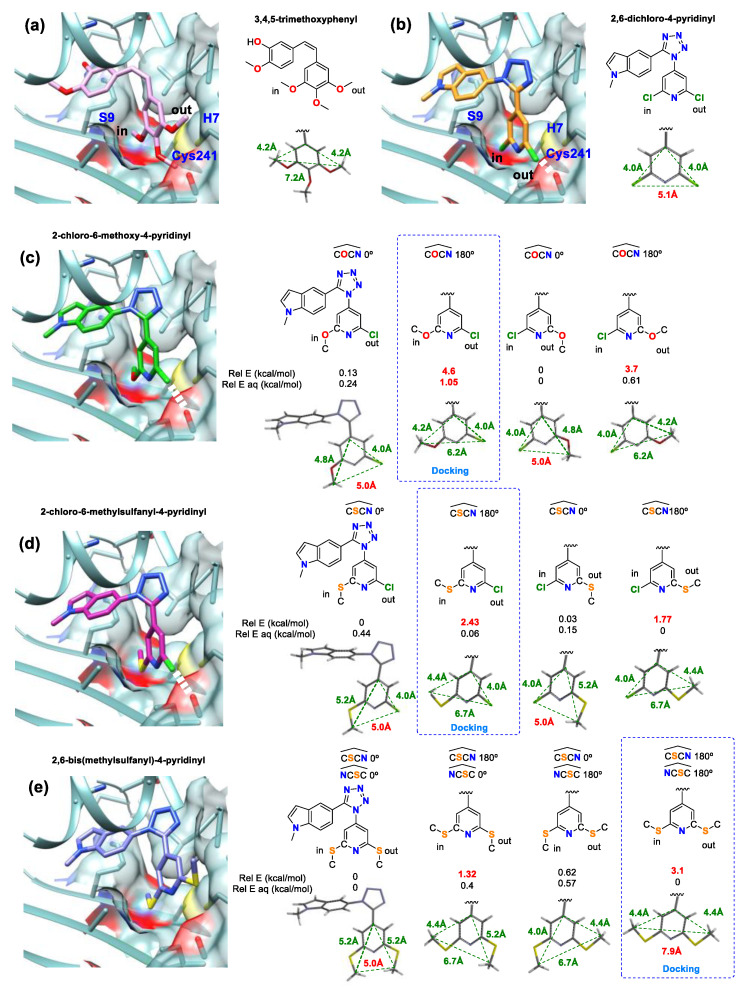
Comparison of the geometries of disubstituted pyridine rings of the tetrazole derivatives and docking poses for the compounds in complex with tubulin in the colchicine domain. The compounds **8**, **9**, **10**, and **11** (panels (**b**), (**c**), (**d**), and (**e**), respectively) are depicted in 2D representations, indicating the “in” and “out” positions of the substituents. The 3D structures of the A rings are represented underneath, and the dihedral angles involving the methoxy and the methylsulfanyl groups are indicated. The green triangles indicate the dimension of the A rings. The relative energies (Rel E, in kcal/mol) are indicated above the most stable geometry (indicated as 0 kcal/mol). The structure and complex in tubulin for CA-4 are also represented (panel (**a**)). The halogen bond between chlorine and the carbonyl oxygen of Val238β (H7) is represented in panels (**b**–**d**).

We studied the structure and the conformational preferences of the diaryltetrazoles via DFT calculations, focusing on the energetic differences between the possible dispositions of the substituents of the pyridine rings as potential determinants of the binding to tubulin. The electrostatic repulsion of the lone pair of the pyridine nitrogen, as well as that of the oxygen atom of the methoxy groups or the sulfur atom of the methylsulfanyl substituents, plays an important role in their preferred conformations. We also observed differences in these preferences depending on the solvent polarity, i.e., water and vacuum, with the second one being used as a surrogate for the non-polar colchicine site. The geometry imposed by the tetrazole on the relative placement of the two heterocyclic systems (the indole and the disubstituted pyridine) results in different environments and energetics for the two pyridine substituents: one closer to the indole (we will refer to it as “in”), and the other opposite to it (we will refer to it as “out”, Figure 5). These two orientations also correspond to different allocations in the complexes of the tetrazoles with tubulin, with the first one (“in”) pointing towards the sidechains of Lys352β and Ala354β of the S9 sheet that forms the wall for the colchicine A site, while the second one (“out”) extends towards the C zone and helix H7, whose Cys241 sidechain favorably makes polar interactions with the pyridine nitrogen (see later, Figure 6). Also, the pyridine substituents with a freely rotating bond (methoxy and methylsulfanyl groups) can adopt two main conformations (Figure 5): one placing the methyl groups along the C4-N1 axis of the ring (we will refer to it as “down” conformation) and another with the methyl group directed towards the perpendicular of this axis at the ring center and in the ring plane (we will refer to it as “up” conformation). As previously described, the first ones (“down” conformations) are usually preferred in 2,6-disubstituted pyridines (see relative energies of the different conformations in Figure 6, where the most stable geometries are “down” conformations). These preferred dispositions are different from those of the methoxy substituents of the 3,4,5-trimethoxyphenyl ring of CA-4, which prefer the “up” conformations (Figure 6a, where only the preferred conformation of CA-4 is shown). 

The docking results show that the conformations adopted in the tetrazole series also condition the interactions with tubulin, thus suggesting that it has an important role in the different observed binding modes. As shown in the subsequent molecular docking section, when the substituents in the pyridine ring are different (2-chloro-6-methoxy and 2-chloro-6-methylsulfanyl derivatives), the preferred docking poses place the chlorine in the “out” position. Regarding the “up” and “down” conformations of the methyl groups, the “up” dispositions are found to be preferable since this ensures that the size of the A ring is more similar to the trimethoxyphenyl ring of CA-4, thus accounting for better interactions in the A zone. The relative energies for the different geometries and the preferred docking poses are depicted in Figure 6.

In this context, the energetics of the system combined with the interactions with tubulin explain the antimitotic potency differences observed for the pyridine substituents (see later). Interestingly, these potency variations also differ from those previously found in one-carbon spaced diaryls [54], which can be explained by the geometries of the scaffolds that, in turn, modify the structure–activity relationships inferred from the two families: tetrazole and one-carbon spaced dyaryls. The tetrazoles enforce a U shape, with the two heteroaromatic rings being closer than in diarylmethanes, which is in accordance with the design objective of adopting a disposition similar to that of the Z isomer of combretastatin A4 (Figure 6). 

We have previously studied the consequences of these preferences on the potency of phenstatin analogs and other one-carbon-atom bridged biaryls inhibitors of tubulin polymerization [53,54,55]. However, these conformational preferences have different effects on the potency of the two-atom bridged tetrazoles, as the former adopt a disposition with a wider angle between the aromatic rings that pushes the A ring towards the walls of the colchicine site, thus making steric hindrance a major determinant of the potency of the tetrazole derivatives.

#### 2.4.2. Docking Studies

We studied the binding modes of compounds **6**–**23** at the colchicine site of tubulin by conducting docking experiments. The many available X-ray crystal structures of colchicine site ligands bound to tubulin have shown that the site is more flexible than initially thought and that it can bind compounds of varied scaffolds and sizes, occupying different sub-pockets even for structurally related ligands [22]. We accounted for the colchicine site flexibility by means of ensemble docking procedures that use an ensemble of X-ray structures of tubulin in complexes with the most diverse set of colchicine site ligands available in the belief that it efficiently samples the different ligand pharmacophore configurations set by the varied protein arrangements. The docking of the virtual ligands in the protein ensemble selects the more complementary combinations of ligand and receptor, efficiently exploring the possible interactional space [43]. We used 112 X-ray crystal structures available at the pdb [22,62], with the optional inclusion of water molecules that make bridges between ligands and the protein site up to a total of 139 structures plus 6 additional ones from a previous molecular dynamics simulation obtained as described [40], to represent the different possible configurations of the colchicine site of tubulin. 

**Figure 7:**
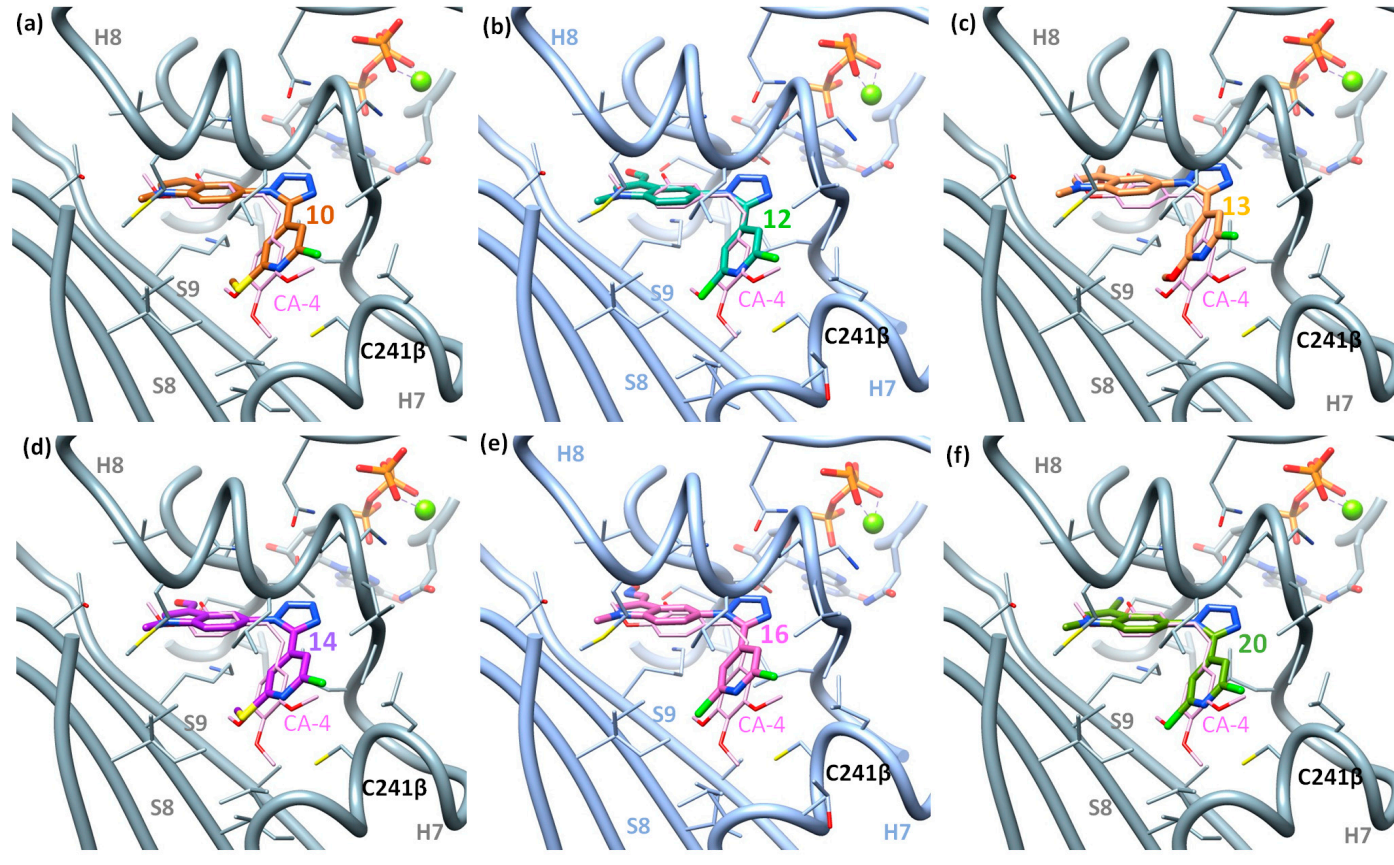
Docking poses for selected compounds in complex with tubulin (**5JVD** in blue and **5H7O** in gray cartoons) superposed onto the X-ray structure of combretastatin A-4 (**CA-4**), which is depicted in light pink: (**a**) Compound **10** (carbons in orange); (**b**) compound **12** (carbons in turquoise); (**c**) compound **13** (carbons in yellow); (**d**) compound **14** (carbons in purple); (**e**) compound **16** (carbons in pink); (**f**) compound **20** (carbons in olive). The structural elements forming the colchicine-site zones are indicated, along with interacting sidechains. The superimposition with the structure of CA-4 shows the high similarity between the binding modes of the two ligands.

We applied two docking programs with very different scoring functions (PLANTS [63] and AutoDock 4.2 [64]]) for the docking experiments. We used the common pose with the best scoring combination for the two docking programs as the binding pose for every docked ligand. The scoring was assessed by automated geometry and energy scoring comparison procedures. For the geometrical comparisons, the following tasks were performed: (a) sub-pocket occupancy assignments were calculated for each ligand pose by measuring the lowest distances to the sub-pocket geometrical centers defined by the pharmacophores derived from the X-ray structures of colchicine site ligands in complex with tubulin; (b) RMSD calculations between the poses and unsubstituted scaffolds with the same bridges that place the phenyl rings at the centers of the A, B, or C sites of the colchicine site of tubulin were executed; and (c) the RMSDs (Root Mean Square Deviations) of the poses with combretastatin A-4 (for A-B binding), MI-181 (for A-C binding), or ABT-751 (for A-B-C binding) in their pdb [65] X-ray structures (pdb IDs 5LYJ, 4YJ2, and 3HKC, respectively [62]) were determined. Every pose for every ligand is allocated to the corresponding sub-pockets according to the above results (see Supplementary Table S2 in Supplementary Materials). To facilitate a comparison between the scores of the two programs, we converted the individual scores to relative scales ranging from 0 (worst) to 1 (best) and calculated Z-scores. For each considered ligand, the pair of poses with the best combination of Z-values for the two programs were assigned as the consensus binding mode. Control experiments with representative colchicine site ligands of known X-ray structures in complex with tubulin correctly retrieved the experimental poses as described, thus validating the applied methodology [66]. Selected examples of the consensus binding poses for ligands **10**, **12**, **13**, **14**, **16**, and **20**, each with different substitutions regarding the indole and the pyridines, are shown in Figure 7.

The ensemble docking approach also provides information about the protein structures selected by the docking procedure, thus offering insight into the sites most complementary to the assayed ligands. In this case, the pdb IDs most frequently retrieved are 5JVD, with a methyl chalcone (2*E*)-3-(3-hydroxy-4-methoxyphenyl)-1-(7-methoxy-2*H*-1,3-benzodioxol-5-yl)-2-methylprop-2-en-1-one analog similar to CA4, and 5H7O, with a 2-(1*H*-indol-4-yl)-4-(3,4,5-trimethoxyphenyl)-1*H*-imidazo[4,5-c]pyridine ligand (Figure S36 in the Supplementary Materials). They have a trimethoxyphenyl ring or related moiety within the A pocket. The proteins in every pair of the consensus poses are usually different for AutoDock, which prefers 5JVD, and PLANTS, which prefers 5H7O. This is probably a reflection of their different scoring functions. These results reinforce the validity of using of ensemble docking strategies with as many protein structures as possible for the docking experiments and validate our application of consensus scoring approaches. The scoring results of neither program cannot discriminate between the subtle differences observed in the biological results. This is expected, considering the accuracy of the energetics accessible in the theoretical calculations, especially if the limitations of the highly simplified scoring functions and the potency, which depends on more events than just binding, are considered.

The docking procedure selected binding poses for all the tetrazoles occupying the B and A zones of the colchicine site in a similar disposition to that observed for the X-ray complex of tubulin with combretastatin A4 (Figure 7). This is due to the U-shaped arrangement of the two heteroaryl systems, a disposition required for the binding to the A-B zone, as has been well-documented for related systems. In all the cases, the indole rings occupy the B zone, contacting helix H8. The bicyclic system contacts flat helix 8 (H8), making carbonyl—py interactions with the sidechain of Asn258β (S8). The substituent on the indole nitrogen protrudes toward residues 179–181 of the α subunit. The aldehydes **12**, **13**, and **14** (Figure 7b–d, respectively) are of a suitable disposition to make hydrogen bonds with the sidechain ammonium group of Lys352β, while oximes (Figure 7e) and nitriles (Figure 7f) protrude more and collide somewhat with the neighboring residues, thus explaining the higher potency of aldehyde derivatives with respect to the oxime and nitrile analogs. The tetrazoles lie in the interfacial space between the two subunits, and the pyridine rings occupy the A zone, acting as replacements for the trimethoxyphenyl ring of combretastatin A4 (Figure 7). The A site has a shape of an inverted table, with sheets S8 and S9 forming the flat tabletop and the sidechains of Ala316β (S8), Ile318β (S8), Lys352β (S9), and Ala354β (S9) protruding as the legs and forming a pocket that the pyridines occupy edgewise, with the pyridine nitrogen facing the flat surface between the sheets and the ring planes crossing from S8 to S9 between the legs. The substituents of the pyridine ring accumulate against the walls of the hole, which is closed by H7 and the loop between helices H7 and H8 [63,67].

Docking scores are not accurate enough to correctly predict the rank order for the compounds, as previously noted for related families [53,54,55]. Therefore we attempted to justify the observed structure–activity relationships for the tetrazole series by jointly considering the docking and conformational studies. The preference for one chlorine ligand on the pyridines suggests that it makes an important interaction with tubulin. The docking results show that the out chlorine atom is located close to the carbonyl oxygen of Val238β (H7), making a halogen bond (Figure 6b–d). The rank order for the antimitotic potency of the “in” substituents can then be accounted for by a combination of conformational preferences and the size of the lateral substituents of the pyridine. With the chlorine in the out disposition, the methoxy and the methylsulfanyl substituents bind in the same “in” dispositions; however, the energetic penalty is much higher for the former, therefore explaining its lower potency (Figure 6c,d). The dichloropyridines do not have conformational penalties, but their smaller size results in slightly poorer packing within the pocket, thus explaining the observed intermediate potency between the other two (Figure 6b). Finally, the pyridine with the two methylsulfanyl substituents lacks the halogen bond and is too wide to fit in the site (Figure 6e), thus explaining the lower potency. These interactions cannot occur in the related diarylmethane series [54] due to the wider angle between the rings enforcing a different interaction of the substituents with the target, thus explaining the observed differences in the structure–activity relationships for these two structurally similar families of colchicine site ligands.

## 3. Materials and Methods

### 3.1. Chemistry

#### 3.1.1. General Chemical Techniques

Reagents were used as purchased without further purification. Solvents (dichloromethane, methanol, acetonitrile, toluene, ethanol, ethyl acetate) were dried and stored using molecular sieves. TLC was performed on precoated silica gel polyester plates (0.25 mm thickness) through the use of a UV fluorescence indicator 254 (Polychrom SI F254). Chromatographic separations were performed on silica gel columns via flash (Kieselgel 40, 0.040–0.063 mm; Merck, Rahway, NJ, USA) chromatography. The compound/stationary phase ratio was 1g of compound/50 g of silica gel. ^1^H NMR and ^13^C NMR spectra were recorded in CDCl_3_, CD_3_OD, DMSO-*d*_6_, or C_3_D_6_O using a Bruker SY spectrometer at 400/100 MHz or a Varian Mercury 400/100 MHz spectrometer. DEPT-135 and DEPT-90 spectra were recorded to identify the C, CH, CH_2_, and CH_3_ signals (Figures S1–S16 in Supplementary Materials). Chemical shifts (δ) are given in ppm downfield from tetramethylsilane, and coupling constants (J values) are given in Hz. IR spectra in KBr disks were run on a Nicolet Impact 410 Spectrophotometer (see Figures S17–S32 in Supplementary Materials). A hybrid QSTAR XL quadrupole/time of flight spectrometer was used for HRMS analyses. The aqueous solubility of the compounds was determined in a Helios Alfa Spectrophotometer from Thermo-Spectronic.

#### 3.1.2. Chemical Synthesis and Characterization

Method A. General synthetic procedure for the preparation of carbaldehyde derivatives (compounds **12**–**15**).

POCl_3_ (6 eq) was added to dry DMF (3 mL) at 0 °C and stirred for 30 min under N_2_ atmosphere. Then, 1 eq of the indole derivative (compound **8**, **9**, **10**, or **11**) was added and the mixture was stirred at rt for 24 h. Then, the reaction was poured into ice water saturated with sodium acetate (10 mL) and kept at 4 °C overnight until precipitation. The resulting powder was filtered, dissolved in CH_2_Cl_2_ (15 mL), and dried over anhydrous Na_2_SO_4_. The solvent was evaporated under vacuum to obtain the carbaldehyde derivative that was purified by crystallization.

Method B. General synthetic procedure for the preparation of oxime derivatives (compounds **16**–**19**).

A solution consisting of the aldehyde derivative (compound **12**, **13**, **14**, or **15**, 1 eq) NH_2_OH·HCl (10 eq) and pyridine (10 eq) was heated in refluxing methanol (20–30 mL) for 24 h under N_2_ atmosphere. Then, the methanol was evaporated under vacuum, and CH_2_Cl_2_ (20–30 mL) was added. The organic layer was washed with NaCl saturated aqueous solution (20 mL, three times) until neutral pH. The organic layer was dried over anhydrous Na_2_SO_4_, filtered, and evaporated under vacuum to obtain a mixture of both oximes (*E+Z*). The products were purified via flash column chromatography.

Method C. General synthetic procedure for the preparation of carbonitrile derivatives (compounds **20**–**23**).

The mixture of *Z+E* oximes (compounds **16**, **17**, **18**, or **19**, 1 eq) was dissolved in pyridine (4 mL), and an excess of acetic anhydride was added (18 eq). The mixture was refluxed for 48 h. The reaction was quenched by pouring onto ice, and the mixture was extracted with CH_2_Cl_2_ (20 mL, twice). The organic layer was washed with 1 M HCl (20 mL, twice), 5% NaHCO_3_ (20 mL, twice), NaCl saturated aqueous solution (20 mL, twice), dried over anhydrous Na_2_SO_4_, filtered, and evaporated under reduced pressure. The products were purified via flash column chromatography.

Compounds **1**, **3**–**6**, and **8** [44] and compound **2** [55] were prepared and purified as previously reported.

2-chloro-6-methoxy-*N*-(1-methyl-1*H*-indol-5-yl)pyridine-3-carboxamide (**7**). A solution of **2** (2.0 g, 10.6 mmol), EDC (1.65 g, 10.6 mmol), and DMAP (1.30 g, 10.6 mmol) in CH_2_Cl_2_ (30 mL) was stirred for 1 h at room temperature (rt). Then, compound **5** (1.10 g, 7.52 mmol) was added, and the mixture was stirred under reflux for 24 h. Subsequently, the mixture was poured into cold water (30 mL), and the organic layer was washed with 1 M HCl (30 mL, twice), NaHCO_3_ (30 mL, twice), NaCl saturated aqueous solution (30 mL, twice), dried over anhydrous Na_2_SO_4_, filtered and the solvent was evaporated under vacuum. The crude was purified via column chromatography (1:3–1:1 EtOAc-Hexane) and crystallized in EtOAc-Hexane to obtain 1.71 g of pure product (5.41 mmol, 72%). ***R*_f_** = 0.25 (1:1 EtOAc-Hexane). M. p.: 187–189 °C. IR (KBr): 3435, 1651, 1604, 1547, 1493 cm^−1^. ^1^H-NMR (400 MHz, CDCl_3_): δ 3.81 (3H, s), 3.93 (3H, s), 6.46 (1H, d, *J* = 5.6 Hz), 7.07 (1H, d, *J* = 3.6 Hz), 7.28–7.33 (3H, m), 7.69 (1H, bs), 7.91 (2H, bs). ^13^C-NMR (100 MHz, C_3_D_6_O): δ 33.0 (CH_3_), 54.8 (CH_3_), 101.6 (CH), 108.6 (CH), 110.6 (CH), 113.4 (CH), 115.3 (CH), 116.6 (CH), 129.4 (C), 130.9 (CH), 131.5 (C), 135.3 (C), 149.5 (C), 149.6 (C), 162.3 (C), 165.3 (C). HRMS (C_16_H_14_N_3_O_2_Cl) *m*/*z*: calculated 338.0663 (M + Na^+^); found 338.0666 (M + Na^+^).

5-(5-(2-chloro-6-methoxypyridin-4-yl)-1*H*-tetrazol-1-yl)-1-methyl-1*H*-indole (**9**). A solution consisting of compound **7** (470 mg, 1.5 mmol) and sodium azide (780 mg, 12.0 mmol) was prepared in acetonitrile (7 mL). Silicon tetrachloride (1.37 mL, 12.0 mmol) was added dropwise, and the mixture was stirred under reflux for 48 h. Then, it was cooled down in an ice bath and the reaction was quenched by dilution with CH_2_Cl_2_ (14 mL) and 5% NaHCO_3_ (20 mL) and vigorously stirred for 30 min. The aqueous layer was extracted with EtOAc (20 mL, twice), and the organic layers were washed with brine (20 mL, twice), then dried over anhydrous Na_2_SO_4_, filtered, and evaporated under reduced pressure. The product was purified via column chromatography (1:3–1:1 EtOAC-Hexane) and crystallized in MeOH to obtain 413 mg of pure product (1.21 mmol, 81%). *R*_f_ = 0.52 (1:1 EtOAc-Hexane). M. p.: 149–150 °C. IR (KBr): 1613, 1551, 1493, 1240, 1155 cm^−1^. ^1^H-NMR (400 MHz, CDCl_3_): δ 3.86 (3H, s), 3.88 (3H, s), 6.56 (1H, d, *J* = 3.6 Hz), 6.72 (1H, d, *J* = 1.2 Hz), 7.12 (1H, dd, *J* = 8.0 Hz, *J* = 1.8 Hz), 7.17 (1H, d, *J* = 1.2 Hz), 7.23 (1H, d, *J* = 3.6 Hz), 7.44 (1H, d, *J* = 8.0 Hz), 7.62 (1H, d, *J* = 1.8 Hz). ^13^C-NMR (100 MHz, CDCl_3_): δ 33.3 (CH_3_), 54.5 (CH_3_), 102.1 (CH), 108.7 (CH), 110.5 (CH), 115.2 (CH), 118.2 (CH), 118.3 (CH), 125.6 (C), 128.6 (C), 131.7 (CH), 136.2 (C), 137.1 (C), 149.5 (C), 150.1 (C), 163.9 (C). HRMS (C_16_H_13_N_6_OCl) *m*/*z*: calculated 341.0908 (M + H^+^); found 341.0912 (M + H^+^).

5-(5-(2-chloro-6-(methylsulfanyl)pyridin-4-yl)-1*H*-tetrazol-1-yl)-1-methyl-1*H*-indole (**10**). Sodium methanethiolate (85 mg, 1.23 mmol) and KOH (35 mg, 0.6 mmol) were added to a solution of compound **8** (419 mg, 1.21 mmol) in dry DMF (20 mL), and the mixture was refluxed for 24 h. Then, the mixture was cooled down, and the solvent was evaporated under vacuum. The crude was dissolved in EtOAc (20 mL), and the organic layer was washed with 1 M HCl (20 mL) and NaCl saturated aqueous solution (20 mL, twice), dried over anhydrous Na_2_SO_4_, filtered, and evaporated under reduced pressure. The product was purified via column chromatography (7:3 EtOAC-Hex) and crystallized in MeOH to obtain 332 mg (0.93 mmol, 77%). *R*_f_ = 0.57 (1:1 EtOAc-Hexane). M. p.: 167–168 °C. IR (KBr): 2922, 1541, 1433, 1301 cm^−1^. ^1^H-NMR (400 MHz, CDCl_3_): δ 2.44 (3H, s), 3.90 (3H, s), 6.60 (1H, dd, *J* = 0.9 Hz, *J* = 2.0 Hz), 7.14 (1H, dd, *J* = 2.0 Hz, *J* = 8.0 Hz), 7.15 (1H, d, *J* = 2 Hz), 7.24 (1H, s), 7.26 (1H, s), 7.46 (1H, d, *J* = 8.0 Hz), 7.65 (1H, d, *J* = 2.0 Hz). ^13^C-NMR (100 MHz, CDCl_3_): δ 13.5 (CH_3_), 33.3 (CH_3_), 102.3 (CH), 110.6 (CH), 117.6 (CH), 118.2 (CH), 118.3 (CH), 118.5 (CH), 125.4 (C), 128.7 (C), 131.8 (CH), 134.1 (C), 137.2 (C), 151.0 (C), 151.7 (C), 162.7 (C). HRMS (C_16_H_13_N_6_SCl) *m*/*z*: calculated 357.0683 (M + H^+^); found 357.0684 (M + H^+^).

5-(5-(2,6-bis(methylsulfanyl)pyridin-4-yl)-1*H*-tetrazol-1-yl)-1-methyl-1*H*-indole (**11**). Compound **11** was synthesized following the same methodology as compound **10**, i.e., by using a solution consisting of compound **10** (560 mg, 1.6 mmol) in dry DMF (30 mL), sodium methanethiolate (224 mg, 3.20 mmol), and KOH (45 mg, 0.8 mmol). The product was purified via column chromatography (7:3 EtOAC-Hexane) and crystallized in MeOH to obtain 524 mg of compound **11** (1.42 mmol, 89%). *R*_f_ = 0.61 (1:1 EtOAc-Hexane). M. p.: 160–161 °C. IR (KBr): 2926, 1593, 1434, 1312, 1300 cm^−1^. ^1^H-NMR (400 MHz, CDCl_3_): δ 2.46 (6H, s), 3.89 (3H, s), 6.58 (1H, dd, *J* = 0.9 Hz, *J* = 3.2 Hz), 6.99 (2H, s), 7.15 (1H, dd, *J* = 2.1 Hz, *J* = 8.8 Hz), 7.23 (1H, d, *J* = 3.2 Hz), 7.45 (1H, d, *J* = 8.8 Hz), 7.65 (1H, d, *J* = 2.1 Hz). ^13^C-NMR (100 MHz, CDCl_3_): δ 13.5 (2CH_3_), 33.3 (CH_3_), 102.2 (CH), 110.4 (CH), 115.0 (2CH), 118.3 (CH), 118.4 (CH), 125.9 (C), 128.6 (C), 131.6 (CH), 131.7 (C), 137.1 (C), 151.7 (C), 161.0 (2C). HRMS (C_17_H_16_N_6_S_2_) *m*/*z*: calculated 369.0944 (M + H^+^); found 369.0950 (M + H^+^).

5-(5-(2,6-dichloropyridin-4-yl)-1*H*-tetrazol-1-yl)-1-methyl-1*H*-indole-3-carbaldehyde (**12**). Obtained as described in method A from DMF (3 mL), POCl_3_ (716 µL, 7.83 mmol), and compound **8** (450 mg, 1.30 mmol). The crude was crystallized in Et_2_O to obtain 451 mg of pure product (1.21 mmol, 93%). M. p.: 198–199 °C. IR (KBr): 2925, 1661, 1538, 1434 cm^−1^. ^1^H-NMR (400 MHz, CDCl_3_): δ 3.93 (3H, s), 7.46 (1H, dd, *J* = 2.1 Hz, *J* = 8.8 Hz), 7.63 (2H, s), 7.78 (1H, d, *J* = 8.8 Hz), 8.33 (1H, d, *J* = 2.1 Hz), 8.45 (1H, s), 9.89 (1H, s). ^13^C-NMR (100 MHz, DMSO-*d*_6_): δ 33.9 (CH_3_), 112.5 (CH), 117.3 (C), 118.9 (CH), 121.0 (CH), 123.1 (2CH), 124.6 (C), 128.1 (C), 138.1 (C), 138.3 (C), 143.8 (CH), 149.9 (2C), 150.0 (C), 184.9 (CH). HRMS (C_16_H_10_N_6_OCl_2_) *m*/*z*: calculated 395.0184 (M + Na^+^); found 395.0185 (M + Na^+^).

5-(5-(2-chloro-6-methoxypyridin-4-yl)-1*H*-tetrazol-1-yl)-1-methyl-1*H*-indole-3-carbaldehyde (**13**). Obtained as described in method A from DMF (3 mL), POCl_3_ (662 µL, 7.08 mmol), and compound **9** (402 mg, 1.18 mmol). The crude was crystallized in Et_2_O to obtain 379 mg of pure product (1.02 mmol, 87%). M. p.: 214–215 °C. IR (KBr): 2944, 1659, 1461, 1433, 1220 cm^−1^. ^1^H-NMR (400 MHz, CDCl_3_): δ 3.79 (3H, s), 3.95 (3H, s), 6.83 (1H, d, *J* = 1.2 Hz), 7.26 (1H, d, *J* = 1.2 Hz), 7.50 (1H, dd, *J* = 2.1 Hz, *J* = 8.8 Hz), 7.79 (1H, d, *J* = 8.8 Hz), 8.33 (1H, d, *J* = 2.1 Hz), 8.48 (1H, s), 9.91 (1H, s). ^13^C-NMR (100 MHz, DMSO-*d*_6_): δ 34.2 (CH_3_), 54.8 (CH_3_), 109.8 (CH), 112.8 (CH), 116.1 (CH), 117.6 (C), 119.2 (CH), 121.6 (CH), 124.9 (C), 128.8 (C), 137.6 (C), 138.5 (C), 144.0 (CH), 148.4 (C), 151.6 (C), 163.7 (C), 185.2 (CH). HRMS (C_17_H_13_N_6_O_2_Cl) *m*/*z*: calculated 369.0860 (M + H^+^); found 369.0861 (M + H^+^).

5-(5-(2-chloro-6-(methylsulfanyl)pyridin-4-yl)-1*H*-tetrazol-1-yl)-1-methyl-1*H*-indole-3-carbaldehyde (**14**). Obtained as described in method A from DMF (3 mL), POCl_3_ (510 µL, 5.46 mmol), and compound **10** (324 mg, 0.91 mmol). The crude was crystallized in Et_2_O to obtain 315 mg of pure product (0.82 mmol, 90%). M. p.: 201–202 °C. IR (KBr): 2938, 1671, 1448, 1435, 1211 cm^−1^. ^1^H-NMR (400 MHz, CDCl_3_): δ 2.48 (3H, s), 3.89 (3H, s), 7.59–7.61 (3H, m), 7.77 (1H, dd, *J* = 2.1 Hz, *J* = 8.8 Hz), 8.25 (1H, bs), 8.42 (1H, bs), 9.89 (1H, s). ^13^C-NMR (100 MHz, CDCl_3_): δ 13.8 (CH_3_), 33.9 (CH_3_), 110.4 (CH), 116.0 (CH), 117.0 (C), 117.6 (CH), 117.8 (CH), 121.2 (CH), 124.7 (C), 128.3 (C), 136.2 (C), 136.8 (C), 143.6 (CH), 149.5 (C), 151.0 (C), 161.8 (C), 184.4 (CH). HRMS (C_17_H_13_N_6_OSCl) *m*/*z*: calculated 385.0633 (M + H^+^); found 385.0634 (M + H^+^).

5-(5-(2,6-bis(methylsulfanyl)pyridin-4-yl)-1*H*-tetrazol-1-yl)-1-methyl-1*H*-indole-3-carbaldehyde (**15**). Obtained as described in method A from DMF (3 mL), POCl_3_ (755 µL, 8.01 mmol), and compound **11** (496 mg, 1.34 mmol). The crude was crystallized in Et_2_O to obtain 456 mg of pure product (1.15 mmol, 86%). M. p.: 207–208 °C. IR (KBr): 2927, 1665, 1440, 1432, 1219 cm^−1^. ^1^H-NMR (400 MHz, CDCl_3_): δ 2.45 (6H, s), 3.88 (3H, s), 7.59 (1H, dd, *J* = 2.1 Hz, *J* = 8.8 Hz), 7.78 (2H, s), 7.81 (1H, dd, *J* = 2.1 Hz, *J* = 8.8 Hz), 8.20 (1H, bs), 8.40 (1H, bs), 9.89 (1H, s). ^13^C-NMR (100 MHz, CDCl_3_): δ 13.7 (2CH_3_), 33.7 (CH_3_), 111.0 (CH), 115.1 (2CH), 117.2 (C), 119.5 (CH), 121.3 (CH), 125.1 (C), 128.1 (C), 137.5 (C), 137.9 (C), 143.3 (CH), 151.3 (C), 160.5 (2C), 185.3 (CH). HRMS (C_18_H_16_N_6_OS_2_) *m*/*z*: calculated 397.0900 (M + H^+^); found 397.0904 (M + H^+^).

5-(5-(2,6-dichloropyridin-4-yl)-1*H*-tetrazol-1-yl)-1-methyl-1*H*-indole-3-carbaldehyde oxime (**16**). Obtained as described in method B from compound **12** (290 mg, 0.84 mmol), NH_2_OH·HCl (583 mg, 8.4 mmol), and pyridine (676 µL, 8.4 mmol) in MeOH (20 mL). The product was purified via flash column chromatography (1:4–1:1 AcOEt-Hexane) to obtain 221 mg of the *Z+E* mixture of 16 (0.57 mmol, 68%). *R*_f_ = 0.20 (4:3 EtOAc-Hexane). IR (KBr): 3337, 1601, 1541, 1523, 1433 cm^−1^. ^1^H-NMR (400 MHz, CDCl_3_): δ 3.92 (3H, s), 3.93 (3H, s), 7.14–7.70 (6H, m), 7.43–7.60 (6H, m), 7.67 (1H, bs), 7.88 (1H, bs), 8.24 (1H, bs, 8.29), (1H, bs), 8.37 (1H, bs). ^13^C-NMR (100 MHz, CDCl_3_): δ 34.8 (CH_3_), 110.3 (C), 112.0 (CH), 118.3 (CH), 120.0 (CH), 123.8 (2CH), 125.8 (C), 128.6 (C), 136.1 (C), 137.0 (C), 137.7 (CH), 148.1 (CH), 151.7 (2C), 152.4 (C). HRMS (C_16_H_11_N_7_O_2_Cl_2_) *m*/*z*: calculated 388.0475 (M + H^+^); found 388.0475 (M + H^+^).

5-(5-(2-chloro-6-methoxypyridin-4-yl)-1*H*-tetrazol-1-yl)-1-methyl-1*H*-indole-3-carbaldehyde oxime (**17**). Obtained as described in method B from compound **13** (270 mg, 0.73 mmol), NH_2_OH·HCl (507 mg, 7.3 mmol) and pyridine (587 µL, 7.3 mmol) in MeOH (20 mL). The product was purified via flash column chromatography (1:4–1:1 AcOEt-Hexane) to obtain 191 mg of the *Z+E* mixture of **17** (0.51 mmol, 69%). *R*_f_ = 0.17 (4:3 EtOAc-Hexane). IR (KBr): 3272, 1613, 1551, 1522, 1465, 1219 cm^−1^. ^1^H-NMR (400 MHz, CDCl_3_): δ 3.88 (3H, s), 3.96 (3H, s), 7.17–7.22 (3H, m), 7.29 (1H, d, *J* = 2.12 Hz),7.40–7.56 (4H, m), 7.66 (1H, d, *J* = 3.1 Hz), 8.22 (1H, d, *J* = 3.1 Hz), 8.28 (1H, bs), 8.37 (1H, bs), 8.62 (1H, bs). ^13^C-NMR (100 MHz, DMSO-*d*_6_): δ 33.9 (CH_3_), 54.3 (CH_3_), 110.4 (C), 111.7 (CH), 112.8 (CH), 117.7 (CH), 119.1 (CH), 121.3 (CH), 124.6 (C), 128.4 (C), 137.0 (CH), 139.0 (C), 139.2 (C), 148.6 (CH), 149.7 (C), 151.1 (C), 162.0 (C). HRMS (C_17_H_14_N_7_O_2_Cl) *m*/*z*: calculated 384.0970 (M + H^+^); found 384.0974 (M + H^+^).

5-(5-(2-chloro-6-(methylsulfanyl)pyridin-4-yl)-1*H*-tetrazol-1-yl)-1-methyl-1*H*-indole-3-carbaldehyde oxime (**18**). Obtained as described in method B from compound **14** (291 mg, 0.76 mmol), NH_2_OH·HCl (521 mg, 7.5 mmol), and pyridine (603 µL, 7.5 mmol) in MeOH (20 mL). The product was purified via flash column chromatography (1:3–1:1 AcOEt-Hexane) to obtain 224 mg of the *Z + E* mixture of **18** (0.56 mmol, 74%). *R*_f_ = 0.22 (4:3 EtOAc-Hexane). IR (KBr): 3269, 1625, 1548, 1520, 1460, 1225 cm^−1^. ^1^H-NMR (400 MHz, CDCl_3_): δ 3.81 (3H, s), 3.95 (3H, s), 7.05 (1H, s), 7.25–7.35 (3H, m), 7.49 (2H, m), 7.95 (*1H*, s, minor), 8.11 (1H, s), 8.66 (1H, bs). ^13^C-NMR (100 MHz, CDCl_3_): δ 13.8 (CH_3_), 33.9 (CH_3_), 107.4 (CH), 110.8 (C), 111.3 (CH), 118.2 (CH), 119.5 (CH), 120.8 (CH), 125.1 (C), 128.3 (C), 137.4 (CH), 138.6 (C), 139.0 (C), 148.0 (CH), 150.2 (C), 151.7 (C), 163.0 (C). HRMS (C_17_H_14_N_7_OSCl) *m*/*z*: calculated 400.0742 (M + H^+^); found 400.0744 (M + H^+^).

5-(5-(2,6-bis(methylsulfanyl)pyridin-4-yl)-1*H*-tetrazol-1-yl)-1-methyl-1*H*-indole-3-carbaldehyde oxime (**19**). Obtained as described in method B from compound **15** (355 mg, 0.90 mmol), NH_2_OH·HCl (626 mg, 9.0 mmol), and pyridine (725 µL, 9.0 mmol) in MeOH (30 mL). The product was purified via flash column chromatography (1:4–1:1 AcOEt-Hexane) to obtain 267 mg of the *Z+E* mixture of **19** (0.65 mmol, 72%). *R*_f_ = 0.25 (4:3 EtOAc-Hexane). IR (KBr): 3251, 1619, 1545, 1522, 1463, 1219 cm^−1^. ^1^H-NMR (400 MHz, C_3_D_6_O): δ 2.48 (6H, s), 3.98 (3H, s), 7.23–7.47 (4H, m), 7.49–7.54 (3H, m), 7.97 (*1H*, s, minor), 8.12 (1H, s), 8.75 (1H, bs). ^13^C-NMR (100 MHz, CDCl_3_): δ 13.8 (2CH_3_), 33.9 (CH_3_), 110.7 (C), 111.2 (CH), 115.4 (2CH), 119.7 (CH), 120.4 (CH), 125.1 (C), 128.2 (C), 136.6 (CH), 137.1 (C), 139.1 (C), 148.1 (CH), 152.0 (C), 160.6 (2C). HRMS (C_18_H_17_N_7_OS_2_) *m*/*z*: calculated 412.1009 (M + H^+^); found 412.1013 (M + H^+^).

5-(5-(2,6-dichloropyridin-4-yl)-1*H*-tetrazol-1-yl)-1-methyl-1*H*-indole-3-carbonitrile (**20**). Obtained as described in method C from compound **16** (92 mg, 0.24 mmol), acetic anhydride (406 µL, 4.30 mmol) in pyridine (4 mL). The product was purified via flash column chromatography (1:4–1:1 AcOEt-Hexane) to obtain 72 mg of pure product (0.19 mmol, 89%). *R*_f_ = 0.40 (1:1 EtOAc-Hexane). IR (KBr): 2221, 1577, 1548, 1520, 1460 cm^−1^. ^1^H-NMR (400 MHz, CDCl_3_): δ 3.94 (3H, s), 7.19 (2H, s), 7.23 (1H, dd, *J* = 1.6 Hz, *J* = 8.8 Hz), 7.56 (1H, d, *J* = 8.8 Hz), 7.76 (1H, s), 7.80 (1H, d, *J* = 1.6 Hz). ^13^C-NMR (100 MHz, DMSO-*d*_6_): δ 33.5 (CH_3_), 85.1 (C), 112.0 (CH), 115.0 (C), 118.2 (CH), 119.7 (CH), 124.1 (2CH), 126.0 (C), 128.1 (C), 136.2 (C), 136.7 (CH), 138.1 (C), 149.7 (2C), 151.1 (C). HRMS (C_16_H_9_N_7_Cl_2_) *m*/*z*: calculated 392.0189 (M + Na^+^); found 392.0191 (M + Na^+^).

5-(5-(2-chloro-6-methoxypyridin-4-yl)-1*H*-tetrazol-1-yl)-1-methyl-1*H*-indole-3-carbonitrile (**21**). Obtained as described in method C from compound **17** (120 mg, 0.31 mmol), acetic anhydride (533 µL, 5.60 mmol) in pyridine (4 mL). The product was purified via flash column chromatography (1:4–1:1 AcOEt-Hexane) to obtain 105 mg of pure product (0.28 mmol, 92%). *R*_f_ = 0.39 (1:1 EtOAc-Hexane). IR (KBr): 2221, 1589, 1460, 1437, 1222 cm^−1^. ^1^H-NMR (400 MHz, CDCl_3_): δ 3.89 (3H, s), 3.90 (3H, s), 6.72 (1H, d, *J* = 1.2 Hz), 7.13 (1H, d, *J* = 1.2 Hz), 7.31 (1H, dd, *J* = 2.0 Hz, *J* = 8.8 Hz), 7.59 (1H, d, *J* = 8.8 Hz), 7.77 (1H, s), 7.84 (1H, d, *J* = 2.0 Hz). ^13^C-NMR (100 MHz, DMSO-*d*_6_): δ 33.5 (CH_3_), 54.8 (CH_3_), 85.0 (C), 110.0 (CH), 112.4 (CH), 115.2 (C), 117.0 (CH), 119.4 (CH), 122.2 (CH), 124.3 (C), 128.2 (C), 134.1 (CH), 137.0 (C), 137.4 (C), 151.0 (C), 151.4 (C), 162.2 (C). HRMS (C_17_H_12_N_7_OCl) *m*/*z*: calculated 388.0684 (M + Na^+^); found 388.0688 (M + Na^+^).

5-(5-(2-chloro-6-(methylsulfanyl)pyridin-4-yl)-1*H*-tetrazol-1-yl)-1-methyl-1*H*-indole-3-carbonitrile (**22**). Obtained as described in method C from compound **18** (180 mg, 0.45 mmol), acetic anhydride (766 µL, 8.10 mmol) in pyridine (4 mL). The product was purified via flash column chromatography (1:4–1:1 AcOEt-Hexane) to obtain 160 mg of pure product (0.42 mmol, 93%). *R*_f_ = 0.45 (1:1 EtOAc-Hexane). IR (KBr): 2221, 1590, 1454, 1440, 1211 cm^−1^. ^1^H-NMR (400 MHz, CDCl_3_): δ 2.53 (3H, s), 4.01 (3H, s), 7.15 (1H, d, *J* = 1.2 Hz), 7.35 (1H, dd, *J* = 2.0 Hz, *J* = 8.8 Hz), 7.42 (1H, d, *J* = 1.2 Hz), 7.59 (1H, d, *J* = 8.8 Hz), 7.77 (1H, s), 7.81 (1H, d, *J* = 2.0 Hz). ^13^C-NMR (100 MHz, CDCl_3_): δ 13.7 (CH_3_), 32.0 (CH_3_), 85.0 (C), 107.1 (CH), 115.1 (C), 116.3 (CH), 118.7 (CH), 118.8 (CH), 121.3 (CH), 124.3 (C), 128.0 (C), 131.8 (C), 136.6 (C), 137.8 (CH), 151.0 (C), 151.5 (C), 161.4 (C). HRMS (C_17_H_12_N_7_SCl) *m*/*z*: calculated 404.0406 (M + Na^+^); found 404.0408 (M + Na^+^).

5-(5-(2,6-bis(methylsulfanyl)pyridin-4-yl)-1*H*-tetrazol-1-yl)-1-methyl-1*H*-indole-3-carbonitrile (**23**). Obtained as described in method C from compound **19** (230 mg, 0.56 mmol), acetic anhydride (530 µL, 5.60 mmol) in pyridine (4 mL). The product was purified via flash column chromatography (1:4–1:1 AcOEt-Hexane) to obtain 198 mg of pure product (0.50 mmol, 90%). *R*_f_ = 0.49 (1:1 EtOAc-Hexane). IR (KBr): 2217, 1582, 1461, 1438, 1220 cm^−1^. ^1^H-NMR (400 MHz, CDCl_3_): δ 2.48 (6H, s), 3.77 (3H, s), 7.21 (1H, dd, *J* = 2.0 Hz, *J* = 8.8 Hz), 7.51 (2H, s), 7.68 (1H, d, *J* = 8.8 Hz), 8.01 (1H, s), 8.21 (1H, d, *J* = 2.0 Hz). ^13^C-NMR (100 MHz, CDCl_3_): δ 13.4 (2CH_3_), 33.0 (CH_3_), 85.3 (C), 111.2 (CH), 115.2 (C), 115.9 (2CH), 118.0 (CH), 120.7 (CH), 125.3 (C), 128.3 (C), 134.0 (C), 136.1 (CH), 137.9 (C), 151.2 (C), 160.6 (2C). HRMS (C_18_H_15_N_7_S_2_) *m*/*z*: calculated 416.0723 (M + Na^+^); found 416.0723 (M + Na^+^).

### 3.2. Determination of Aqueous Solubility

The aqueous solubility of the compounds was determined using the saturation shake-flask method [54]. First, calibration lines at the maximum wavelength of absorbance were determined for each compound. Then, 5 mg of each compound was suspended in 500 µL of pH 7.4 buffer, and the resulting mixture was shaken for 72 h at room temperature and filtrated over a 45 µm filter to remove the insoluble fraction. The concentration of the compound in the supernatant (saturated solution) was determined by measuring UV absorbance at the maximum wavelength of absorbance and interpolation in the calibration line. A Helios Alfa Spectrophotometer was used for the UV measurements. The aqueous solubility values are given as the average of three independent experiments.

### 3.3. Biology

#### 3.3.1. Cell Culture Conditions

The cell lines were obtained from ATTC (Manassas, VA, USA). HeLa (human cervix epithelioid carcinoma), MCF7 (human breast adenocarcinoma), U87 MG (human glioblastoma), T98G (TMZ-resistant human glioblastoma), HepG2 (human hepatocellular carcinoma), and HEK-293 (human embryonic kidney) were grown in Dulbecco’s modified Eagle’s medium (DMEM, Gibco, Waltham, MA, USA) supplemented with 10% (*v*/*v*) heat-inactivated fetal bovine serum (HIFBS, Sigma-Aldrich, St. Louis, MO, USA), 2 mM L-glutamine, 100 μg/mL streptomycin, and 100 units/mL penicillin (Gibco, Waltham, MA, USA) at 37 °C, 95% humidity, and 5% CO_2_. The HT-29 and HCT8 (human colon carcinoma) cell lines were grown in Roswell Park Memorial Institute 1640 (RPMI 1640) (Gibco, Whaltman, MA, USA) supplemented with 10% HIFBS, 100 μg/mL streptomycin, and 100 units/mL penicillin (Gibco, Whaltman, MA, USA) at 37 °C, 95% humidity, and 5% CO_2_. The cells were periodically tested for *Mycoplasma* infection by using a MycoAlert kit (Lonza, Norwest, Australia), and only *Mycoplasma*-free cells were used in the experiments.

#### 3.3.2. Cell Growth Inhibition Assay

The effect of the compounds on the proliferation of human tumor cell lines was determined by using a MTT reagent ((3-[4,5-dimethylthiazol-2-yl]-2,5-diphenyltetrazolium bromide, MTT Thiazolyl Blue Tetrazolium Bromide, Sigma-Aldrich, St. Louis, MO, USA), which was dissolved in PBS at 5 mg/mL in accordance with the manufacturer’s instructions. Cells were incubated in 96-well plates (100 µL/well) in complete DMEM or RPMI 1640 medium (see above) at 37 °C and 5% CO_2_ atmosphere for 24 h to allow for cell attachment to the plate at the following concentrations: 30,000 cells/mL (U87 MG, T98G, HepG2, HCT8, HT-29, and HEK-293 cells) or 15,000 cells/mL (HeLa and MCF7 cells). Then, every compound was added (10 µL/well) to a final concentration of 10 µM. Untreated cells were used as negative controls. The antiproliferative activity of compounds was measured 72 h after drug exposure through the MTT assay. The IC_50_ value (the drug concentration required to inhibit 50% of cell growth with respect to the untreated control) was determined for compounds showing antiproliferative effects in the initial screening at 10 µM. For this purpose, compounds were used at different concentrations, ranging from 10^−10^ to 10^−2^ M. Measurements were performed in triplicate, and each experiment was repeated three times. The IC_50_ values were determined using Origin software (version 9.6, OriginLab, Washington, DC, USA). To study the sensitivity to MDR efflux pumps of compounds, the IC_50_ values were determined following the same procedure but in the presence of verapamil (final concentration of 10 µM).

#### 3.3.3. Tubulin Polymerization Inhibition

Bovine brain tubulin was isolated as previously described by following a modified version of the Shelanski method [68]. The protein was stored at −80 °C, and its concentration was determined by employing the Bradford method before each use. The experiments were performed using pH 6.7 buffer containing 0.1 M MES, 1 mM β-ME, 1 mM EGTA, 1 mM MgCl_2_, 1.5 mM GTP, 1.5 mg/mL of microtubular protein, and the measured ligand (0.1–10 µM). Control samples had the same composition but in absence of the ligand. The samples were incubated at 20 °C for 30 min to allow the ligands to bind to tubulin and cooled in ice for 10 min to make sure that polymerization did not take place at the initial time point. After reaching a stable plateau, the temperature was adjusted to 4 °C before being shifted to 37 °C to facilitate the assembly process of tubulin. Tubulin polymerization, caused when heating the samples, was accompanied by an increase in the absorbance (at 450 nm) of the sample that was monitored by using a Helios α spectrophotometer. The increase in absorbance obtained for the control sample was considered as 100% of tubulin polymerization (0% of inhibition), so a comparison with the increase in absorbance determined for samples containing ligands yielded the degree of tubulin polymerization inhibition (TPI), which is expressed as a percentage value. The IC_50_ for TPI (the drug concentration required to inhibit 50% of tubulin polymerization with respect to the control) was determined by measuring TPI at different concentrations of ligands (0.1–10 µM) and by performing calculations using Origin software (version 9.6, OriginLab, Washington, DC, USA). Measurements were performed in triplicate in two independent experiments using microtubular proteins from different preparations.

#### 3.3.4. Immunofluorescence Experiments

HeLa, MCF7, and U87 MG cells (8·10^4^ cells/mL) were grown on 0.01% poly-L-lysine pre-coated square glass coverslips (22 mm^2^), deposited on 6-well plates (1 coverslip/well), and incubated in complete DMEM medium (see above) at 37 °C and 5% CO_2_ atmosphere for 24 h. Then, the cells were incubated for 24 h in the presence or absence (negative controls) of selected compounds (**10**, **12**, or **14**) at 100 nM. After drug treatment incubation, medium was removed, and coverslips were washed three times with PBS, fixed with 4% formaldehyde in PBS for 10 min, permeabilized with 0.5% Triton X-100, (Sigma-Aldrich, St. Louis, MO, USA) in PBS for 90 s at 4 °C, blocked with 10% BSA in PBS for 30 min, and washed with PBS four times. The coverslips were incubated for 1 h with anti-α-tubulin mouse monoclonal antibody (Sigma-Aldrich, St. Louis, MO, USA), diluted 1:200 in PBS containing 3% BSA, washed four times with PBS, and then incubated in darkness with the fluorescent secondary antibody Alexa Fluor 488 goat anti-mouse IgG (Molecular Probes, Invitrogen, Eugenen, OR, USA) and diluted 1:400 in PBS containing 1% BSA for 1.5 h. The coverslips were washed four times with PBS, and a drop of ProLong™ Gold Antifade Mountant containing DAPI (ThermoFisher, Waltham, MA, USA) was added for cell nuclei staining. The samples were analyzed via confocal microscopy, which was performed by using a LEICA SP5 microscope DMI-6000V model coupled to a LEICA LAS AF software computer.

#### 3.3.5. Cell Cycle Analysis

HeLa, MCF7, or U87 MG cells were seeded in 6-well plates (8·10^4^ cells/mL, 2 mL/well) and incubated in complete DMEM Medium (see above) at 37 °C and 5% CO_2_ atmosphere for 24 h. Then, the cells were treated with selected compounds (**10**, **12**, or **14**) at 100 nM, and untreated cells were used as negative controls. Cells were collected 24, 48, or 72 h following treatment and fixed overnight in ethanol/PBS (7:3) at 4 °C. After two washes with PBS, cells were suspended in PBS and incubated in darkness overnight with 0.2 mg/mL RNase A (Sigma-Aldrich, St. Louis, MO, USA), 50 µg/mL propidium iodide (Sigma-Aldrich, St. Louis, MO, USA), and Triton 10x at room temperature. A BD Accuri™ C6 Plus Flow Cytometer (BD Biosciences, Franklin Lakes, NJ, USA) cytometer was used to analyze the samples, and BD Accuri™ C6 Software (version 1.0.264.21) was used for data analysis.

#### 3.3.6. Apoptotic Cell Death Quantification

Following the manufacturer’s guidelines, an Annexin V-FITC/PI apoptosis detection kit (Immunostep, Salamanca, Spain) was used to quantify HeLa, MCF7, or U87 MG cells. Cells were seeded in 12-well plates (5·10^4^ cells/mL, 1 mL/well) and incubated in complete DMEM medium (see above) at 37 °C and 5% CO_2_ atmosphere for 24 h. Then, the cells were treated with selected compounds (**10**, **12**, or **14**) at 100 nM, and untreated cells were used as negative controls. Then, 72 h after incubation, the cells were harvested, centrifugated, resuspended in the Annexin V binding buffer, and incubated in darkness with Annexin V-FITC/PI for 15 min at room temperature. The samples were analyzed by using a BD Accuri™ C6 Plus Flow Cytometer (BD Biosciences), and acquired data were analyzed via BD Accuri™ C6 Software (version 1.0.264.21).

### 3.4. Computational Studies

The preferred conformations of the tetrazoles were calculated by means of RB3LYP DFT calculations at the 6–31G(D) level using Spartan 08 software package. The indole rings were kept unsubstituted at the 3-position to avoid long-range interactions that might have confounded the conformational searches. The structures were built and subjected to conformational searches at the molecular mechanism (MMFF force field) level. The retrieved conformations were then minimized by using RB3LYP DFT calculations at the 6–31G(D) level, and the lowest energy configurations amongst all the possible outcomes were selected as the most stable forms for every compound.

Ensemble docking studies were performed as previously described, with the incorporation of a higher number of protein structures and with and without water molecules. The different tubulin–ligand complexes sampled the protein conformational space and helped measure the protein flexibility [52,69]. Briefly, the ligands were docked into the colchicine sites of 145 different tubulin structures from complexes of tubulin with colchicine site ligands deposited in the pdb and five representative structures from a previous molecular dynamics simulation run on a tubulin–podophyllotoxin complex. Parallel docking studies (10 runs per ligand) were performed using PLANTS with default settings and AutoDock 4.2 using the Lamarckian genetic algorithm (LGA) 100−300 times for a maximum of 2.5 million energy evaluations, 150 individuals, and a maximum of 27,000 generations. All the possible configurations for the oximes were used in the docking runs, and the best scored one for every ligand was selected as the docking result. The scores of the different programs were converted into Z-scores to rank them independently from the used scoring scales. The common poses for the two programs with the best consensus Z-scores were selected as the docking results. Every pose was automatically assigned to the colchicine subzones using in-house KNIME pipelines [70]. The RMSDs between all of the poses and model scaffolds with no substituents and with the colchicine binding site ligands representative of binders occupying different subzones were calculated using LigRMSD [71]. Docked poses were analyzed using Chimera [72], Marvin [73], OpenEye [74], and JADOPPT [75].

## 4. Conclusions

Starting from an initial hit from an ensemble pharmacophore search for colchicine site ligands, a new family of diheteroaryl compounds has been designed, synthesized, and evaluated as antitumor compounds. The novel family of tetrazole-1-yl indole derivatives was prepared following an efficient synthetic methodology that is very suitable for the incorporation of further modifications on the two tetrazole-pending heteroaryl fragments. The best-performing candidates were compounds with a formyl group on the 3-indole position and the 2-chloro-6-methylsulfanyl-pyridine derivatives. All the synthesized derivatives were able to exert antiproliferative effects in the submicromolar range against a broad panel of cancer cells, and the most potent compounds (compounds **8**, **9**, **10**, **12**, **13**, and **14**) showed nanomolar IC_50_ values, exhibiting particularly high potencies against the U87 MG glioblastoma cells and even against the TMZ-resistant T98G cells. To the best of our knowledge, this family represents one of the few examples of colchicine-site ligands where the high antimitotic potency is accompanied by high solubility. The synthesized compounds successively overcame the typical limitations of tubulin ligands such as low selectivity toward cancer cells, as evidenced by the fact that they exhibited selectivity indexes from 30 to 200 and the appearance of resistance mechanisms. Different and complementary techniques have been employed to elucidate the mechanism of action of the three representative compounds (**10**, **12**, and **14**). The strong correlation between tubulin polymerization inhibition IC_50_ and the antiproliferative IC_50_ together with the severe microtubule network disruption of treated cells (observed via confocal microscopy) proved that the interaction with tubulin is the mechanism at play. The effects on the cell microtubules 24 h after treatment also affected the cell cycle of the cancer cells, as revealed by the cell cycle histograms obtained 24, 48, and 72 h after treatment. The following common pattern was noted in all of the cases: the compounds were able to provoke severe mitotic arrest at an early time point (24 h), and the G2/M population decreases progressively at the expense of the increase in the subG0/G1 population, which reached its maximum value 72 h after treatment, showing good agreement with the results obtained in the antiproliferative experiments. Our studies of the cell death mechanism were consistent with the cell cycle assays and demonstrated apoptotic induction caused by the compounds. The DFT and docking experiments in the colchicine site of tubulin not only predicted a binding mode of synthesized compounds similar to CA-4 but also accounted for the observed structure–activity relationships. The best-performing ligands were those containing a formyl group at the 3-indole position, as they proved that they were able to make hydrogen bonds with the sidechain ammonium group of Lys352β, which is not encountered for oximes and nitriles derivatives. Regarding the effect of the substituents on the pyridine ring, the superiority of the ligands with chlorine atoms can be explained by their ability to make a halogen bond with the carbonyl oxygen of Val238β (H7), which is not possible for the least potent bismethylsulfanyl derivatives. The conformational penalties calculated for the methoxy derivatives account for their weaker interactions in comparison with 2,6-dichloro- and 2-chloro-6-methylsulfanylpyridine analogs, whereas the smaller size of 2,6-dichloropyridines explain their less efficient packing capacities compared to the 2-chloro-6-methylsulfanylpyridine counterparts. Overall, our results further justify the scientific interest in this class of tetrazole derivatives, and further research could be directed toward the development of new anti-GBM therapies targeting tubulin. Future in vivo experiments are necessary to explore the potential of these new molecules. The formyl group at the 3-indole position could help to improve the pharmacokinetic or pharmacodynamic properties of drugs developed for treatment by serving as an anchor for further derivatization. The described compounds are potential candidates for further clinical development, especially for the treatment of difficult-to-treat glioblastomas.

## Figures and Tables

**Table 1:** Experimental aqueous solubility in phosphate buffer at pH 7.4.

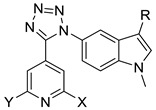				
Nº	X	Y	R	Solubility ^1^ (µg/mL)
**8**	Cl	Cl	H	19.3 ± 0.1
**9**	Cl	OMe	H	35.4 ± 0.2
**10**	Cl	SMe	H	26.1 ± 0.2
**11**	SMe	SMe	H	30.7 ± 0.3
**12**	Cl	Cl	CHO	33.1 ± 0.3
**13**	Cl	OMe	CHO	45.3 ± 0.4
**14**	Cl	SMe	CHO	36.9 ± 0.3
**15**	SMe	SMe	CHO	41.2 ± 0.4
**16**	Cl	Cl	CHNOH	38.2 ± 0.4
**17**	Cl	OMe	CHNOH	53.0 ± 0.3
**18**	Cl	SMe	CHNOH	42.7 ± 0.5
**19**	SMe	SMe	CHNOH	47.8 ± 0.6
**20**	Cl	Cl	CN	25.3 ± 0.1
**21**	Cl	OMe	CN	39.7 ± 0.4
**22**	Cl	SMe	CN	31.2 ± 0.3
**23**	SMe	SMe	CN	34.1 ± 0.3
CA-4				1.1 ± 0.1
ABT-751				40 ± 0.3

^1^ Aqueous solubility was measured by employing the shake-flask method in buffered media at pH 7.4. Data are shown as the mean and standard deviation of three independent experiments.

**Table 2:** Antiproliferative activity against human tumor cell lines and the non-tumorigenic cell line HEK-293, expressed as the IC_50_ in µM, determined via the MTT assay ^1^.

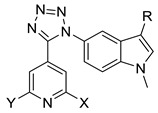												
Nº	X	Y	R	HeLa	MCF7	U87 MG	T98G	HepG2	HCT8	HT-29	HT-29 *	HEK-293
**8**	Cl	Cl	H	0.045	0.043	0.032	0.041	0.049	0.049	0.052	0.052	4.72
**9**	Cl	OMe	H	0.072	0.069	0.048	0.054	0.070	0.075	0.081	0.080	5.20
**10**	Cl	SMe	H	0.039	0.038	0.022	0.025	0.037	0.040	0.059	0.061	4.39
**11**	SMe	SMe	H	0.233	0.240	0.209	0.219	0.227	0.236	0.255	0.257	6.82
**12**	Cl	Cl	CHO	0.036	0.037	0.017	0.026	0.036	0.040	0.051	0.056	3.08
**13**	Cl	OMe	CHO	0.057	0.065	0.055	0.054	0.055	0.050	0.064	0.067	3.72
**14**	Cl	SMe	CHO	0.024	0.026	0.012	0.019	0.031	0.029	0.031	0.033	2.27
**15**	SMe	SMe	CHO	0.144	0.130	0.115	0.122	0.137	0.141	0.244	0.242	6.48
**16**	Cl	Cl	CHNOH	0.207	0.210	0.131	0.127	0.220	0.219	0.211	0.215	7.01
**17**	Cl	OMe	CHNOH	0.220	0.215	0.155	0.181	0.229	0.227	0.238	0.230	7.25
**18**	Cl	SMe	CHNOH	0.131	0.123	0.093	0.102	0.127	0.115	0.152	0.150	6.13
**19**	SMe	SMe	CHNOH	0.307	0.339	0.275	0.294	0.341	0.333	0.355	0.359	8.31
**20**	Cl	Cl	CN	0.625	0.631	0.522	0.555	0.634	0.628	0.679	0.682	>10
**21**	Cl	OMe	CN	0.722	0.701	0.634	0.689	0.740	0.705	0.749	0.756	>10
**22**	Cl	SMe	CN	0.341	0.329	0.312	0.359	0.327	0.335	0.355	0.357	>10
**23**	SMe	SMe	CN	0.841	0.963	0.831	0.865	0.877	0.822	0.929	0.927	>10
ABT-751				0.388	0.180	0.375	0.421	0.327	0.209	0.213	0.250	1.23
CA-4				0.002	0.001	0.010	0.017	0.013	0.220	0.305	0.327	0.721
Colchicine				0.009	0.010	0.031	0.037	0.029	0.291	0.375	0.302	0.801
TMZ				>10	>10	135	241	>10	>10	>10	>10	>10

^1^ IC_50_ is the required drug concentration to inhibit the growth of the corresponding cell line by 50% with respect to the untreated cells (negative controls), 72 h after drug exposure. * IC_50_ determined in the human colon adenocarcinoma HT-29 cell line in the presence of Verapamil (10 µM), a P-gp/MDR1 inhibitor. Data are shown as the mean of three independent experiments performed in triplicate. Standard deviations were, in all cases, below 1% of the mean values (see Supplementary Table S1 in Supplementary Materials).

**Table 3:** Effect of the compounds on tubulin polymerization inhibition (TPI).

Nº	X	Y	R	TPI IC_50_ (µM) ^1^
**8**	Cl	Cl	H	2.8 ± 0.1
**9**	Cl	OMe	H	3.1 ± 0.2
**10**	Cl	SMe	H	1.5 ± 0.1
**11**	SMe	SMe	H	3.8 ± 0.1
**12**	Cl	Cl	CHO	1.3 ± 0.1
**13**	Cl	OMe	CHO	2.8 ± 0.2
**14**	Cl	SMe	CHO	0.8 ± 0.1
**15**	SMe	SMe	CHO	3.5 ± 0.2
**16**	Cl	Cl	CHNOH	3.7 ± 0.2
**17**	Cl	OMe	CHNOH	3.8 ± 0.1
**18**	Cl	SMe	CHNOH	3.3 ± 0.1
**19**	SMe	SMe	CHNOH	4.3 ± 0.2
**20**	Cl	Cl	CN	6.2 ± 0.3
**21**	Cl	OMe	CN	6.9 ± 0.3
**22**	Cl	SMe	CN	4.6 ± 0.2
**23**	SMe	SMe	CN	7.9 ± 0.2
ABT-751				4.4 ± 0.1
CA-4				2.8 ± 0.4
Colchicine				1.4 ± 0.1

^1^ IC_50_ is the concentration to inhibit 50% of tubulin polymerization in vitro with respect to the negative control. Data are shown as the mean and standard deviation of two independent experiments performed in triplicate.

**Table 4:** Analysis of the cell cycle populations (SubG0/G1, G0/G1, S, and G2/M) of Hela cells after 24, 48, or 72 h of treatment with compounds **10**, **12**, or **14** at 100 nM. Untreated control cells were run in parallel.

Time after Treatment	Cell Cycle Population	Control (% Cells)	Compound 10 (% Cells)	Compound 12 (% Cells)	Compound 14 (% Cells)
24 h	SubG0/G1	1.2	18.6	17.8	32.7
	G0/G1	72.0	18.1	20.0	16.6
	G2/M	23.1	59.5	57.8	47.0
	S	3.9	3.9	4.6	3.7
48 h	SubG0/G1	1.6	29.1	32.5	41.3
	G0/G1	72.3	16.7	18.2	17.1
	G2/M	22.6	45.9	40.8	34.1
	S	3.6	7.6	7.8	7.0
72 h	SubG0/G1	0.5	45.6	45.4	57.4
	G0/G1	73.7	14.4	15.3	9.6
	G2/M	22.9	35.2	34.0	25.4
	S	3.0	4.9	5.6	8.1

**Table 5:** Analysis of the cell cycle populations (SubG0/G1, G0/G1, S, and G2/M) of MCF7 cells after 24, 48, or 72 h of treatment with compounds **10**, **12**, or **14** at 100 nM. Untreated control cells were run in parallel.

Time after Treatment	Cell Cycle Population	Control (% Cells)	Compound 10 (% Cells)	Compound 12 (% Cells)	Compound 14 (% Cells)
24 h	SubG0/G1	1.0	7.7	5.2	5.2
	G0/G1	60.3	22.5	19.4	22.7
	G2/M	29.8	65.8	68.6	66.0
	S	9.3	3.9	7.0	5.9
48 h	SubG0/G1	1.2	20.4	13.1	17.9
	G0/G1	59.7	16.9	16.4	16.8
	G2/M	30.8	58.1	62.9	58.4
	S	8.6	4.6	7.9	6.9
72 h	SubG0/G1	3.8	27.2	15.5	34.6
	G0/G1	64.7	13.7	15.0	19.1
	G2/M	23.0	54.8	59.1	38.7
	S	9.1	4.4	10.8	8.1

**Table 6:** Analysis of the cell cycle populations (SubG0/G1, G0/G1, S, and G2/M) of U87 MG cells after 24, 48, or 72 h of treatment with compounds **10**, **12**, or **14** at 100 nM. Untreated control cells were run in parallel.

Time after Treatment	Cell Cycle Population	Control (% Cells)	Compound 10 (% Cells)	Compound 12 (% Cells)	Compound 14 (% Cells)
24 h	SubG0/G1	3.8	23.5	26.1	27.2
	G0/G1	70.5	21.1	27.3	23.7
	G2/M	20.5	47.8	37.8	41.3
	S	5.4	8.3	9.6	8.5
48 h	SubG0/G1	5.9	49.2	51.1	45.4
	G0/G1	72.5	11.0	11.9	12.5
	G2/M	17.0	31.6	27.4	32.1
	S	4.7	9.0	10.3	10.6
72 h	SubG0/G1	1.5	69.6	70.6	72.6
	G0/G1	74.3	10.5	12.2	11.1
	G2/M	19.6	17.0	10.0	9.6
	S	4.8	2.9	7.8	6.9

## Data Availability

The data presented in this study are available in Supplementary Materials.

## Supplementary Materials

The following supporting information can be downloaded at: https://www.mdpi.com/article/10.3390/ijms241311093/s1.
