# Supplementary material for: Novel Tetrazole Derivatives Targeting Tubulin Endowed with Antiproliferative Activity against Glioblastoma Cells
Source: Int J Mol Sci. 2023 Jul 4;24(13):11093. doi: 10.3390/ijms241311093 (PMC10342533; doi:10.3390/ijms241311093)
Supplement: Supplementary file 1 [file ijms-24-11093-s001.zip › ijms-2483134-supplementary.pdf]

Supplementary material

Novel Tetrazole Derivatives Targeting Tubulin Endowed with Antiproliferative Activity against Glioblastoma Cells

Laura Gallego-Yerga [1,2,3,*], Andrea Jazmín Chiliquinga [4], and Rafael Peláez [1,2,3,*]

[1] Laboratorio de Química Orgánica y Farmacéutica, Departamento de Ciencias Farmacéuticas, Facultad de Farmacia, Universidad de Salamanca, Campus Miguel de Unamuno, 37007, Salamanca, Spain
[2] Instituto de Investigación Biomédica de Salamanca (IBSAL), Facultad de Farmacia, Universidad de Salamanca, Campus Miguel de Unamuno, 37007, Salamanca, Spain
[3] Centro de Investigación de Enfermedades Tropicales de la Universidad de Salamanca (CIETUS), Facultad de Farmacia, Universidad de Salamanca, Campus Miguel de Unamuno, 37007, Salamanca, Spain
[4] Grupo de Investigación de Ciencias en Red, Universidad Técnica del Norte, Ibarra, 100105, Ecuador; ajchiliquinga1@utn.edu.ec
* Correspondence: gallego@usal.es (L.G.-Y.); pelaez@usal.es (R.P.)

NMR spectra of compounds **7,** and **9** -**23**……………………………………………………………………………………………2

IR spectra of compounds **7,** and **9** -**23**……………………………………………………………………………………………18

Supplementary table S1………..26

Histograms of the cell cycle distribution……………………………………………………………………………………..27

Supplementary Figure S36………29

Supplementary Table S2…….30

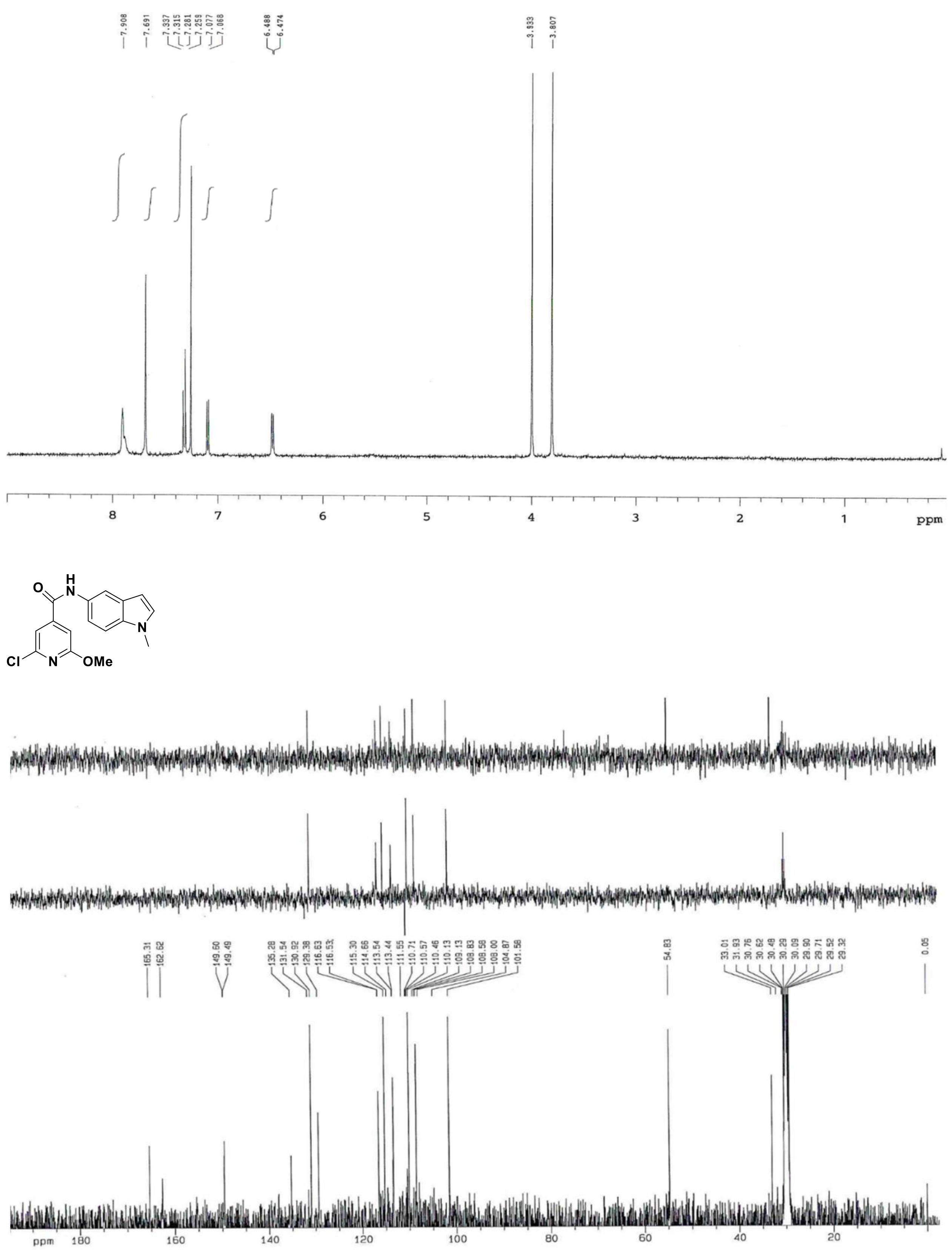

Figure S1. ^{1}H NMR ($CDCl_3$, 400 MHz) and ^{13}C (C_3D_6O, 100 MHz) spectrum of compound **7**. DEPT-135 and DEPT-90 ^{13}C NMR spectrum were recorded to identify the signals corresponding to CH_3, CH, and C.

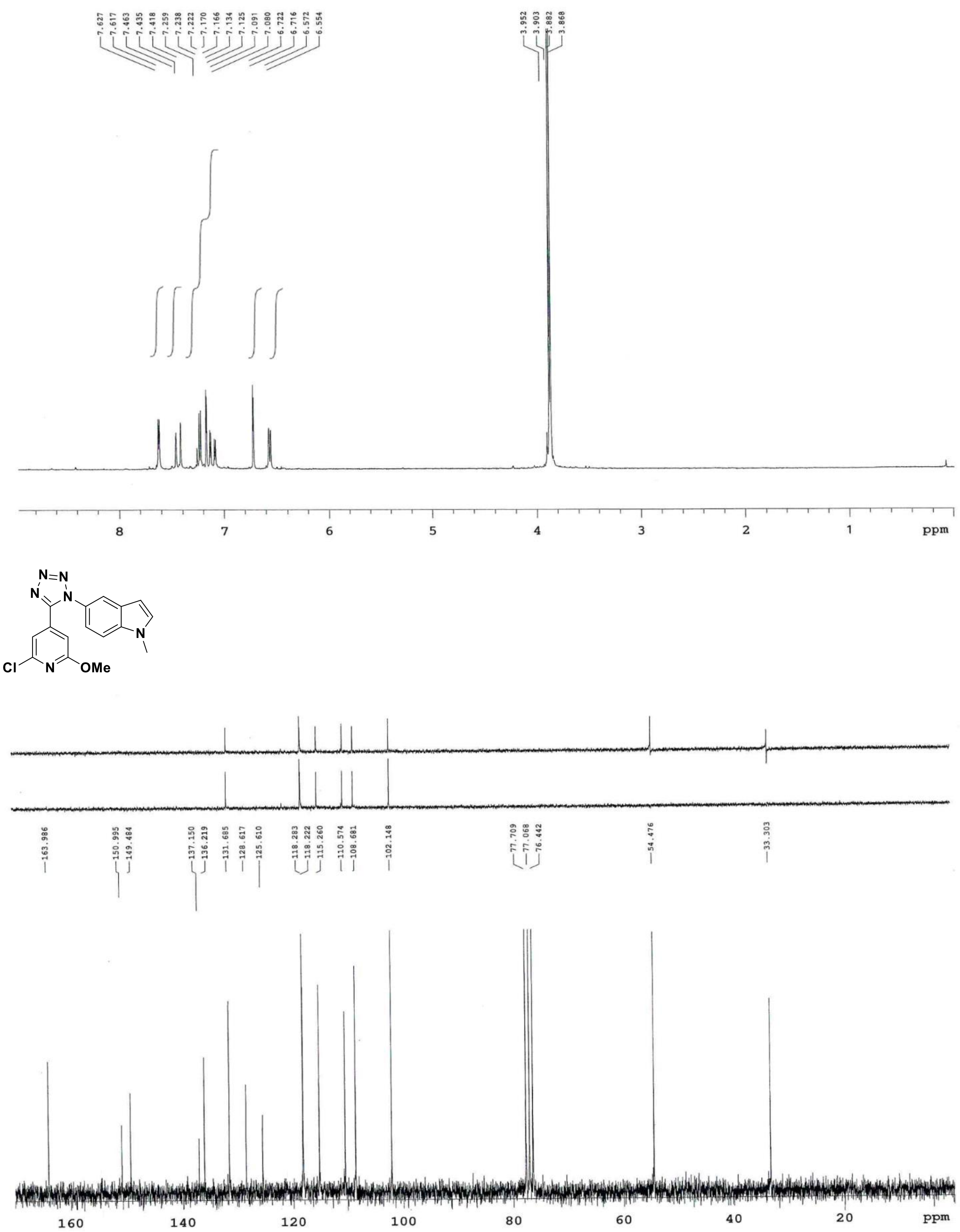

Figure S2. ^{1}H NMR ($CDCl_3$, 400 MHz) and ^{13}C ($CDCl_3$, 100 MHz) spectrum of compound **9**. DEPT-135 and DEPT-90 ^{13}C NMR spectrum were recorded to identify the signals corresponding to CH_3, CH, and C.

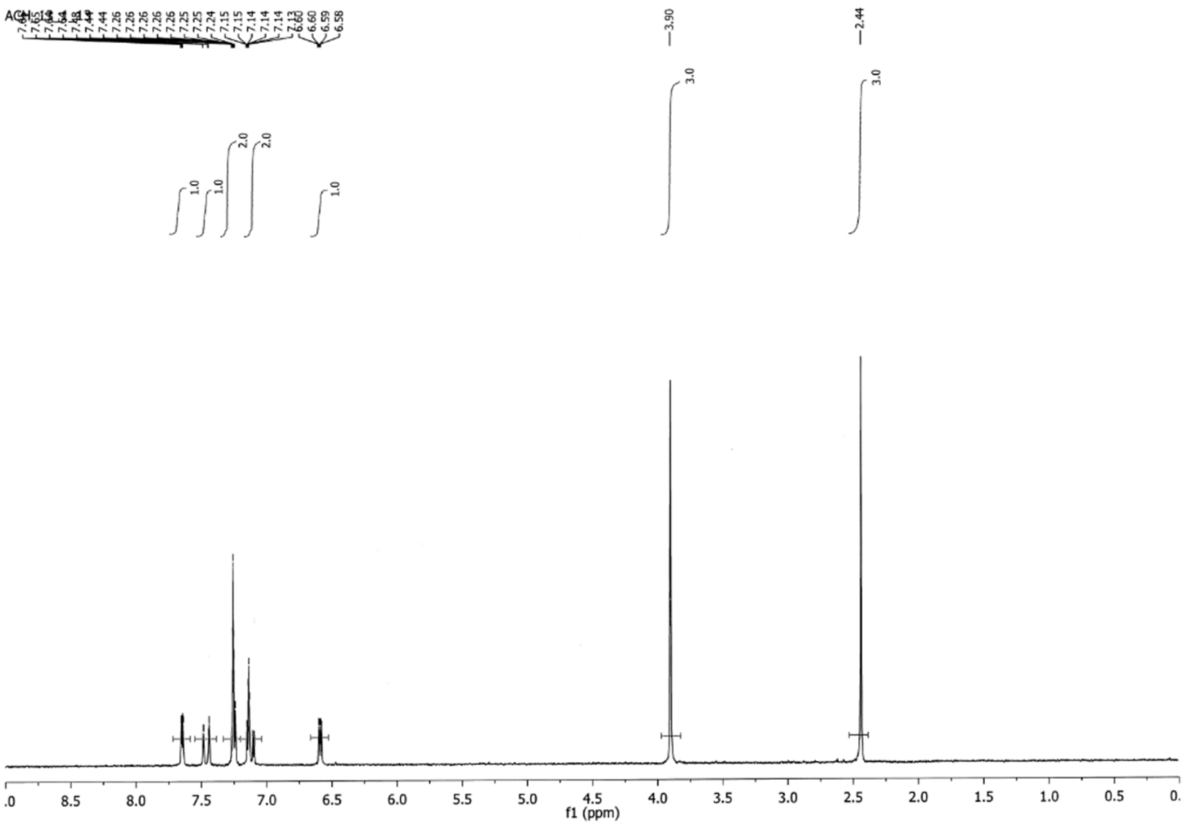

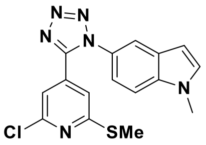

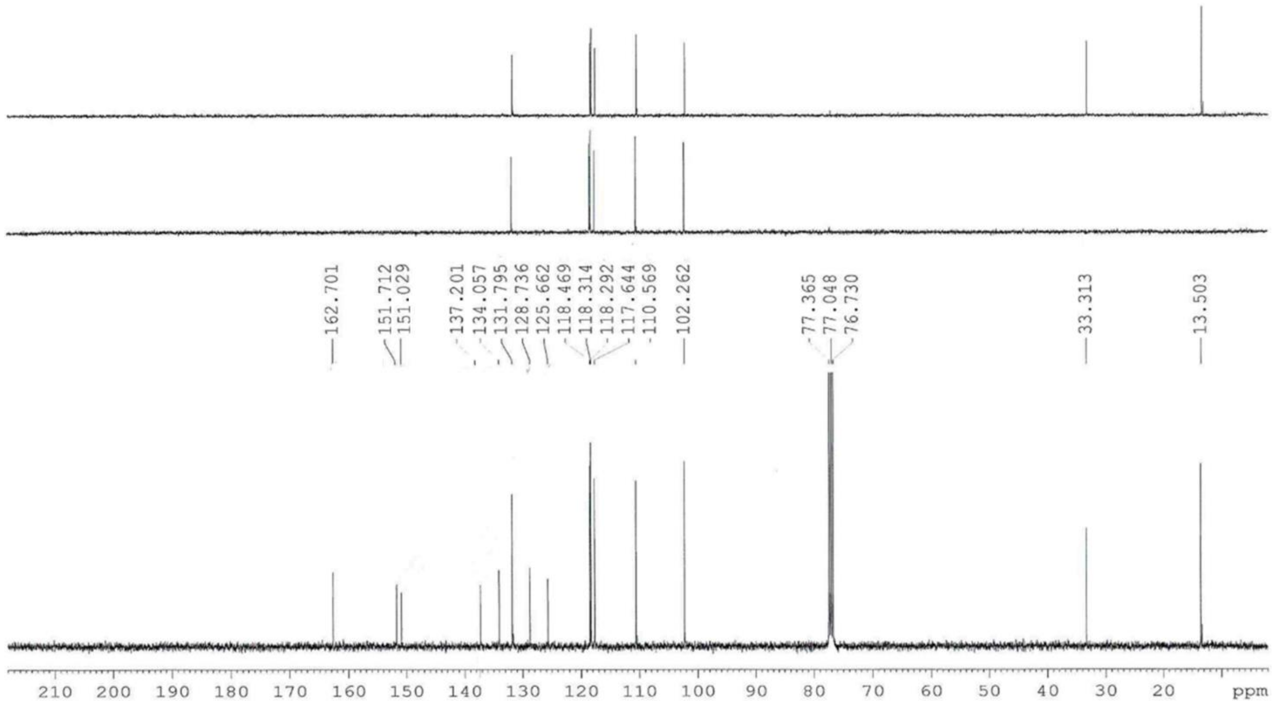

Figure S3. ^{1}H NMR ($CDCl_3$, 400 MHz) and ^{13}C ($CDCl_3$, 100 MHz) spectrum of compound **10**. DEPT-135 and DEPT-90 ^{13}C NMR spectrum were recorded to identify the signals corresponding to CH_3, CH, and C.

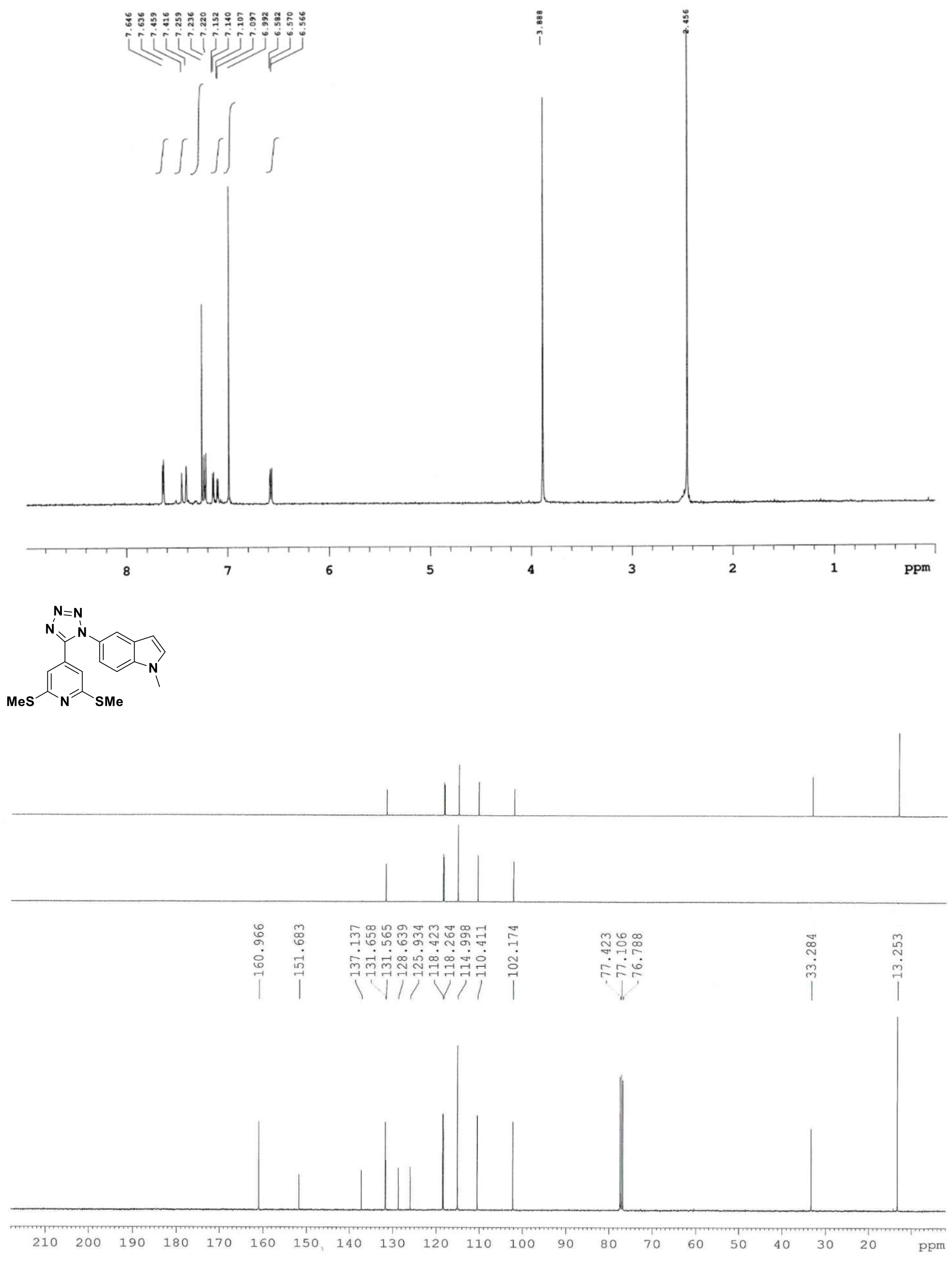

Figure S4. ^{1}H NMR ($CDCl_3$, 400 MHz) and ^{13}C ($CDCl_3$, 100 MHz) spectrum of compound **11**. DEPT-135 and DEPT-90 ^{13}C NMR spectrum were recorded to identify the signals corresponding to CH_3, CH, and C.

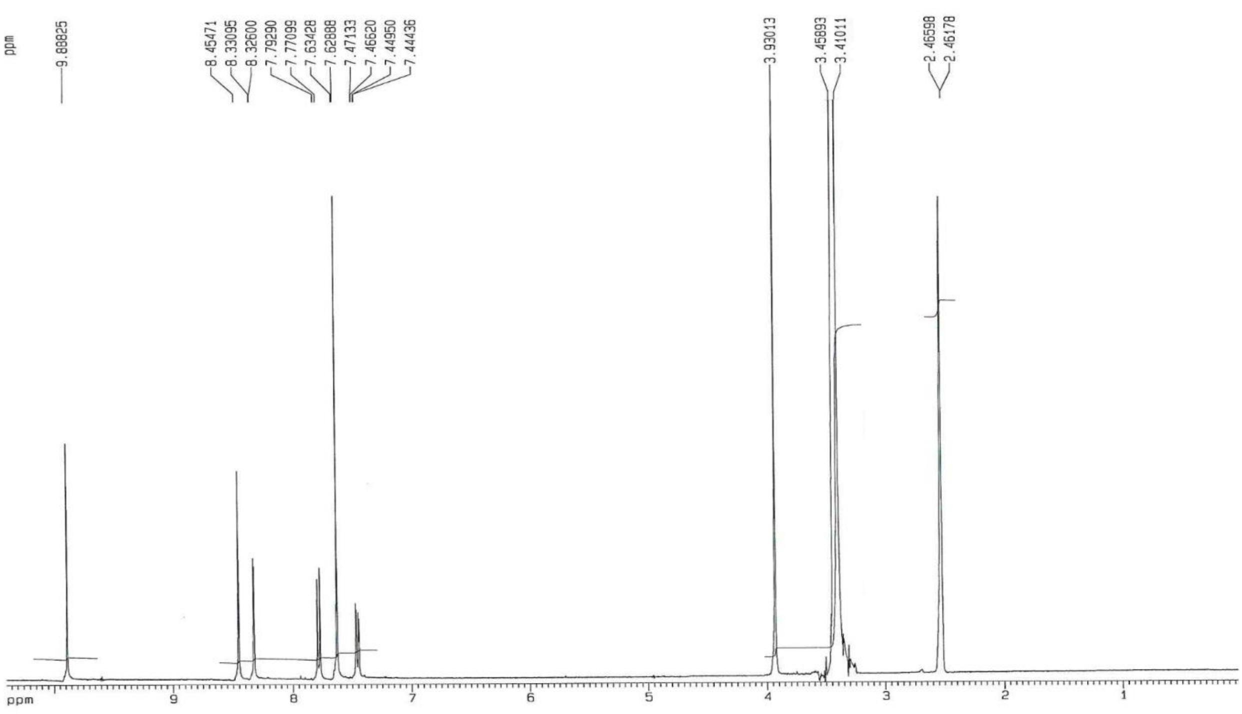

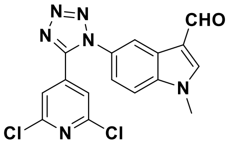

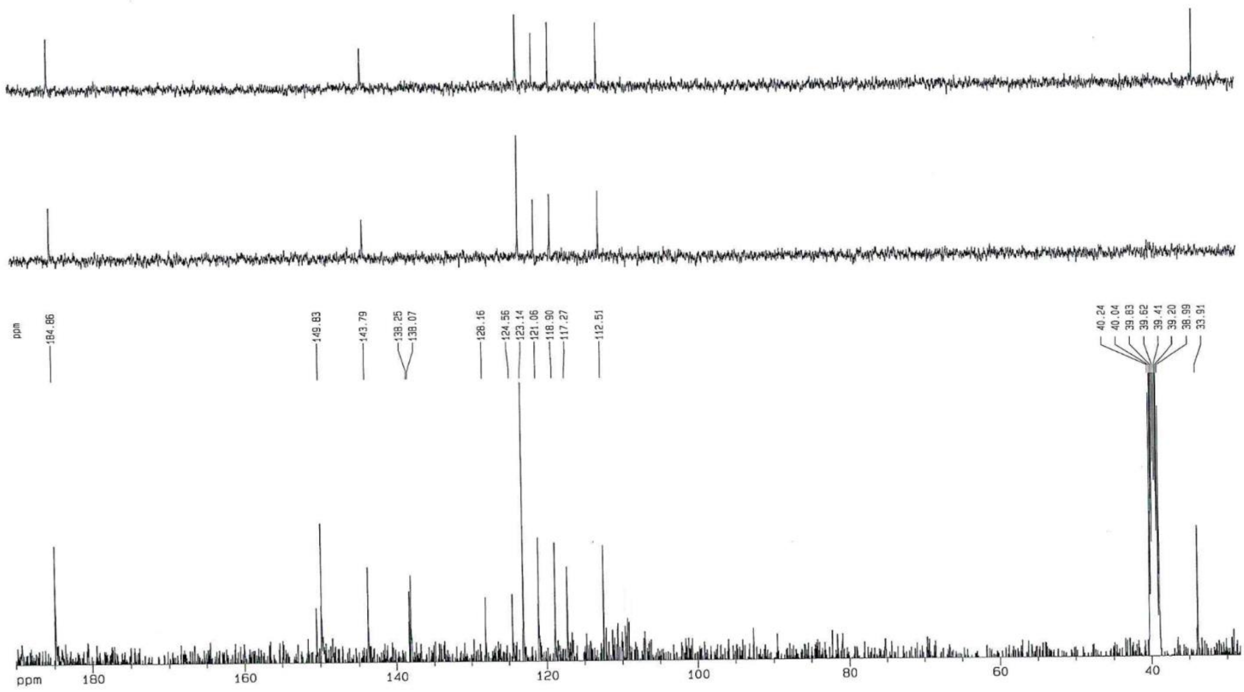

Figure S5. ^{1}H NMR (DMSO-d_6, 400 MHz) and ^{13}C (DMSO-d_6, 100 MHz) spectrum of compound **12**. DEPT-135 and DEPT-90 ^{13}C NMR spectrum were recorded to identify the signals corresponding to CH_3, CH, and C.

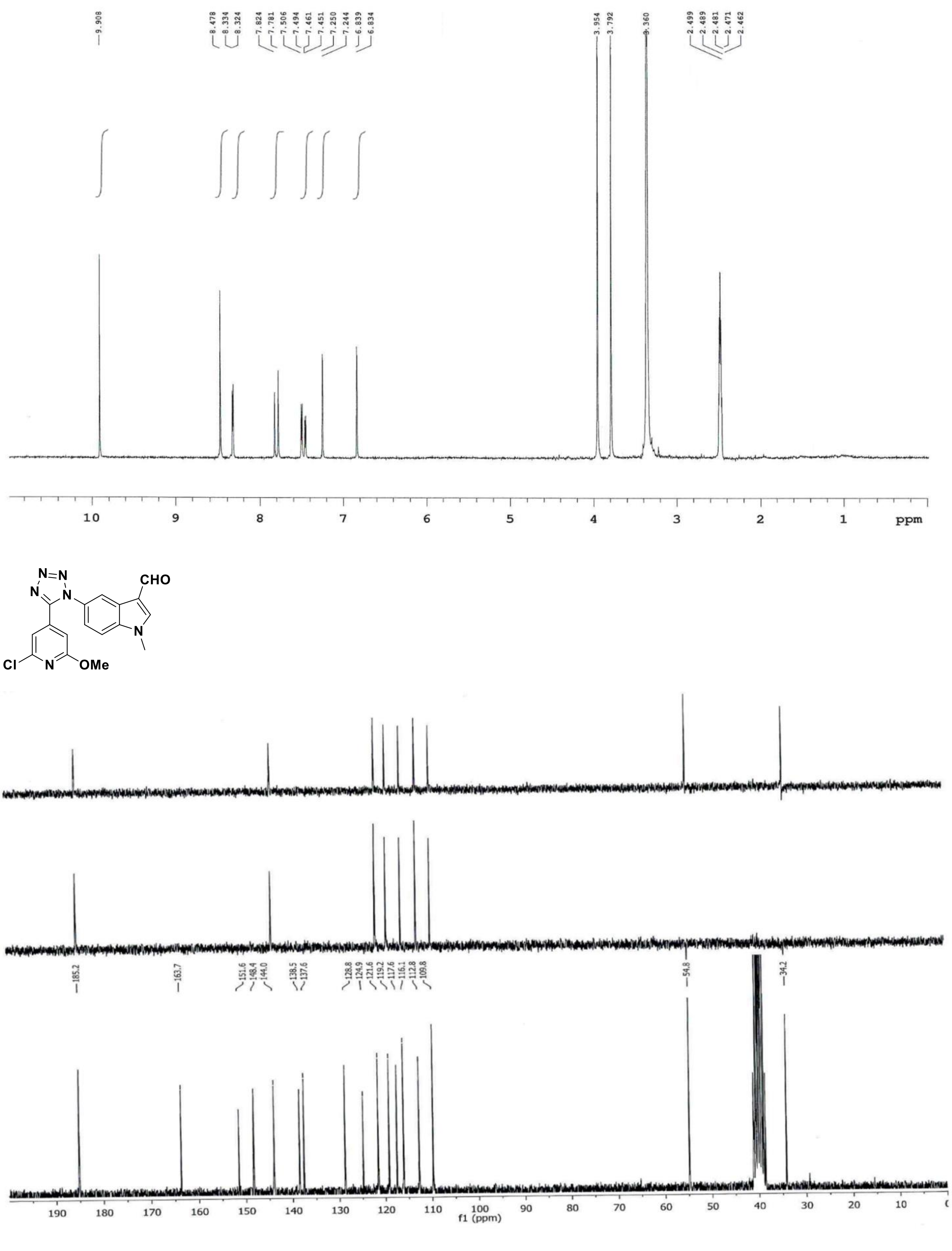

Figure S6. ^{1}H NMR (DMSO-d_6, 400 MHz) and ^{13}C (DMSO-d_6, 100 MHz) spectrum of compound **13**. DEPT-135 and DEPT-90 ^{13}C NMR spectrum were recorded to identify the signals corresponding to CH_3, CH, and C.

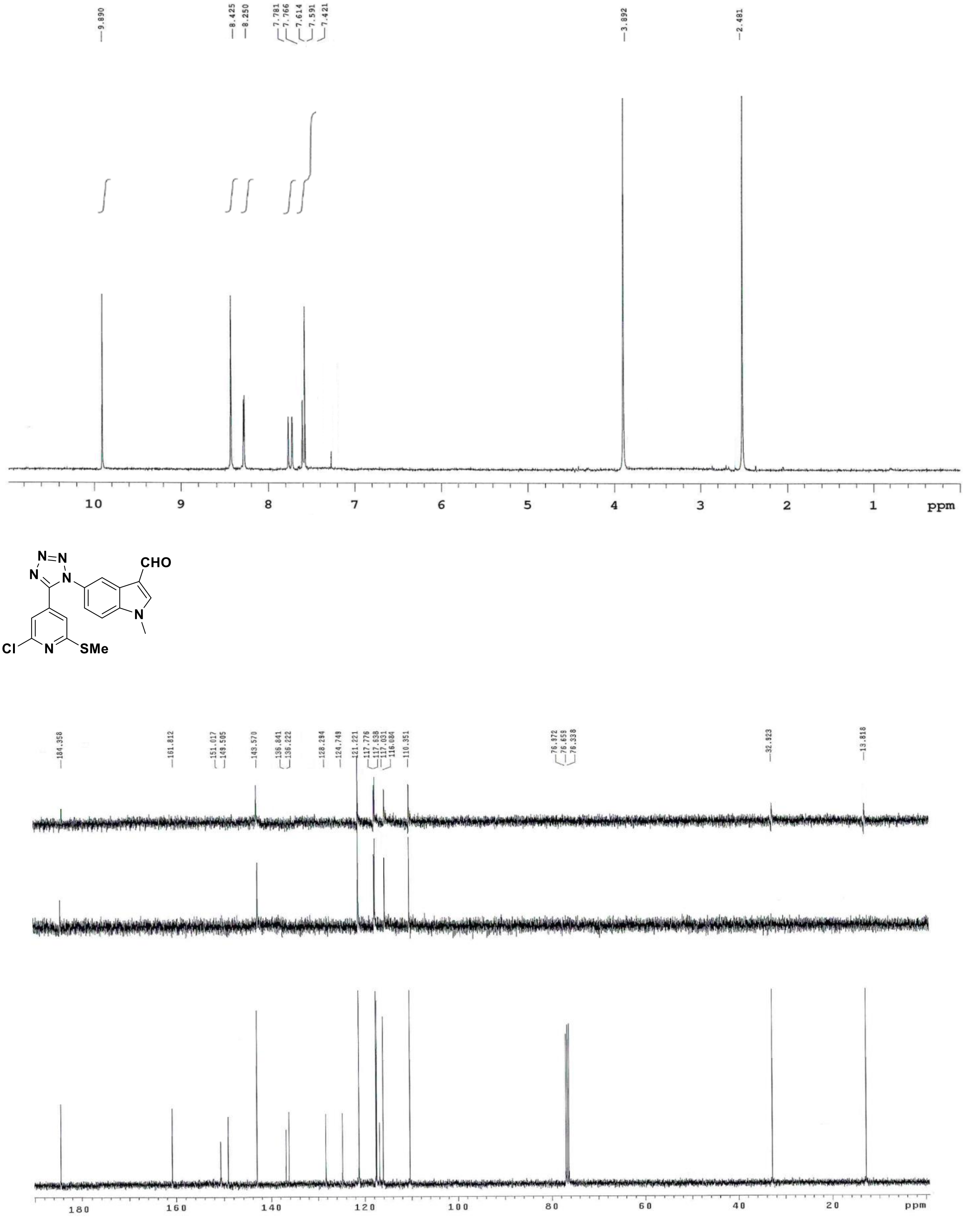

Figure S7. ^{1}H NMR ($CDCl_3$, 400 MHz) and ^{13}C ($CDCl_3$, 100 MHz) spectrum of compound **14**. DEPT-135 and DEPT-90 ^{13}C NMR spectrum were recorded to identify the signals corresponding to CH_3, CH, and C.

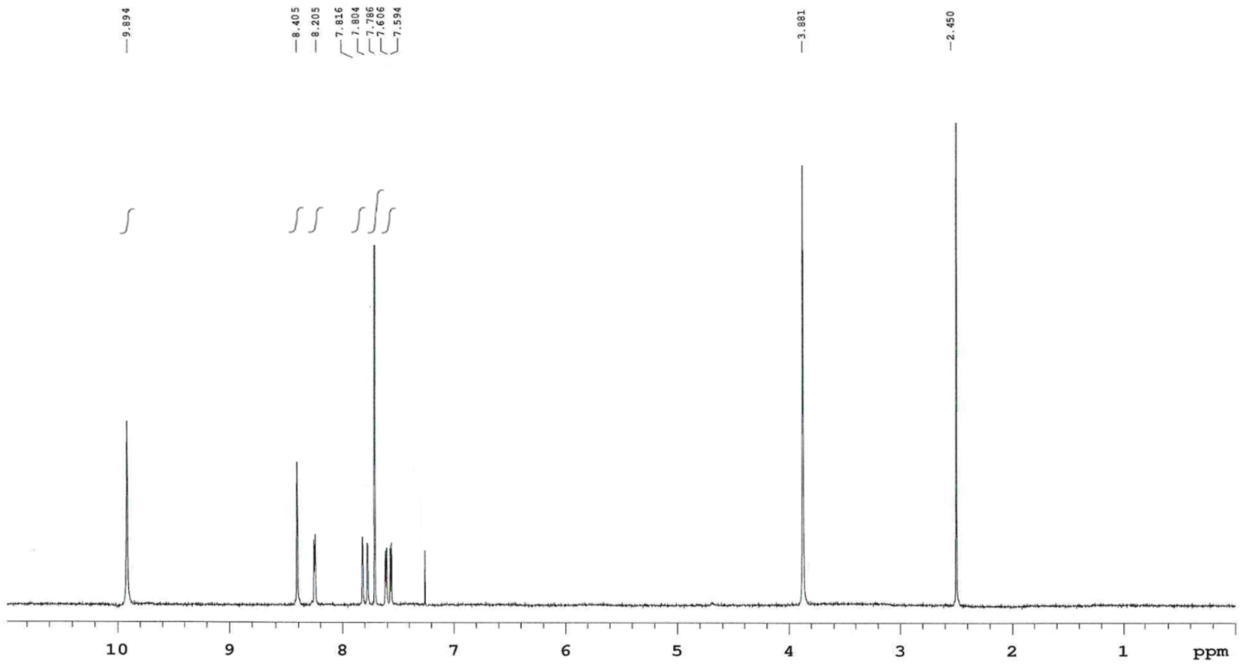

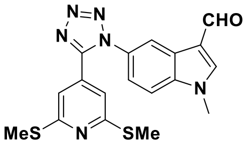

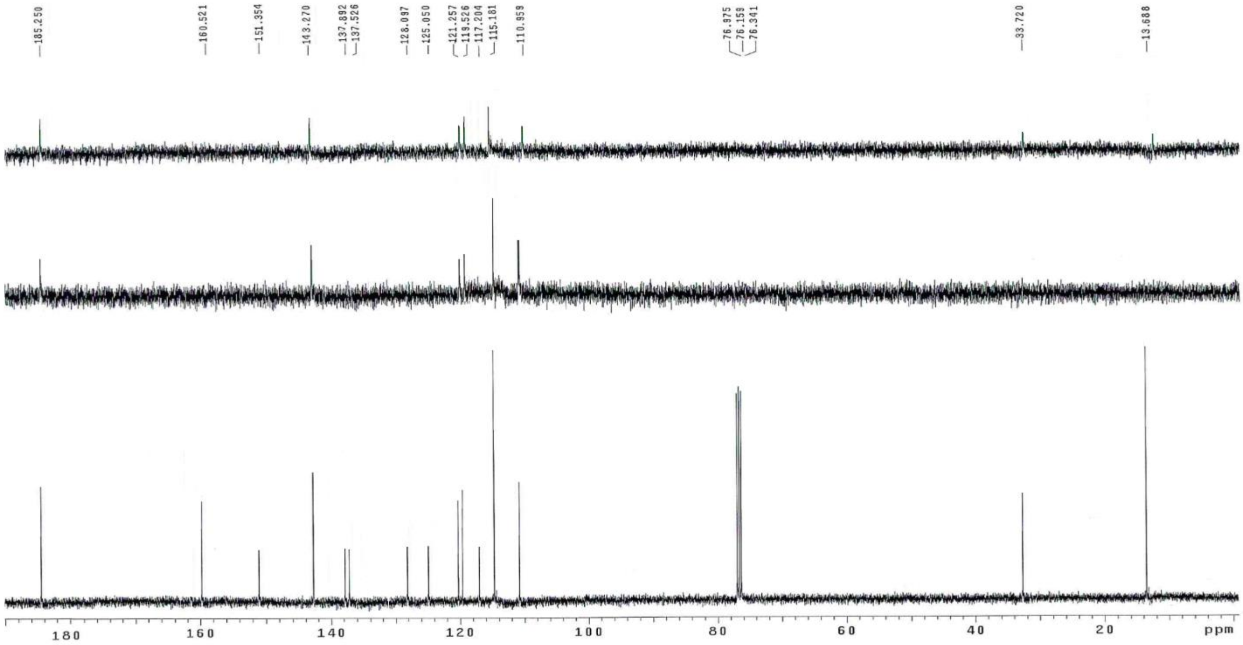

Figure S8. ^{1}H NMR ($CDCl_3$, 400 MHz) and ^{13}C ($CDCl_3$, 100 MHz) spectrum of compound **15.** DEPT-135 and DEPT-90 ^{13}C NMR spectrum were recorded to identify the signals corresponding to CH_3, CH, and C.

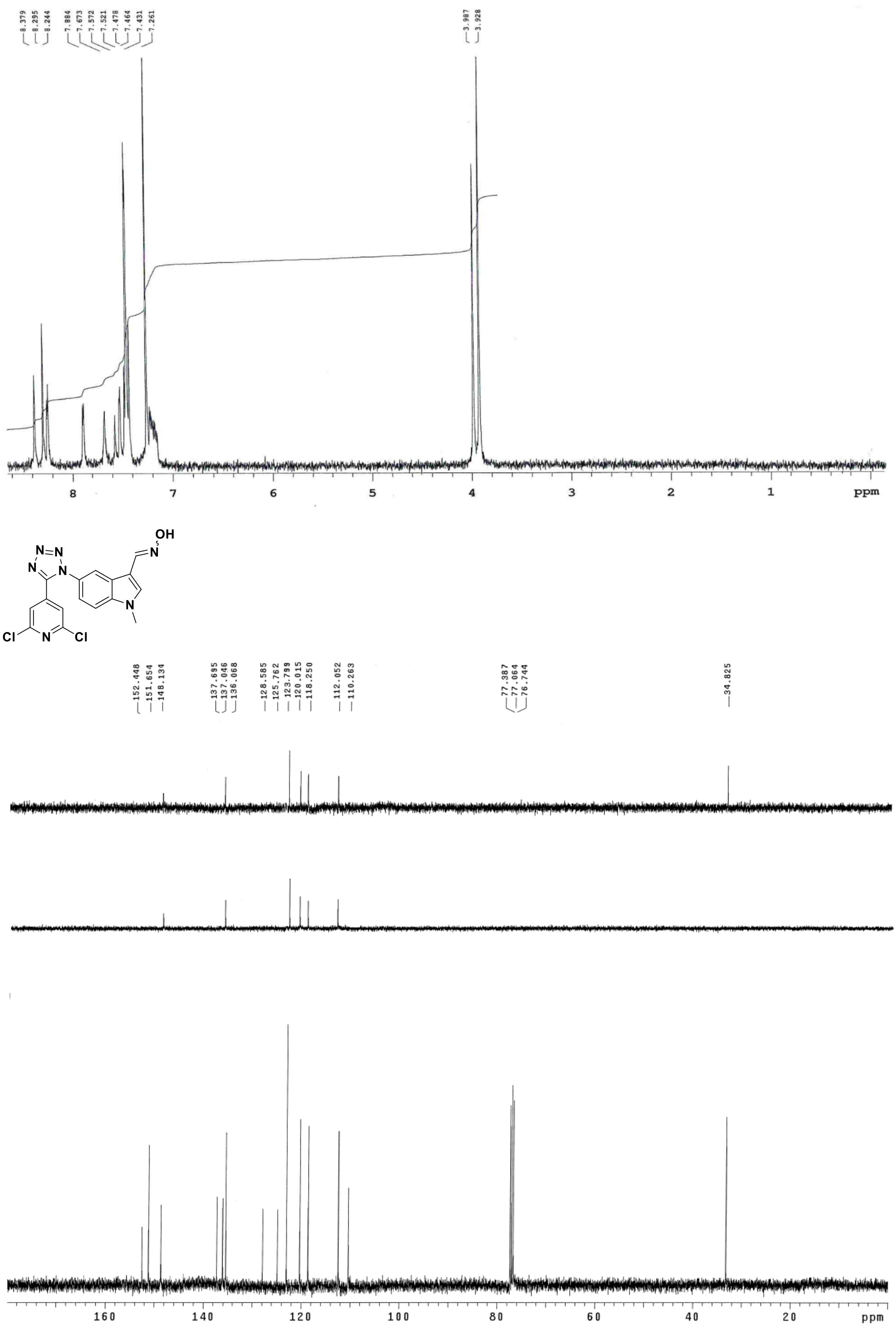

Figure S9. ^{1}H NMR ($CDCl_3$, 400 MHz) and ^{13}C ($CDCl_3$, 100 MHz) spectrum of compound **16**. DEPT-135 and DEPT-90 ^{13}C NMR spectrum were recorded to identify the signals corresponding to CH_3, CH, and C.

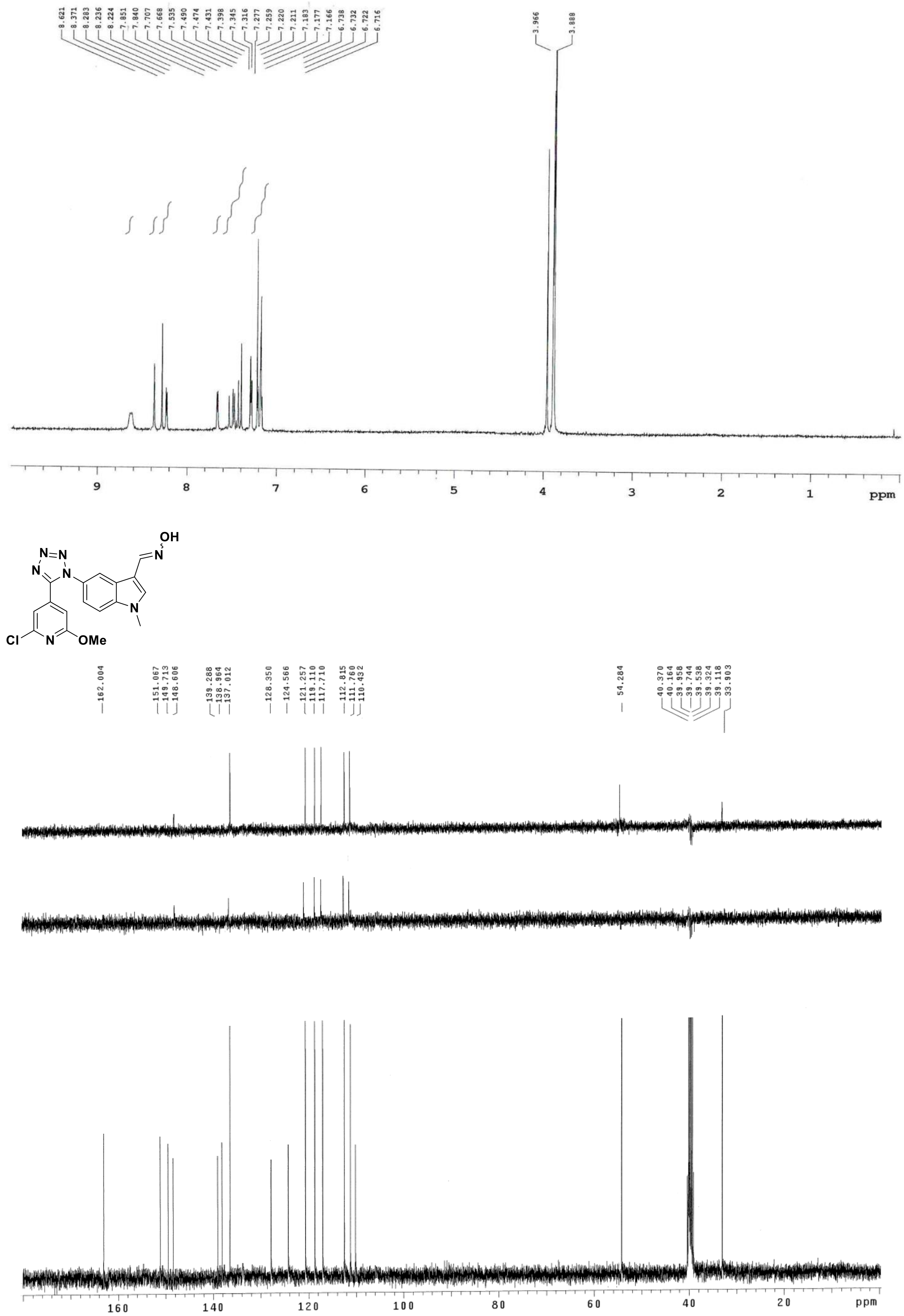

Figure S10. ^{1}H NMR ($CDCl_3$, 400 MHz) and ^{13}C (DMSO-d_6, 100 MHz) spectrum of compound **17** DEPT-135 and DEPT-90 ^{13}C NMR spectrum were recorded to identify the signals corresponding to CH_3, CH, and C.

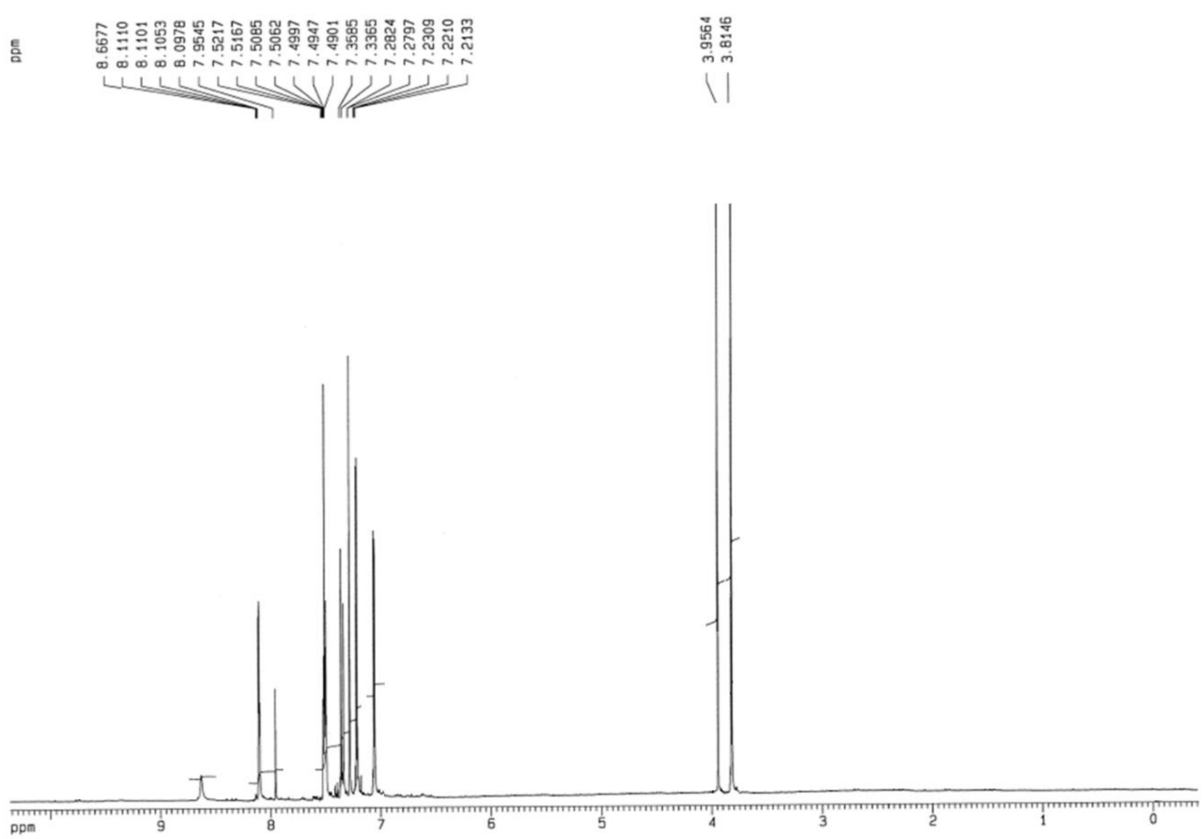

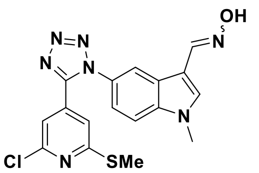

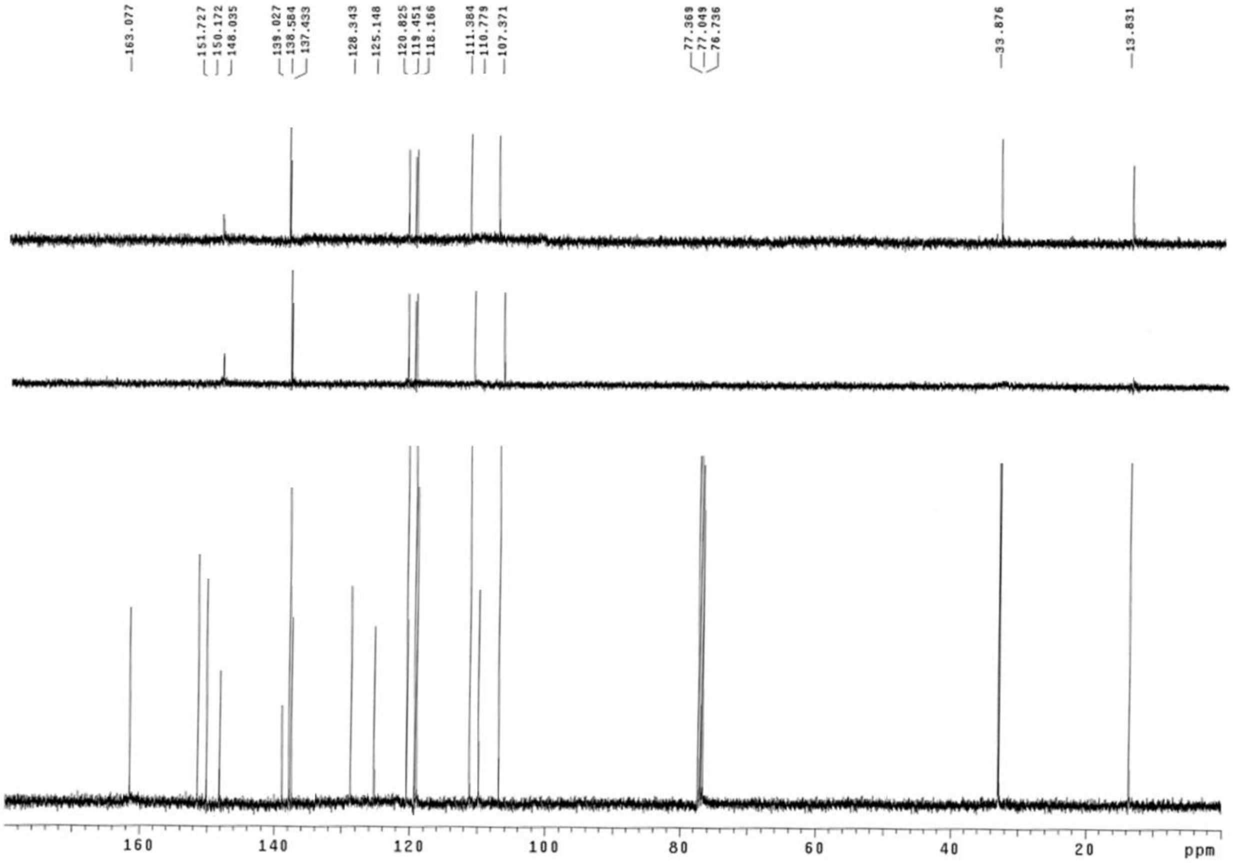

Figure S11. ^{1}H NMR ($CDCl_3$, 400 MHz) and ^{13}C ($CDCl_3$, 100 MHz) spectrum of compound **18**. DEPT-135 and DEPT-90 ^{13}C NMR spectrum were recorded to identify the signals corresponding to CH_3, CH, and C.

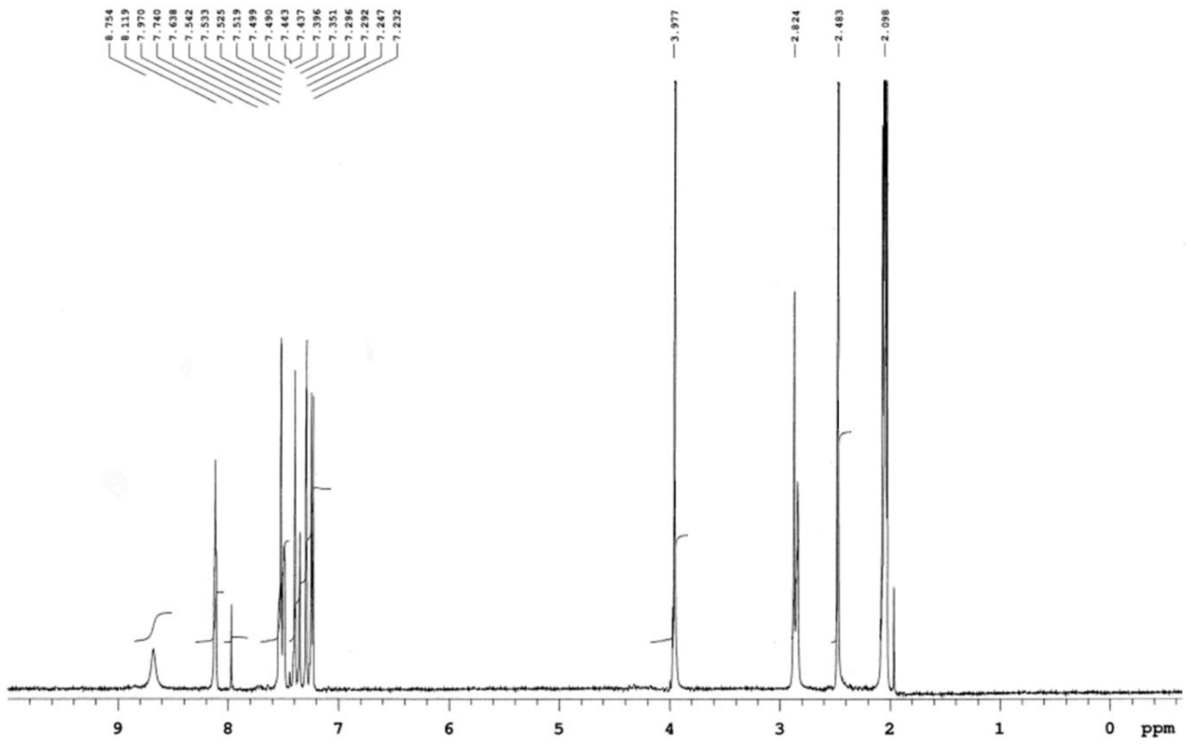

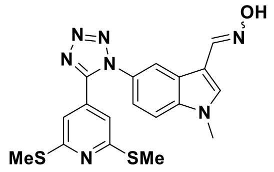

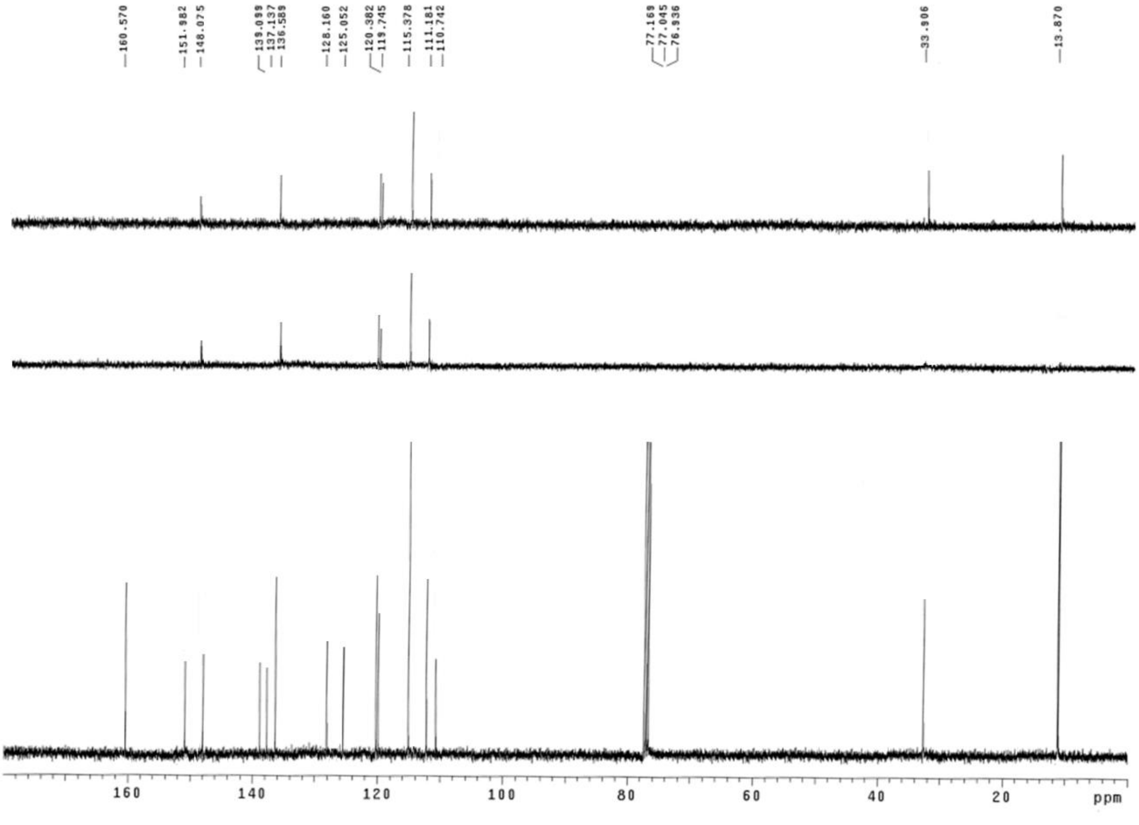

Figure S12. ^{1}H NMR (C_3D_6O, 400 MHz) and ^{13}C ($CDCl_3$,100 MHz) spectrum of compound **19.** DEPT-135 and DEPT-90 ^{13}C NMR spectrum were recorded to identify the signals corresponding to CH_3, CH, and C.

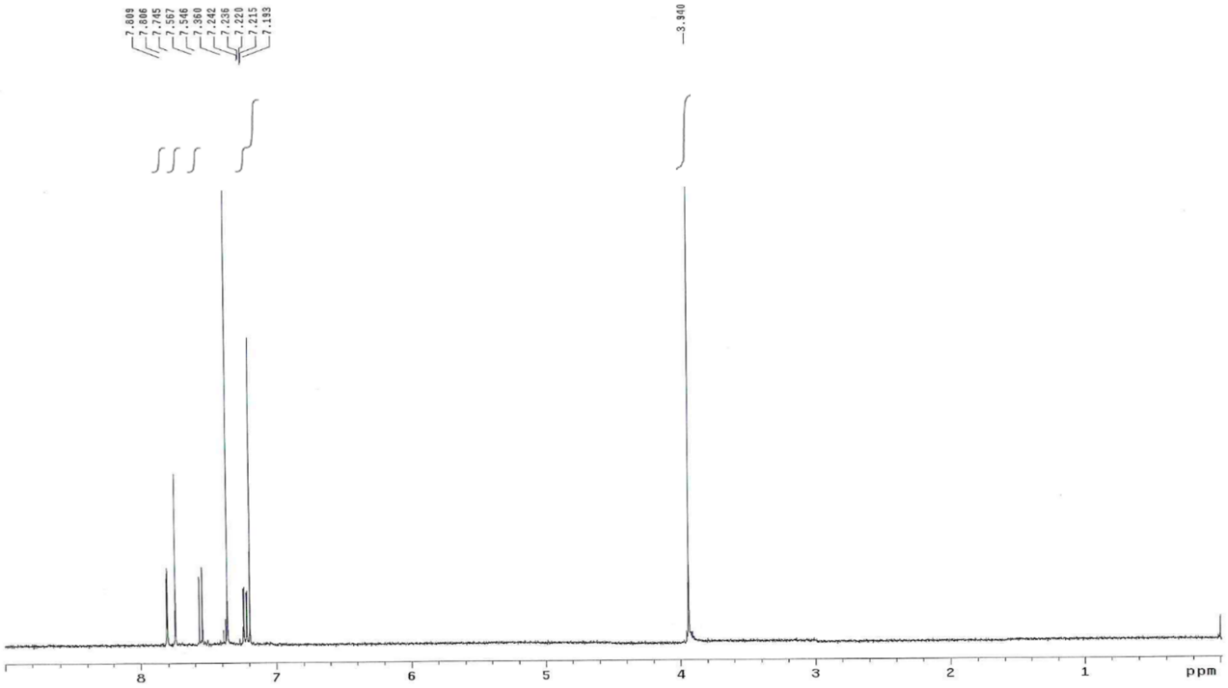

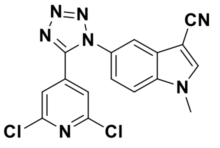

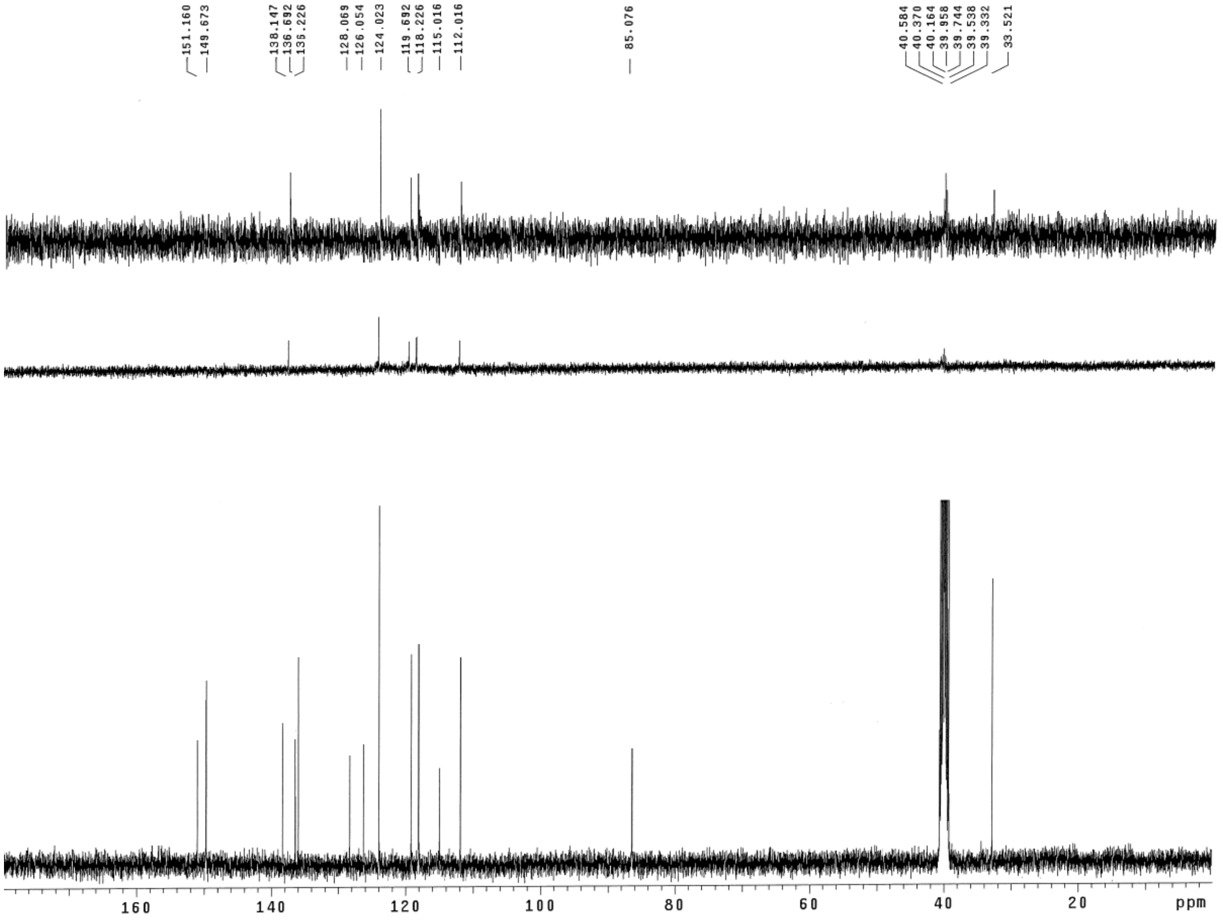

Figure S13. ^{1}H NMR ($CDCl_3$, 400 MHz) and ^{13}C (DMSO-d_6, 100 MHz) spectrum of compound **20**. DEPT-135 and DEPT-90 ^{13}C NMR spectrum were recorded to identify the signals corresponding to CH_3, CH, and C.

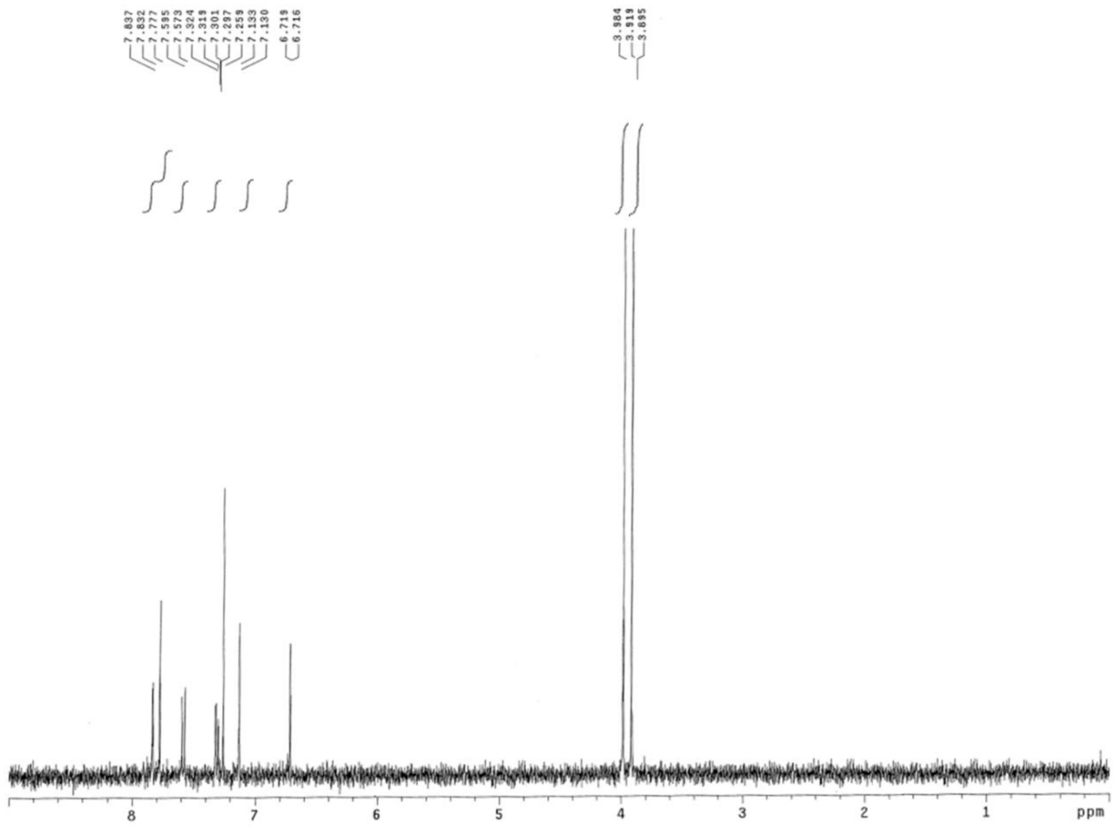

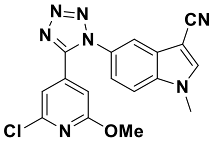

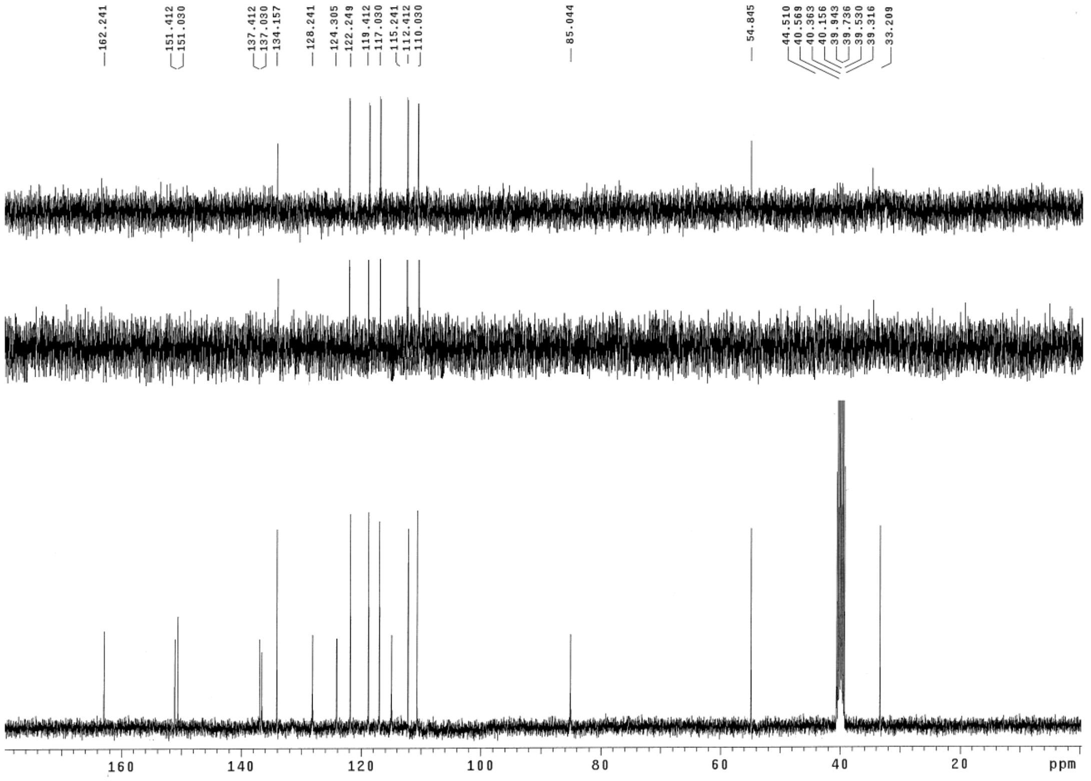

Figure S14. ^{1}H NMR ($CDCl_3$, 400 MHz) and ^{13}C (DMSO-d_6, 100 MHz) spectrum of compound **21** DEPT-135 and DEPT-90 ^{13}C NMR spectrum were recorded to identify the signals corresponding to CH_3, CH, and C.

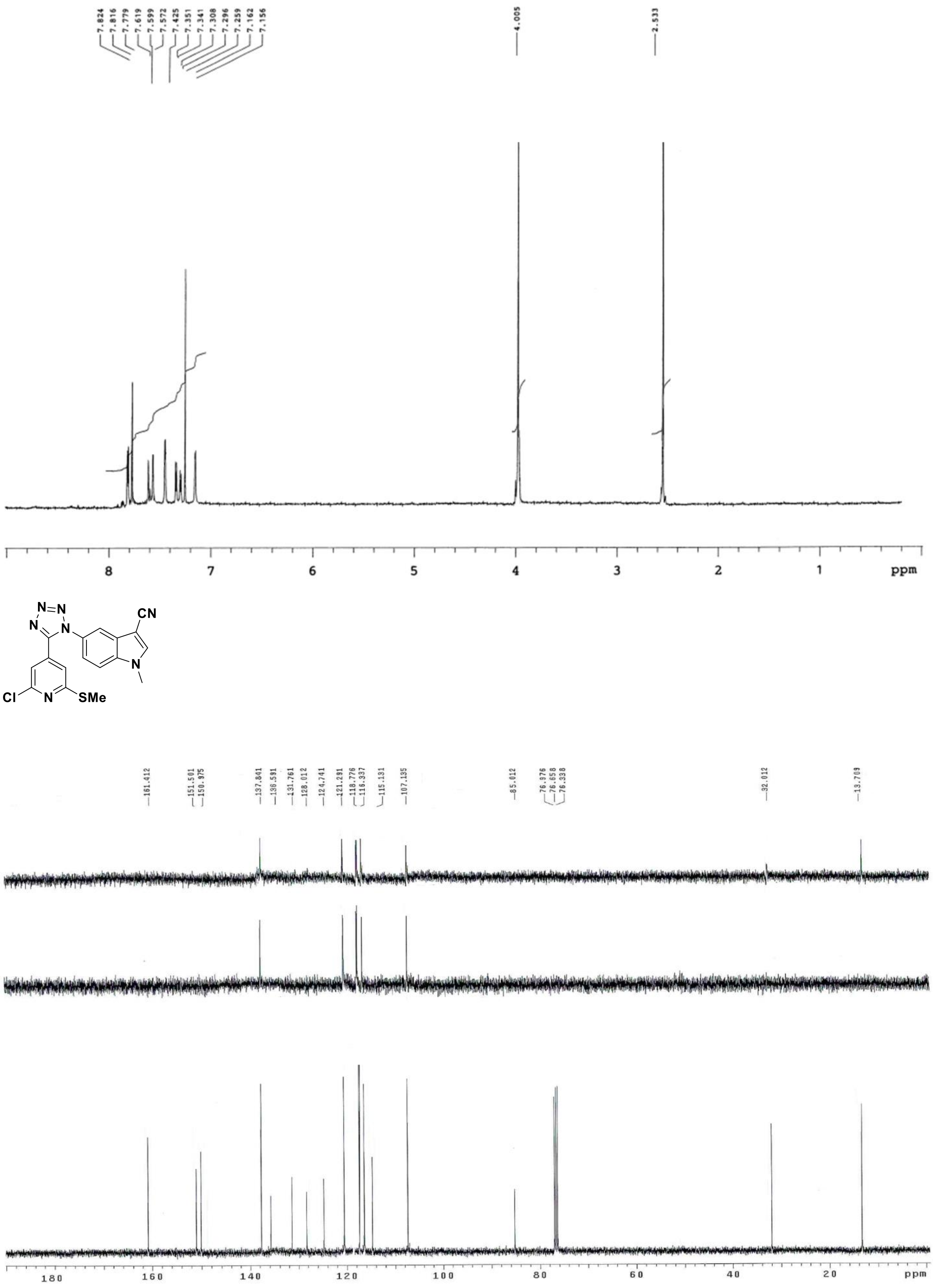

Figure S15. ^{1}H NMR ($CDCl_3$, 400 MHz) and ^{13}C ($CDCl_3$, 100 MHz) spectrum of compound **22**. DEPT-135 and DEPT-90 ^{13}C NMR spectrum were recorded to identify the signals corresponding to CH_3, CH, and C.

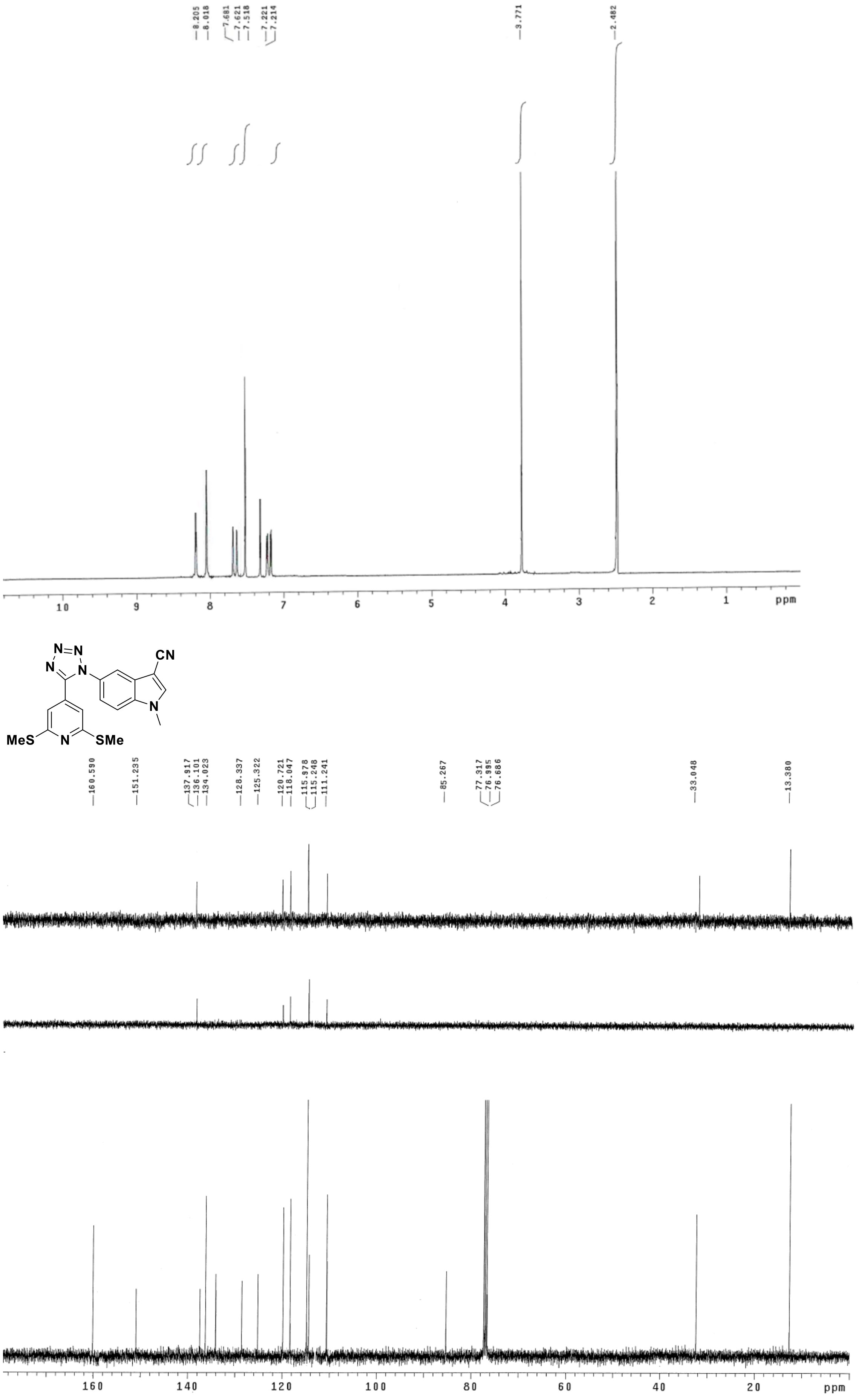

Figure S16. ^{1}H NMR ($CDCl_3$, 400 MHz) and ^{13}C ($CDCl_3$, 100 MHz) spectrum of compound **23.** DEPT-135 and DEPT-90 ^{13}C NMR spectrum were recorded to identify the signals corresponding to CH_3, CH, and C.

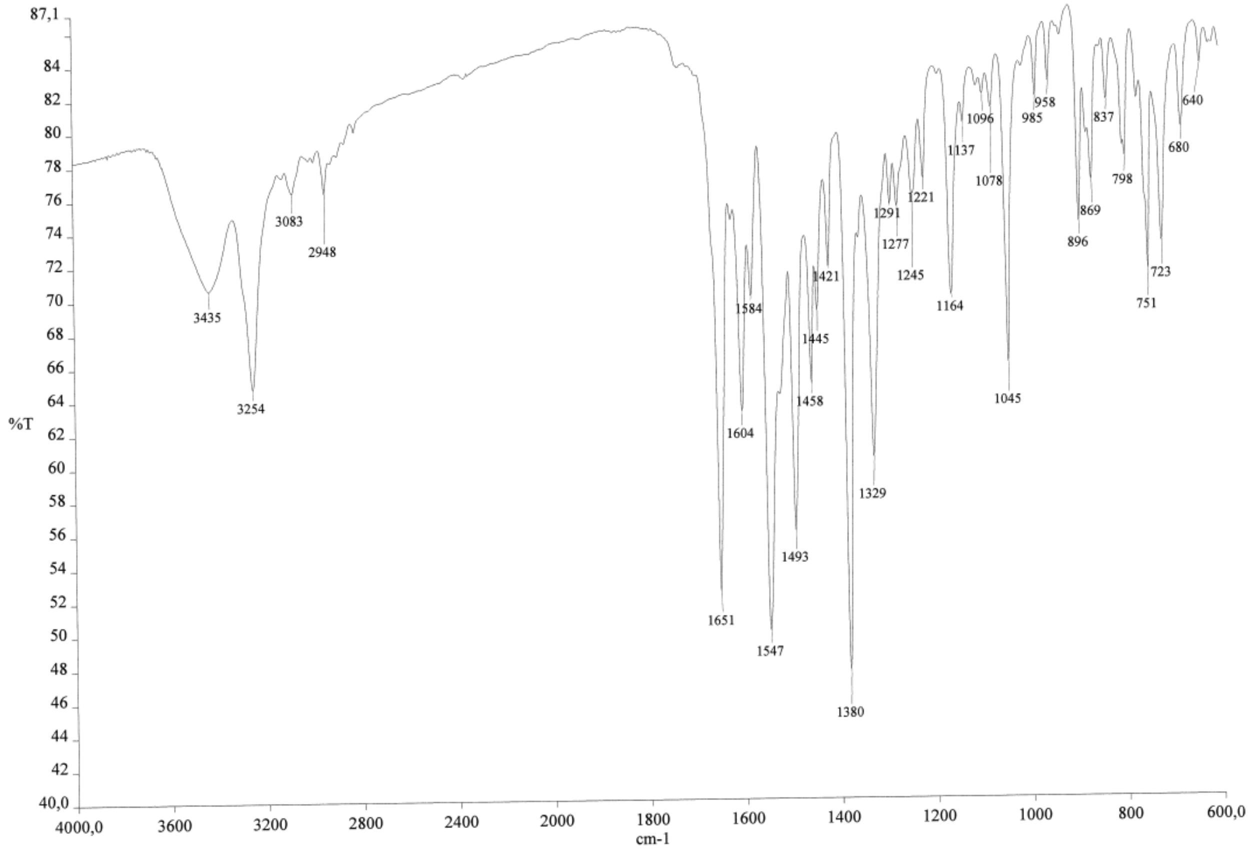

Figure S17. IR spectrum of compound **7.**

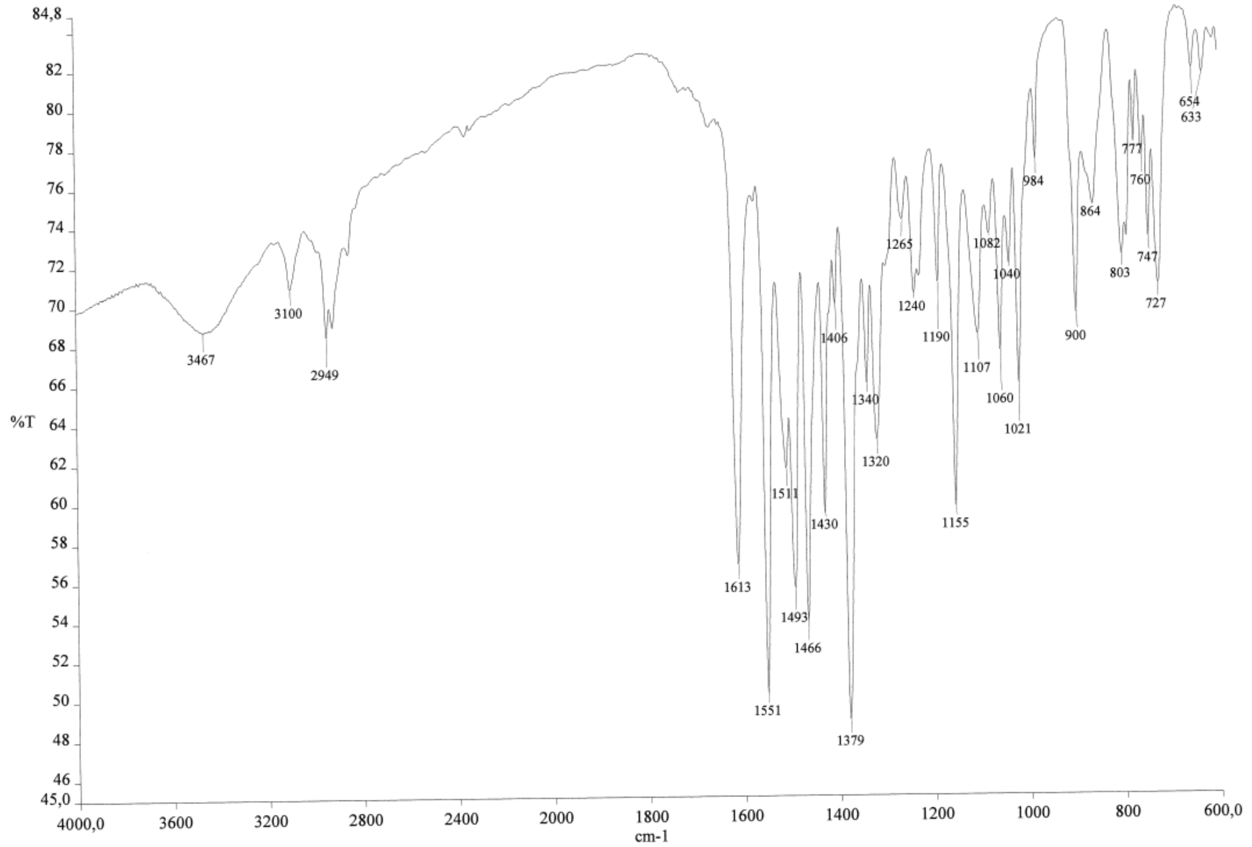

Figure S18. IR spectrum of compound **9.**

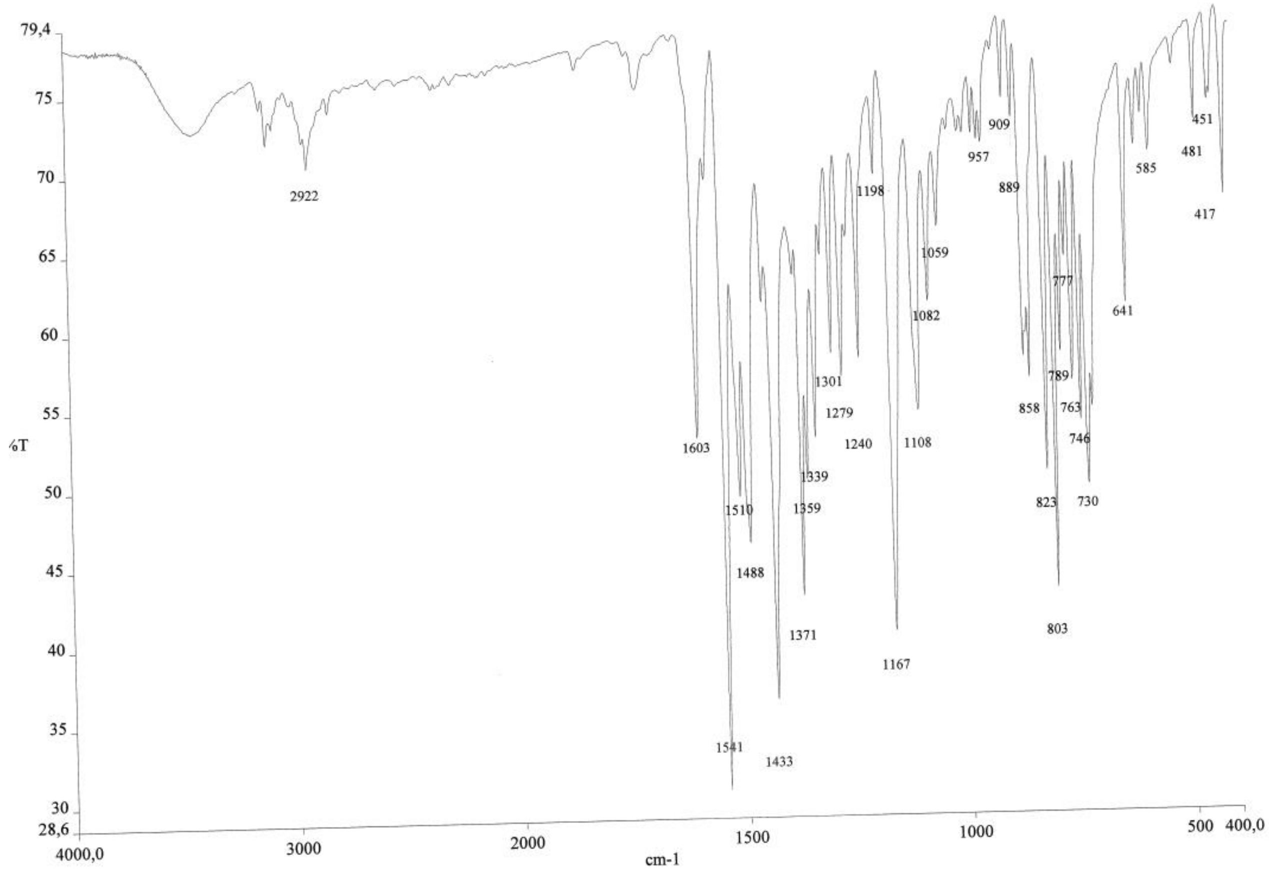

Figure S19. IR spectrum of compound **10.**

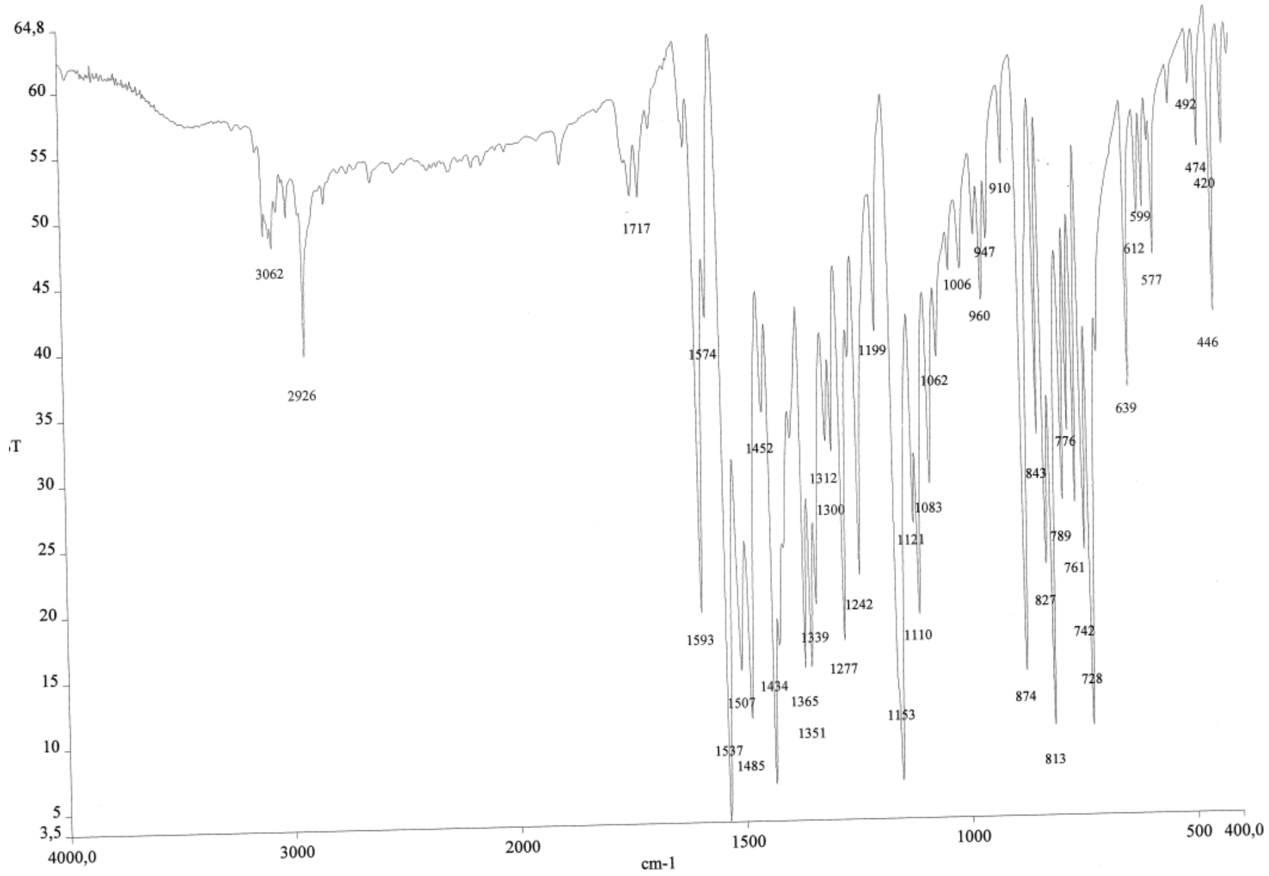

Figure S20. IR spectrum of compound **11.**

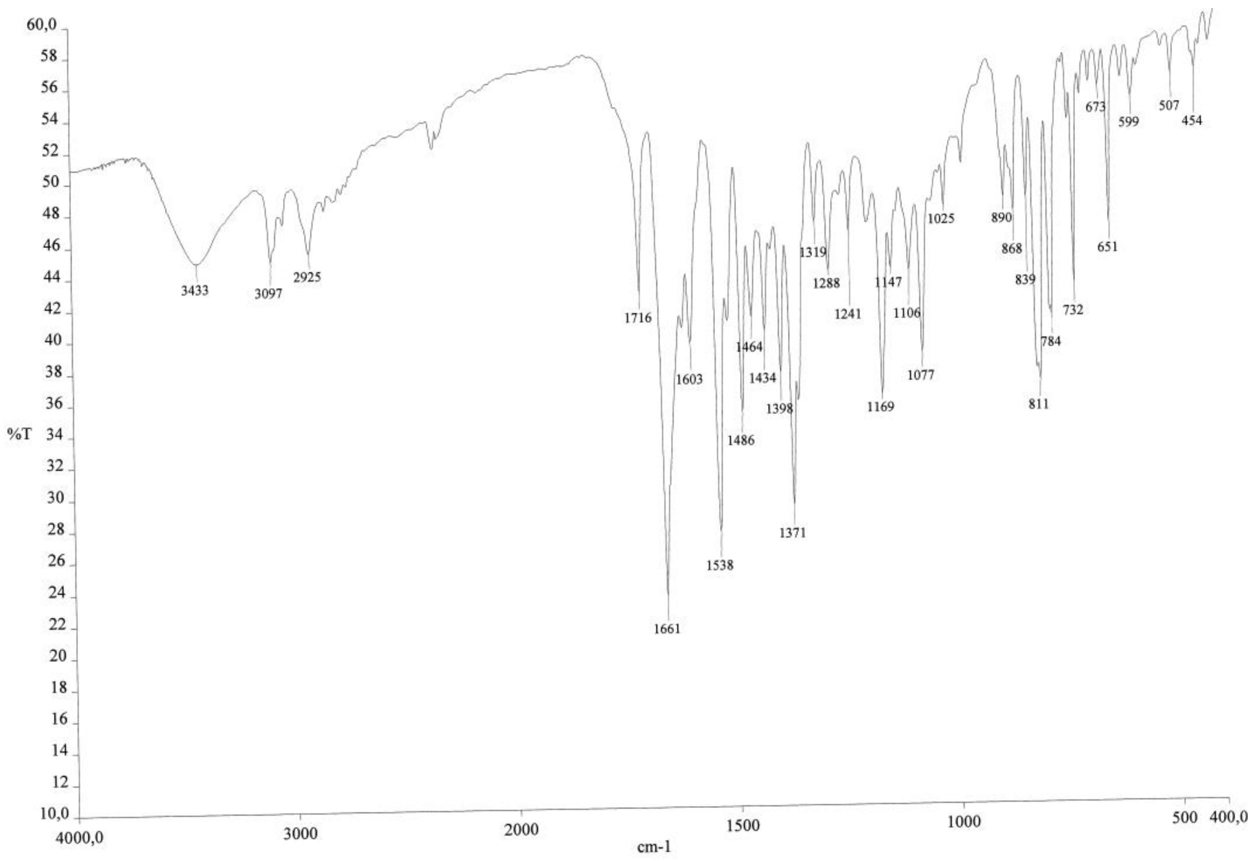

Figure S21. IR spectrum of compound **12.**

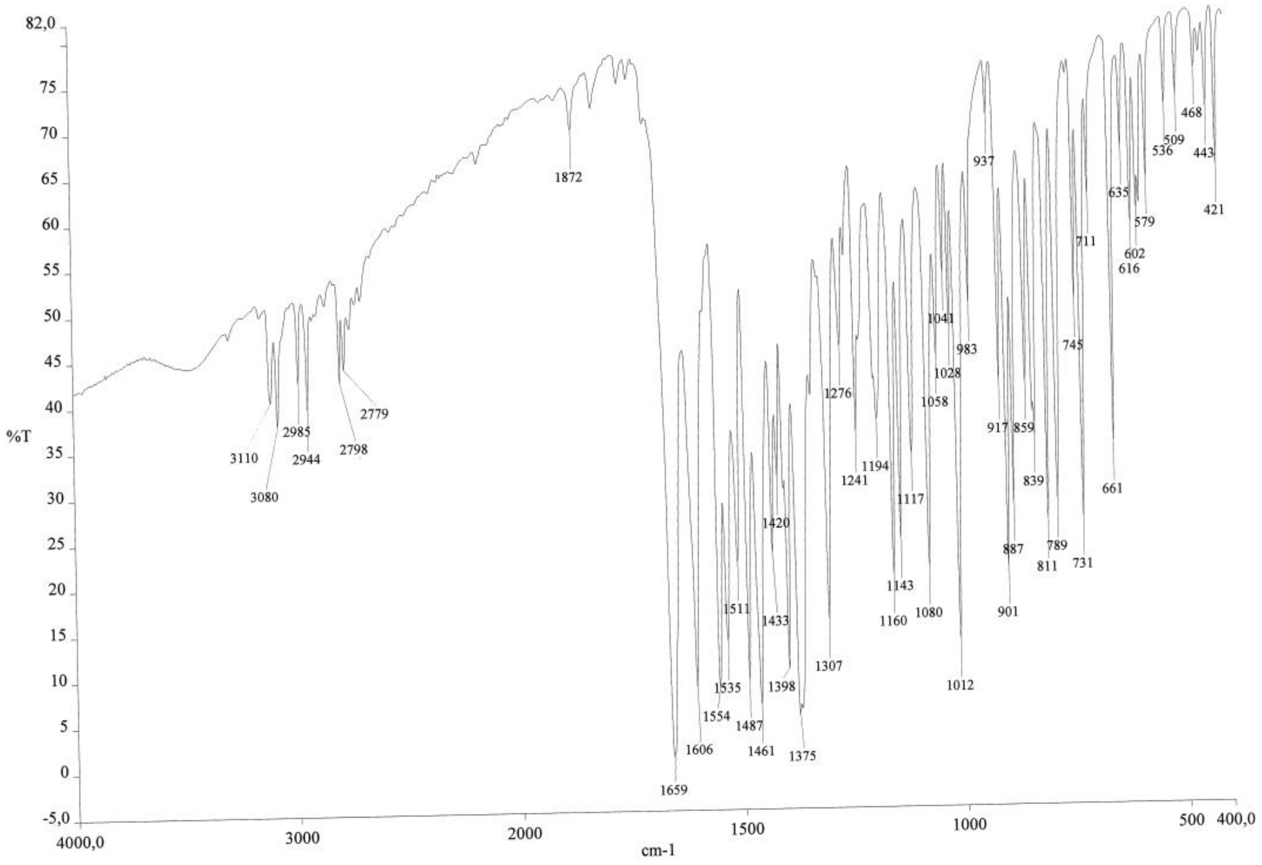

Figure S22. IR spectrum of compound **13.**

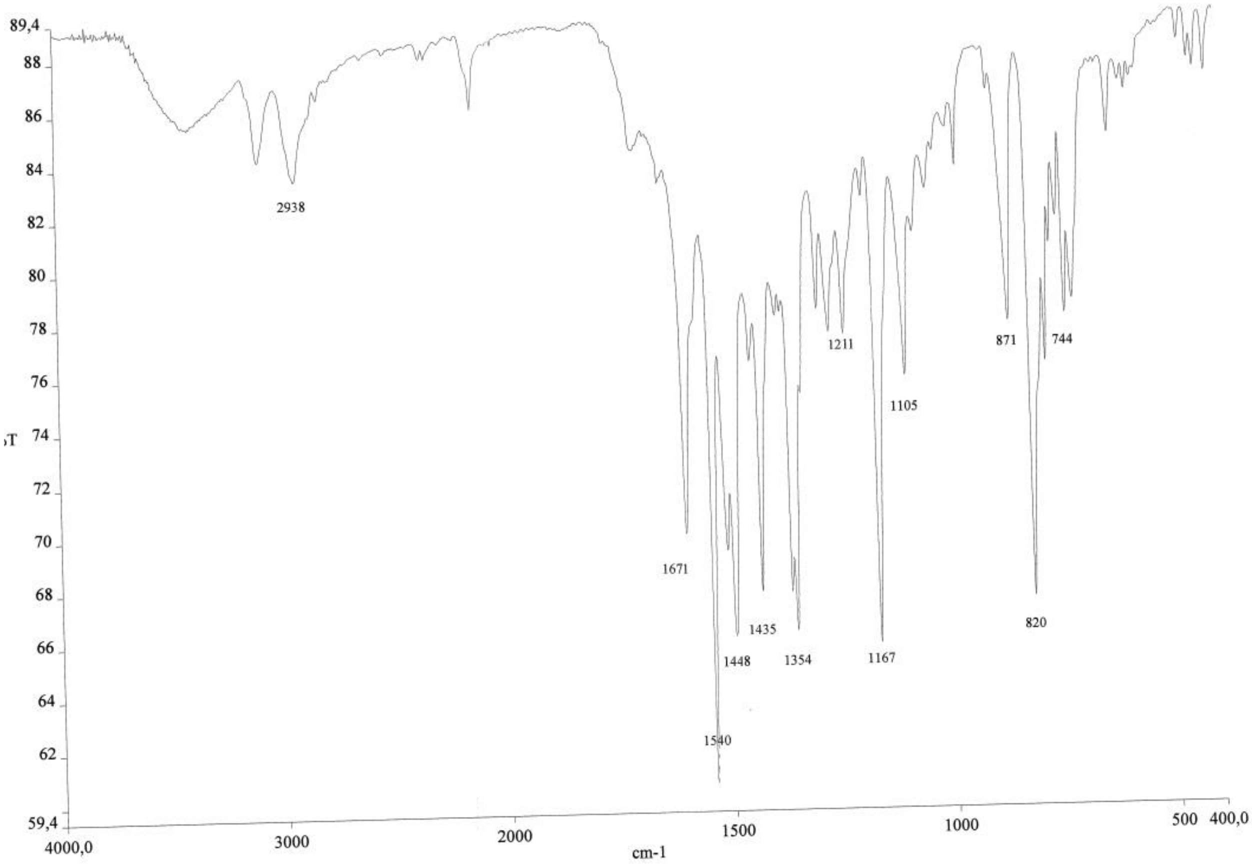

Figure S23. IR spectrum of compound **14.**

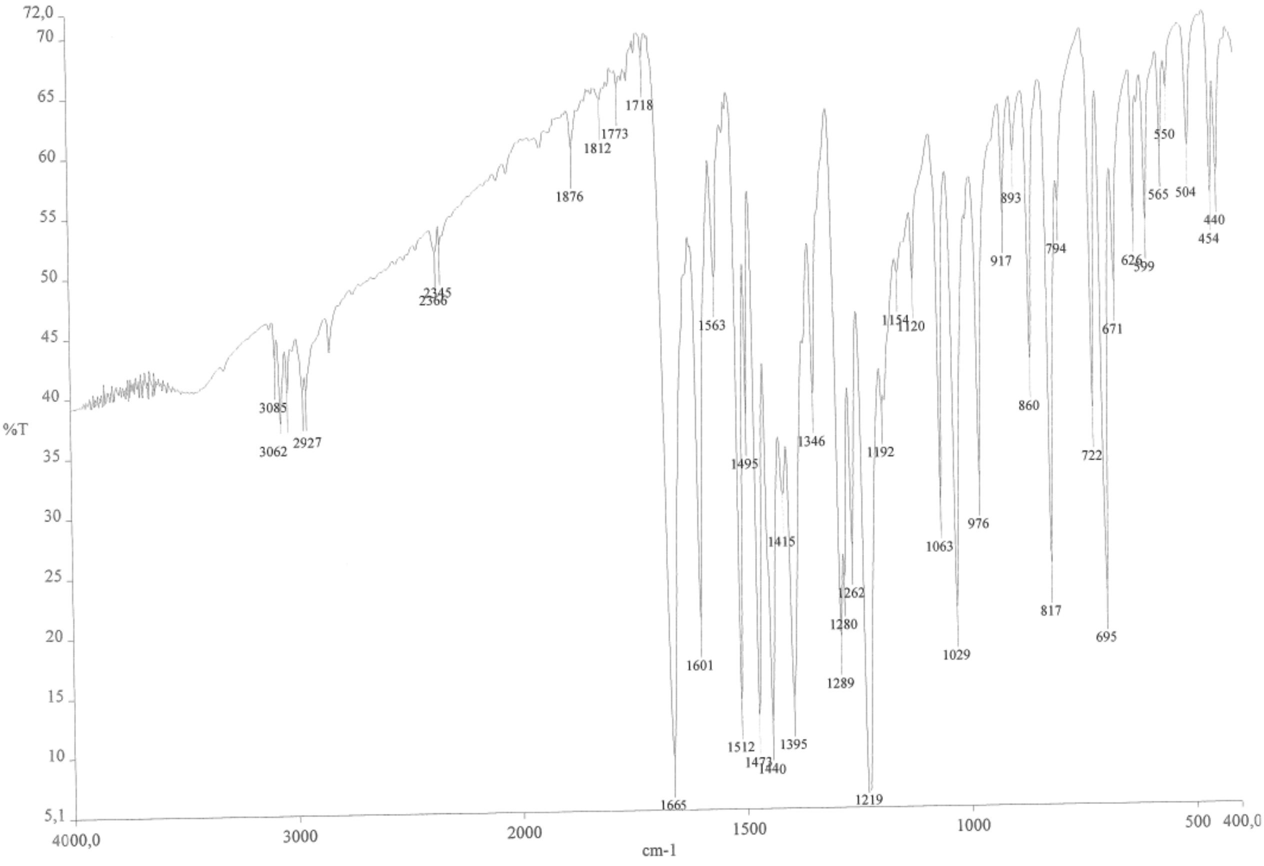

Figure S24. IR spectrum of compound **15.**

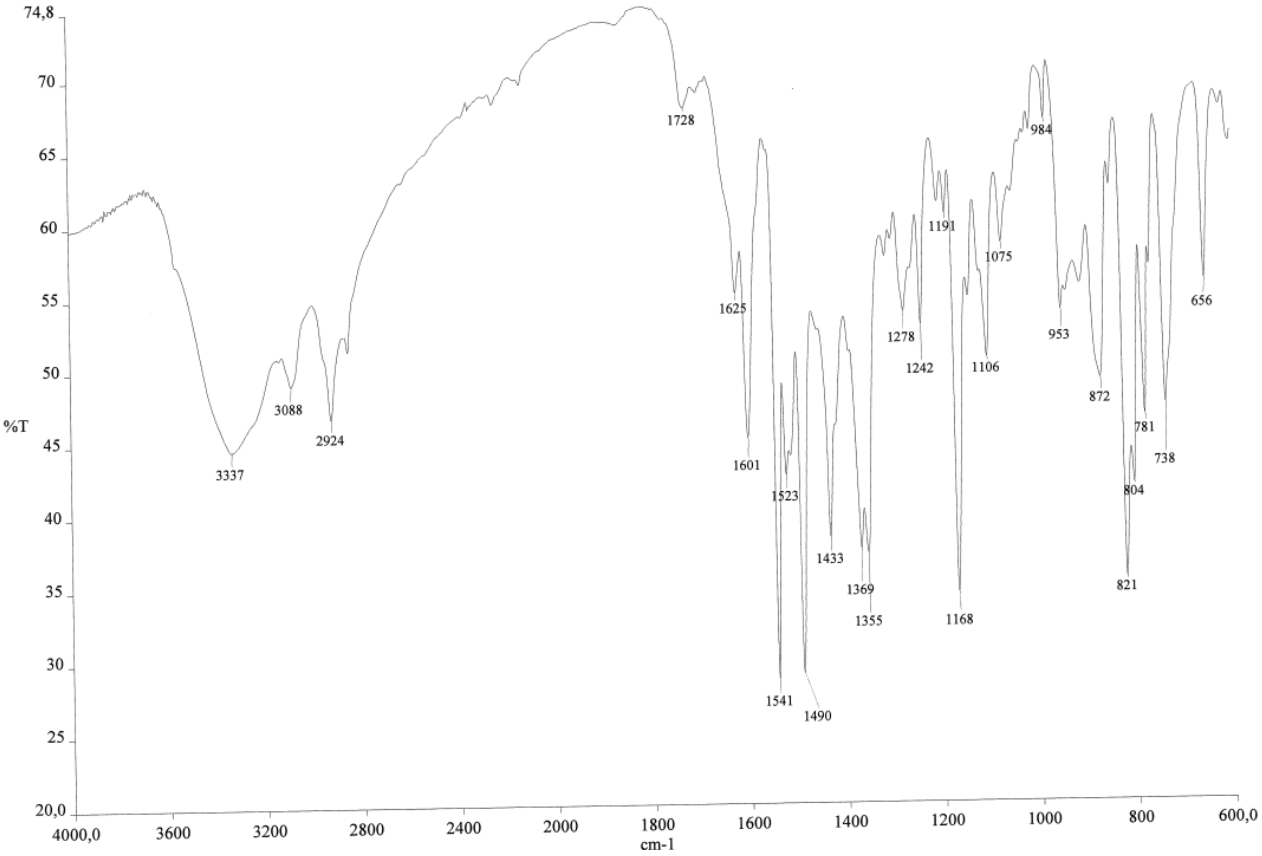

Figure S25. IR spectrum of compound **16.**

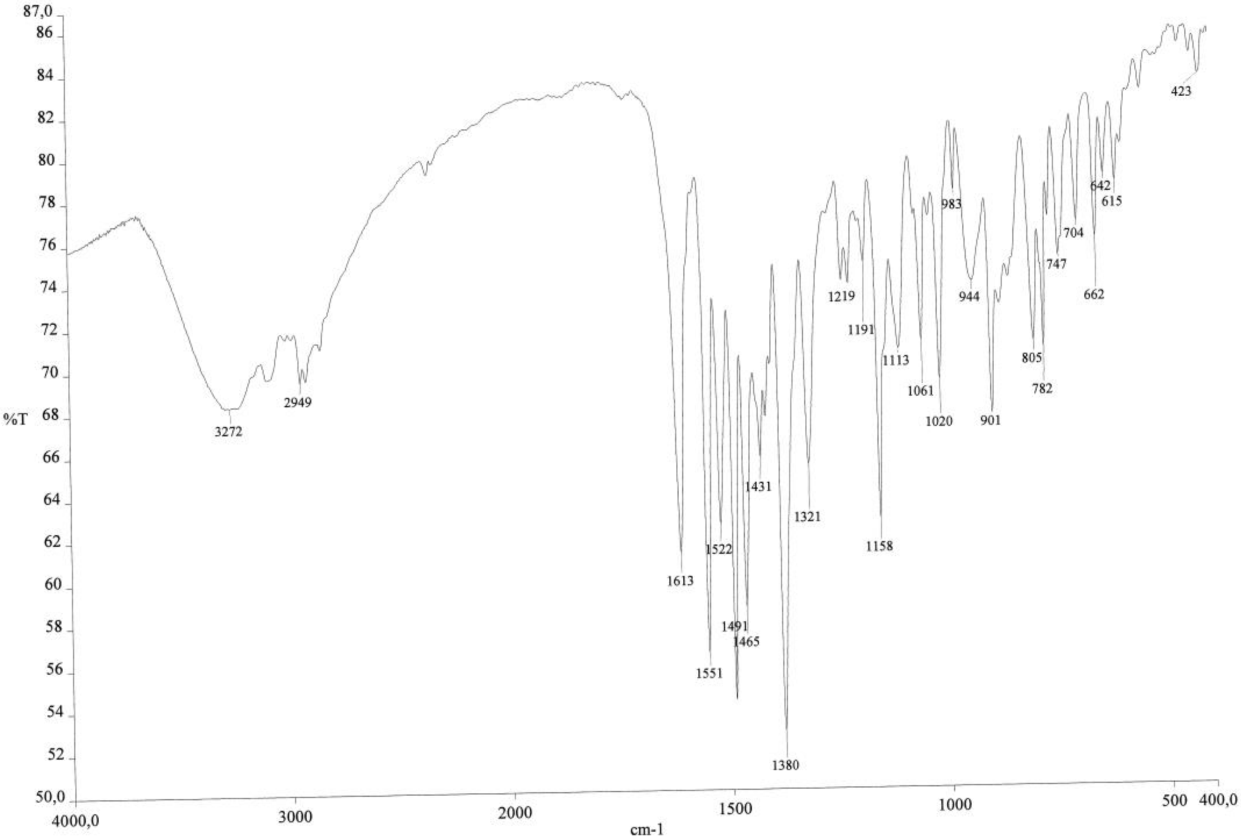

Figure S26. IR spectrum of compound **17.**

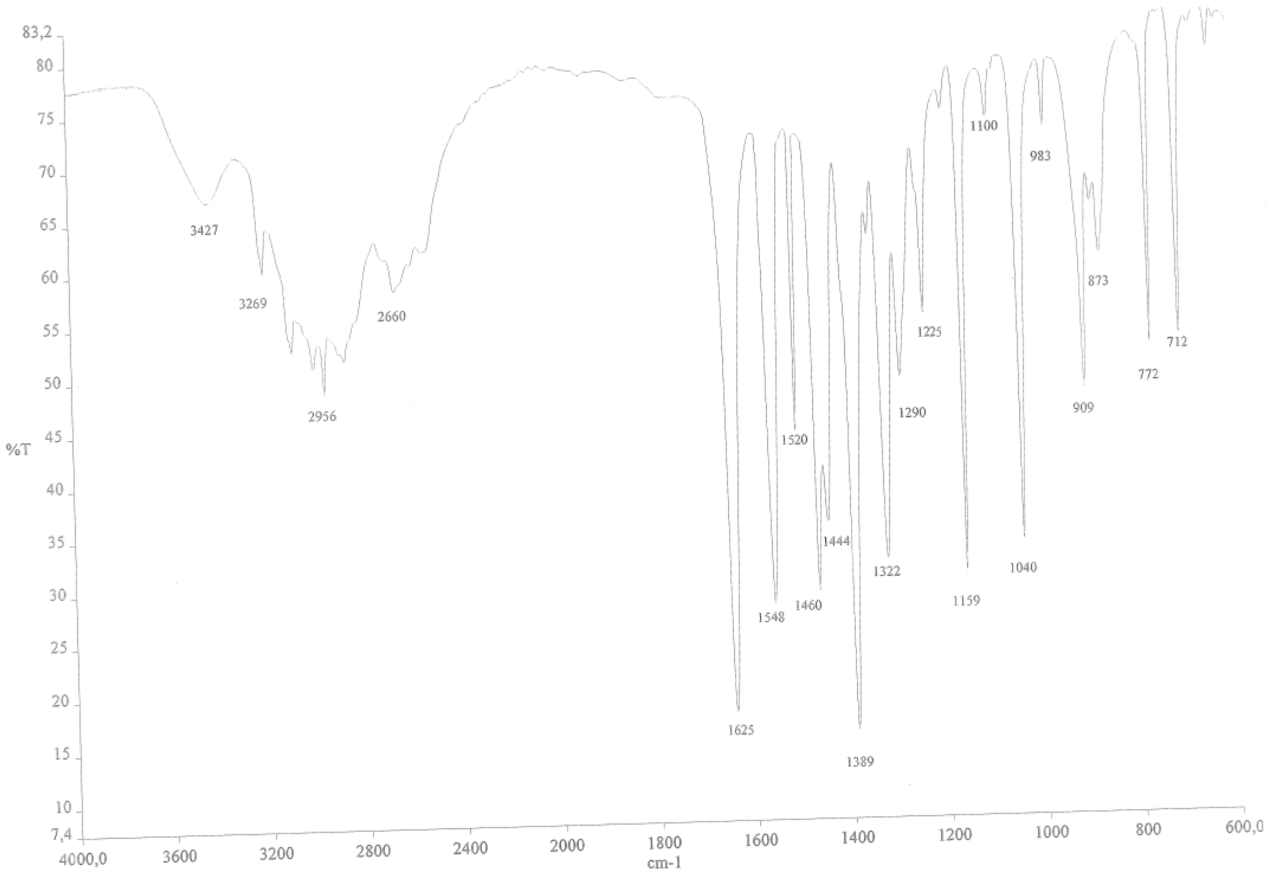

Figure S27. IR spectrum of compound **18.**

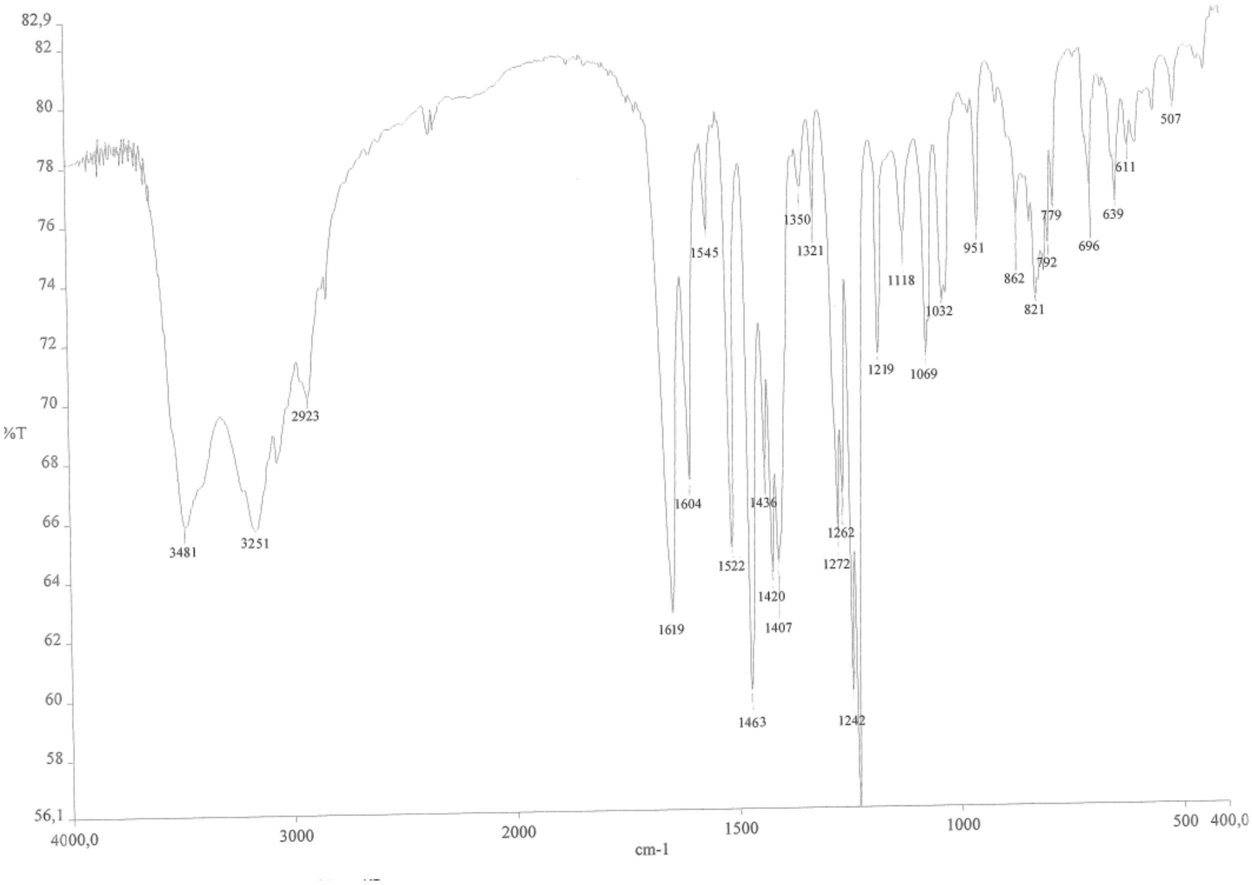

Figure S28. IR spectrum of compound **19.**

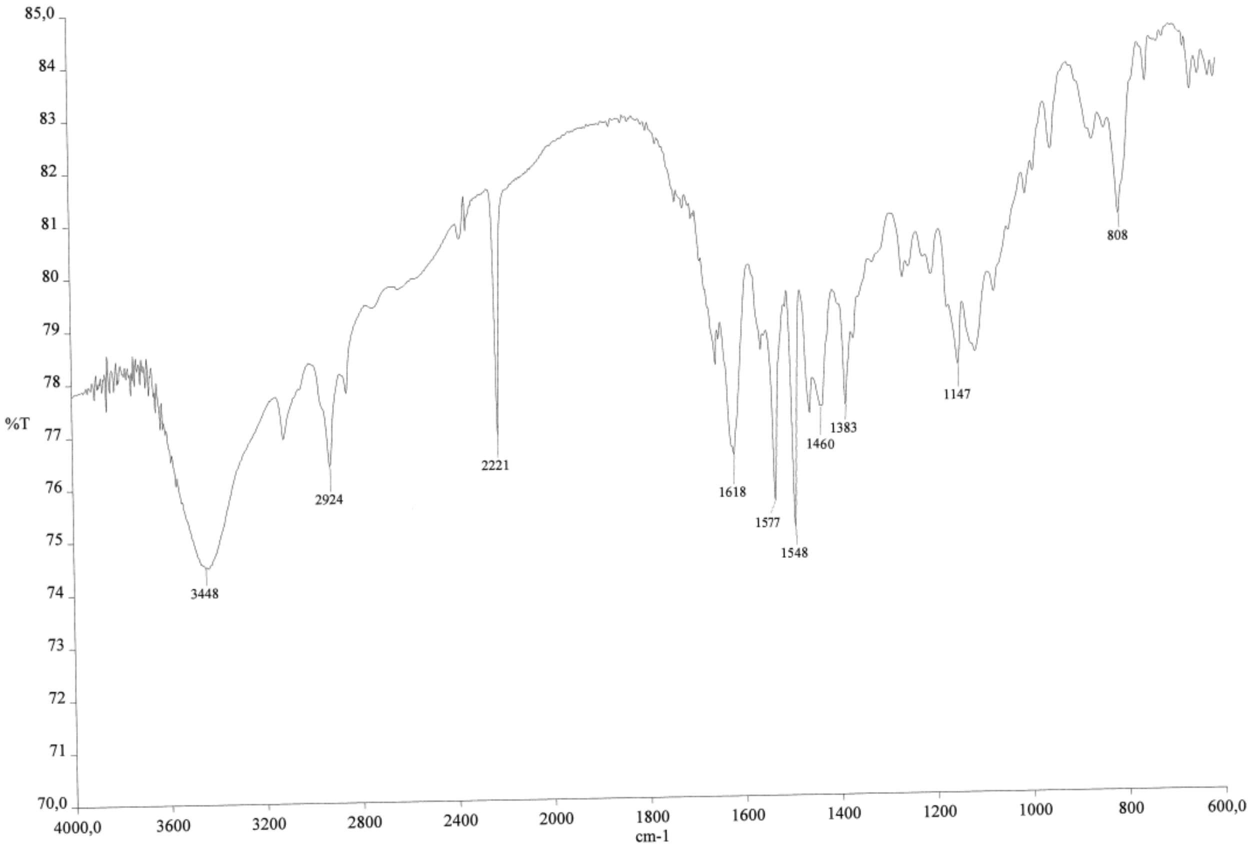

Figure S29. IR spectrum of compound **20.**

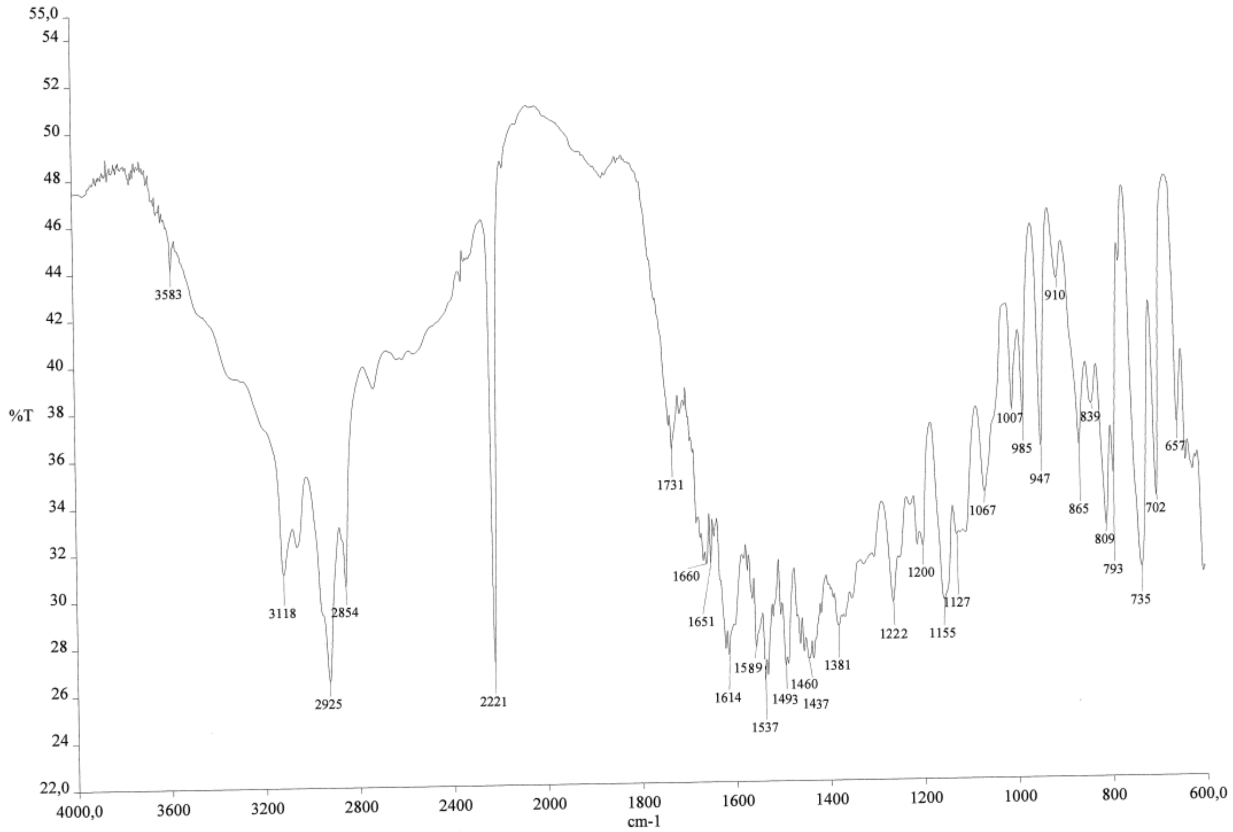

Figure S30. IR spectrum of compound **21.**

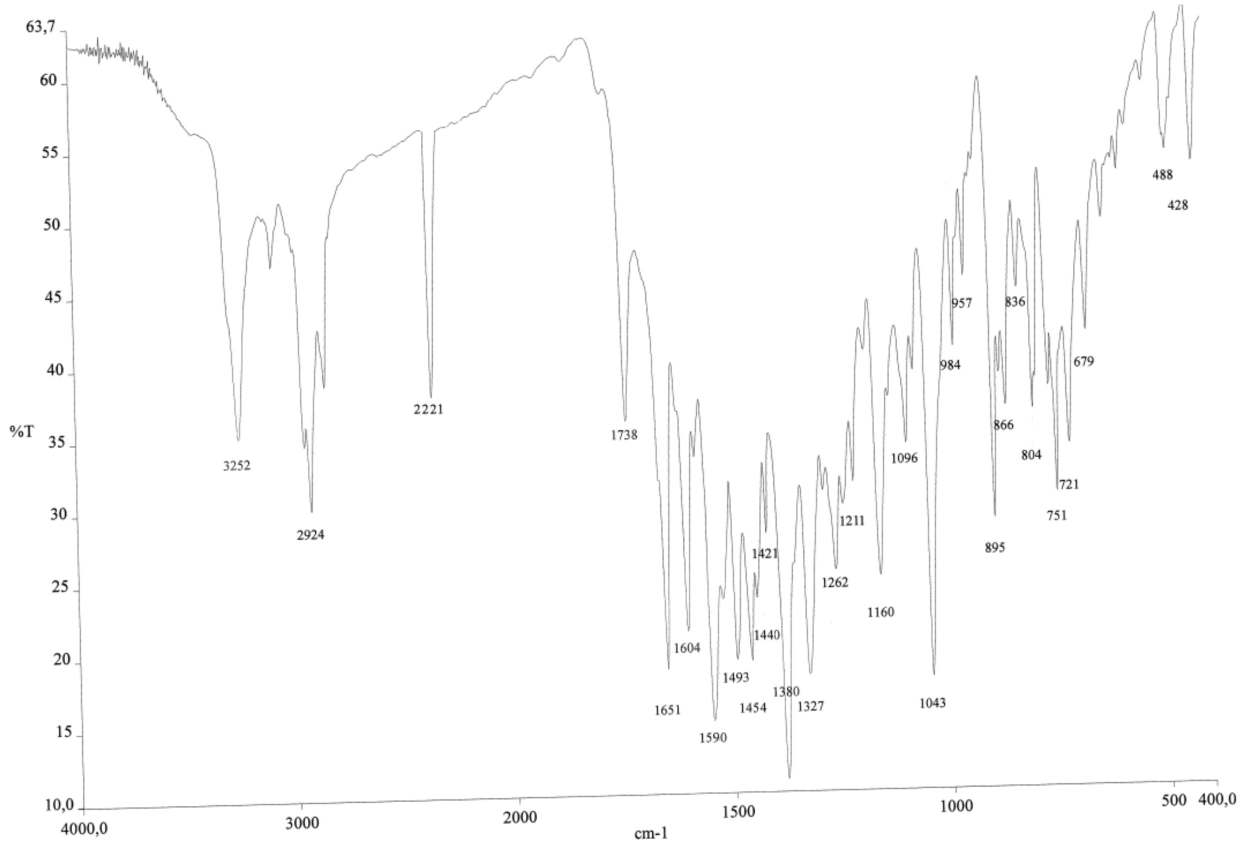

Figure S31. IR spectrum of compound **22.**

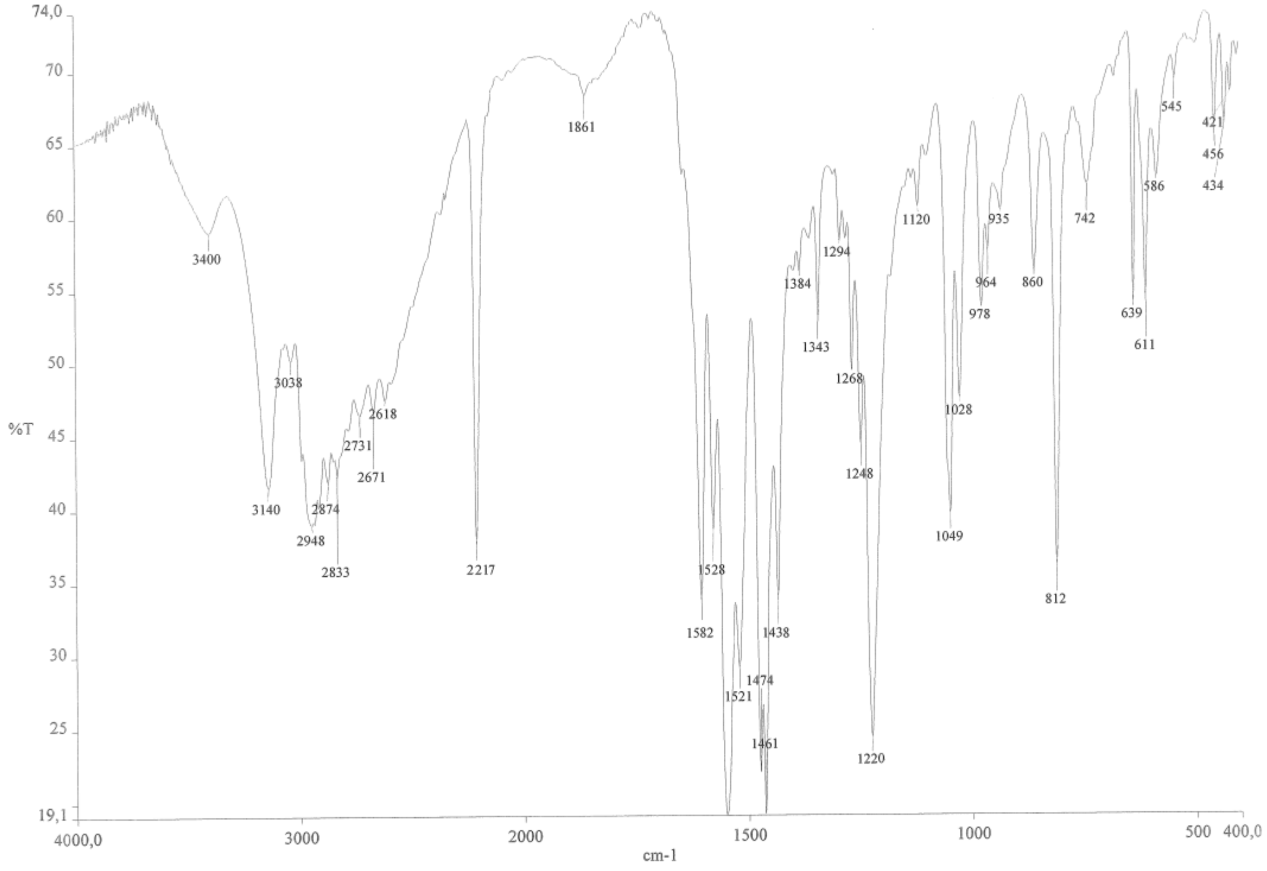

Figure S32. IR spectrum of compound **23.**

Table S1. Antiproliferative activity against human tumor cell lines and the non-tumorigenic cell line HEK-293, expressed as the IC_{50} in nM, determined by the MTT assay. Data are shown as the mean and the standardT deviation of three independent experiments performed in triplicate.

№	HeLa	MCF7	U87 MG	T98G	HepG2	HCT8	HT-29	HT-29*	HEK-293
**8**	45.1 ± 0.3	43.2 ± 0.4	32.4 ± 0.2	41.0 ± 0.3	49.1 ± 0.5	49.4 ± 0.3	52.3 ± 0.4	52.4 ± 0.2	4720 ± 10
**9**	72.5 ± 0.4	69.3 ± 0.5	48.1 ± 0.4	54.4 ± 0.5	70.5 ± 0.5	75.4 ± 0.6	81.1 ± 0.5	80.2 ± 0.6	5200 ± 17
**10**	39.3 ± 0.2	38.4 ± 0.4	22.2 ± 0.1	25.0 ± 0.2	37.1 ± 0.2	40.0 ± 0.3	59.4 ± 0.5	61.4 ± 0.4	4390 ± 12
**11**	233.1 ± 1.4	240.2 ± 2.0	209.3 ± 1.9	219.4 ± 2.2	227.2 ± 2.5	236 ± 1.7	255.2 ± 2.6	257.4 ± 3.0	6820 ± 24
**12**	36.4 ± 0.4	37.3 ± 0.2	17.1 ± 0.1	26.2 ± 0.2	36.4 ± 0.2	40.1 ± 0.3	51.3 ± 0.3	56.5 ± 0.5	3080 ± 31
**13**	57.5 ± 0.3	65.4 ± 0.5	55.4 ± 0.4	54.1 ± 0.3	55.3 ± 0.2	50.1 ± 0.4	64.2 ± 0.4	67.3 ± 0.6	3720 ± 29
**14**	24.2 ± 0.1	26.2 ± 0.1	12.0 ± 0.1	19.0 ± 0.1	31.1 ± 0.3	29.4 ± 0.2	31.1 ± 0.3	33.1 ± 0.4	2270 ± 18
**15**	144.2 ± 1.7	130.1 ± 1.1	115.1 ± 1.4	122.5 ± 1.2	137.2 ± 1.5	141.3 ± 1.0	244.1 ± 1.8	242.1 ± 2.5	6480 ± 37
**16**	207.1 ± 2.0	210.1 ± 1.9	131.3 ± 1.1	127.3 ± 0.9	220.4 ± 2.3	219.1 ± 1.5	211.1 ± 2.0	215.0 ± 2.9	7010 ± 33
**17**	220.2 ± 1.9	215.0 ± 2.7	155.2 ± 1.3	181.4 ± 1.5	229.0 ± 1.7	227.0 ± 2.1	238.0 ± 2.4	230.5 ± 2.5	7250 ± 41
**18**	131.0 ± 1.7	123.4 ± 1.7	93.4 ± 1.0	102.2 ± 1.1	127.0 ± 1.3	115.4 ± 1.3	152.3 ± 1.4	150.4 ± 1.6	6130 ± 35
**19**	307.1 ± 2.8	339.5 ± 2.4	275.4 ± 2.8	294.4 ± 3.0	341.4 ± 3.3	333.2 ± 2.7	355.5 ± 2.3	359.3 ± 4.1	8310 ± 51
**20**	625.4 ± 4.2	631.4 ± 4.9	522.1 ± 3.1	555.4 ± 4.2	634.5 ± 5.6	628.0 ± 3.8	679.4 ± 4.0	682.3 ± 6.3	>10
**21**	722.1 ± 5.0	701.5 ± 5.2	634.0 ± 5.0	689.3 ± 7.5	740.3 ± 6.4	705.4 ± 4.9	749.5 ± 5.1	756.2 ± 5.6	>10
**22**	341.2 ± 2.8	329.0 ± 2.3	312.3 ± 2.6	359.2 ± 3.9	327.3 ± 2.8	335.3 ± 3.3	355.2 ± 3.9	357.1 ± 3.9	>10
**23**	841.1 ± 6.1	963.3 ± 7.1	831.2 ± 4.8	865.2 ± 7.2	877.2 ± 5.9	822.3 ± 6.7	929.1 ± 7.8	927.5 ± 7.4	>10
**ABT-751**	388.5 ± 3.9	180.1 ± 2.8	375.1 ± 5.4	421.0 ± 5.3	327.1 ± 1.1	209.1 ± 2.0	213.5 ± 2.1	250.4 ± 2.9	1.23 ± 0.36
**CA-4**	2.0 ± 0.2	1.4 ± 0.1	10.1 ± 0.1	17.1 ± 0.2	13.4 ± 0.2	220.4 ± 2.0	305.4 ± 2.5	327.5 ± 2.2	721 ± 5
**Colchicine**	9.4 ± 0.1	10.1 ± 0.2	31.2 ± 0.2	37.2 ± 0.1	29.0 ± 0.3	291.0 ± 2.7	375.1 ± 4.0	302.1 ± 3.1	801 ± 7
**TMZ**	>10	>10	135.4	241.5	>10	>10	>10	>10	>10

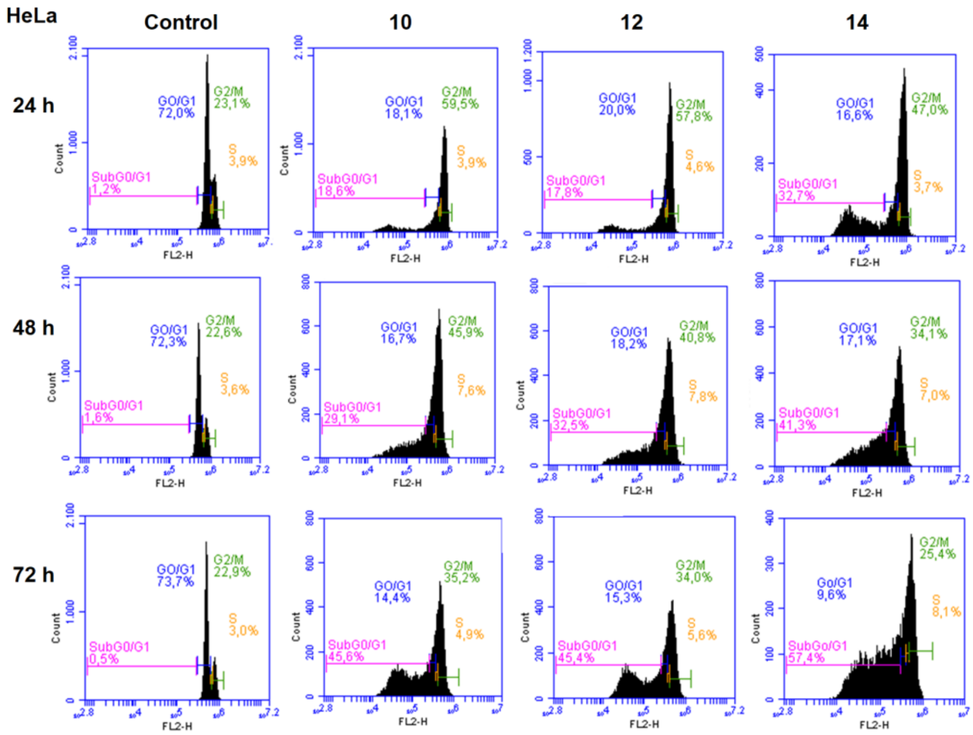

Figure S33. Histograms of the cell cycle distribution of Hela cells after 24, 48, or 72 h of treatment with compounds **10, 12**, or **14** at 100 nM. Untreated control cells were run in parallel. Bars of different colors indicate the positions of the subG0/G1 (pink), G0/G1 (blue), S (orange), and G2/M (green) regions.

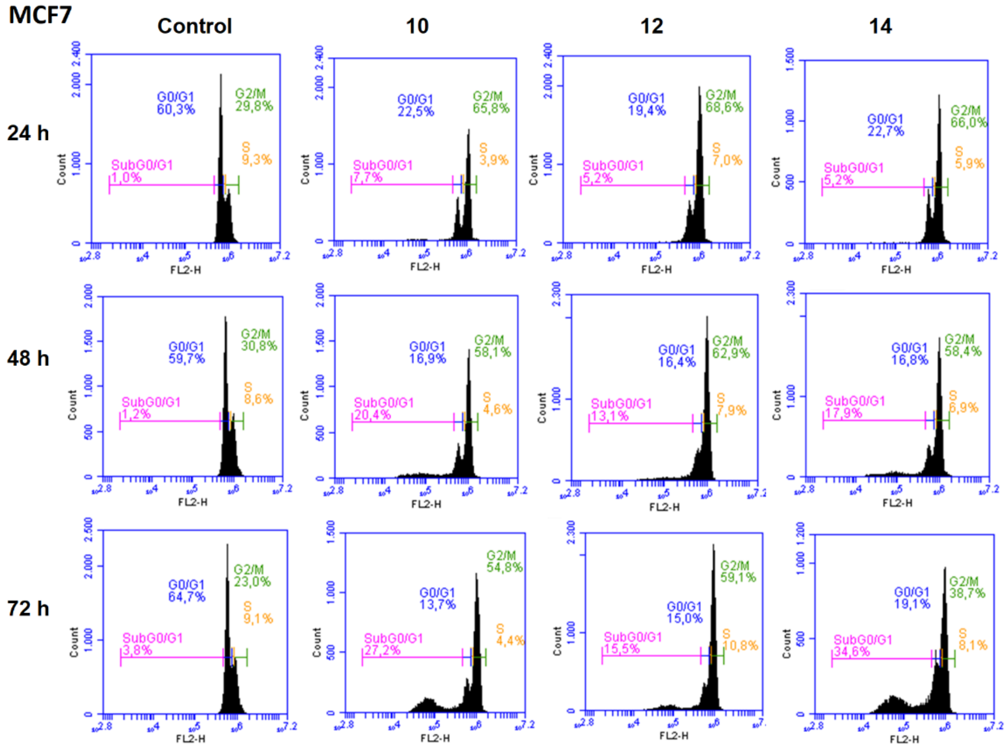

Figure S34. Histograms of the cell cycle distribution of MCF7 cells after 24, 48, or 72 h of treatment with compounds **10, 12**, or **14** at 100 nM. Untreated control cells were run in parallel. Bars of different colors indicate the positions of the subG0/G1 (pink), G0/G1 (blue), S (orange), and G2/M (green) regions.

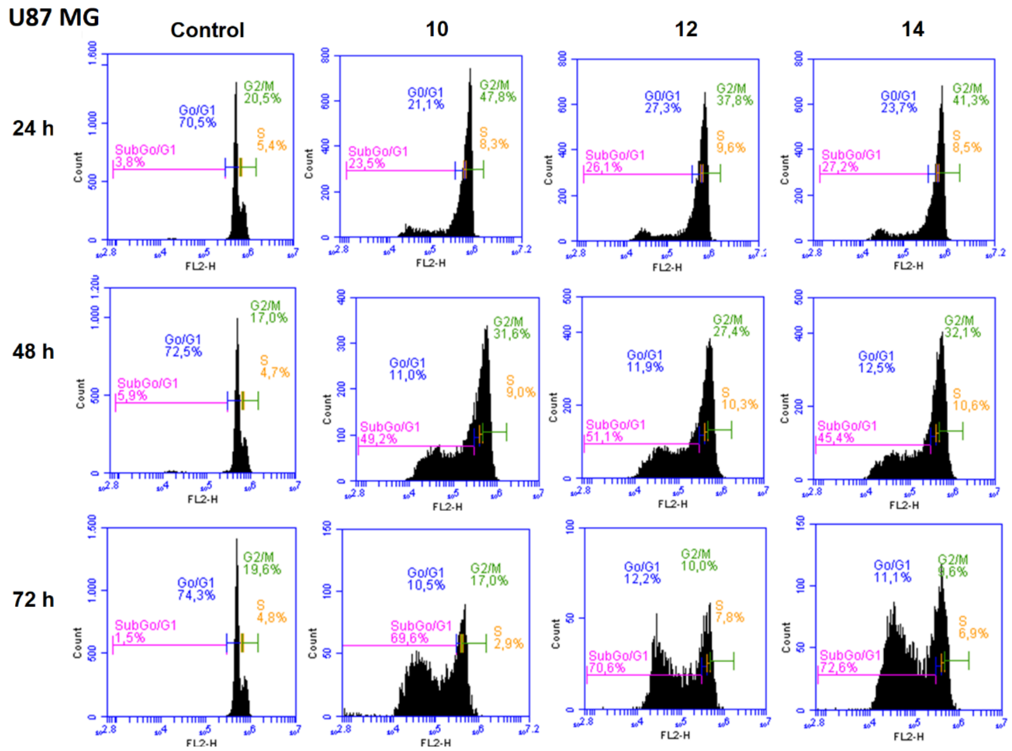

Figure S35. Histograms of the cell cycle distribution of U87 MG cells after 24, 48, or 72 h of treatment with compounds **10, 12**, or **14** at 100 nM. Untreated control cells were run in parallel. Bars of different colors indicate the positions of the subG0/G1 (pink), G0/G1 (blue), S (orange), and G2/M (green) regions.

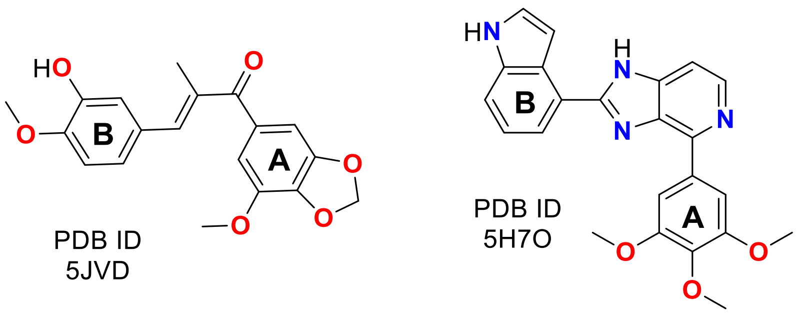

Figure S36. Structures of the ligands of the colchicine site of tubulin that are most frequently selected by the best docking poses: (2*E*)-3-(3-hydroxy-4-methoxyphenyl)-1-(7-methoxy-2H-1,3-benzodioxol-5-yl)-2-methylprop-2-en-1-one (left) and a 2-(1*H*-indol-4-yl)-4-(3,4,5-trimethoxyphenyl)-1*H*-imidazo[4,5-c]pyridine (right). The binding zones (A and B) are indicated.

Pose	Pockets	PDB ID	Norm	Z	E_PL	EPenRot	E/N	E/PM	E_AD	E interm	VdW_Hbond_Des	Eelctr	Eint	Etors	Eunb	Rank	AvE	Cl Size
**6**																		
**6_5JVD_13**	AB	5JVD	0,93	2,21					-9,15	-9,75	-9,77	0,03	-0,61	0,60	-0,61	1	-9,15	100
**6_5H7O_conf_01**	AB	5H7O	0,70	2,00	-87,23	-81,23	-4,15	-0,27										
**7**																		
**7_5JVD_12**	AB	5JVD	0,94	2,29					-8,79	-9,68	-9,70	0,02	-0,64	0,89	-0,64	1,00	-8,77	100
**7_6LSM_conf_07**	AB	6LSM	0,72	2,37	-87,89	-79,89	-4,00	-0,28										
**8**																		
**8_6LSM_conf_01**	AB	6LSM	1,00	2,66	-95,86	-91,86	-4,17	-0,28										
**8_5JVD_34**	AB	5JVD	1,00	1,92					-10,04	-10,64	-10,65	0,01	-0,81	0,60	-0,81	1	-9,93	98
**9**																		
**9_5H7O_conf_01**	AB	5H7O	1,00	2,92	-96,94	-90,94	-4,04	-0,28										
**9_5JVD_23**	AB	5JVD	0,97	1,85					-9,65	-10,55	-10,57	0,02	-0,95	0,89	-0,95	1	-9,63	85
**12**	0.045	0.043	0.032	0.041	0.049	0.049	0.052	4.72										
**12_5JVD_69**	AB	5JVD	1,00	2,80					-10,67	-11,57	-11,46	-0,11	-0,86	0,89	-0,86	1,00	-10,58	91
**12_7CQP_conf_01**	AB	7CQP	0,93	3,00	-97,92	-91,92	-3,92	-0,26										
**13**	0.072	0.069	0.048	0.054	0.070	0.075	0.081	5.20										
**13_5H7O_conf_01**	AB	5H7O	1,00	3,39	-103,38	-95,38	-3,98	-0,28										
**13_5JVD_43**	AB	5JVD	0,97	2,71					-10,31	-11,50	-11,40	-0,10	-0,91	1,19	-0,91	1	-10,25	78
**16E**	0.039	0.038	0.022	0.025	0.037	0.040	0.059	4.39										
**16E_5JVD_77**	AB	5JVD	1,00	2,83					-11,53	-12,72	-12,60	-0,12	-0,82	1,19	-0,82	1	-11,42	89
**16E_5JVD_conf_01**	AB	5JVD	0,90	3,04	-101,13	-93,13	-3,89	-0,26										
**16Z**	0.039	0.038	0.022	0.025	0.037	0.040	0.059	4.39										

**16Z_5H7O_conf_01**	AB	5H7O	1,00	3,69	-98,83	-90,83	-3,80	-0,25										
**16Z_5JVD_83**	AB	5JVD	0,96	3,01					-11,47	-12,66	-12,48	-0,18	-0,86	1,19	-0,86	1,00	-11,33	67
**17E**	0.233	0.240	0.209	0.219	0.227	0.236	0.255	6.82										
**17E_5JVD_37**	AB	5JVD	1,00	2,85					-11,17	-12,66	-12,59	-0,07	-0,89	1,49	-0,89	1	-11,1	92
**17E_5H7O_conf_01**	AB	5H7O	1,00	3,53	-109,55	-99,55	-4,06	-0,29										
**17Z**	0.233	0.240	0.209	0.219	0.227	0.236	0.255	6.82										
**17Z_5H7O_conf_01**	AB	5H7O	1,00	3,46	-98,58	-88,58	-3,65	-0,26										
**17Z_5JVD_26**	AB	5JVD	0,97	2,99					-11,13	-12,62	-12,46	-0,16	-0,90	1,49	-0,90	1	-11,01	59
**20**	0.036	0.037	0.017	0.026	0.036	0.040	0.051	3.08										
**20_5JVD_100**	AB	5JVD	0,99	2,67					-11,08	-11,68	-11,68	0,00	-0,84	0,60	-0,84	1	-11,07	95
**20_7CQP_conf_01**	AB	7CQP	0,92	2,86	-97,12	-91,12	-3,88	-0,26										
**21**	0.057	0.065	0.055	0.054	0.055	0.050	0.064	3.72										
**21_5H7O_conf_01**	AB	5H7O	1,00	3,34	-102,78	-94,78	-3,95	-0,28										
**21_5JVD_94**	AB	5JVD	0,97	2,66					-10,67	-11,57	-11,57	0,01	-0,87	0,89	-0,87	1	-10,62	100
**10**	0.024	0.026	0.012	0.019	0.031	0.029	0.031	2.27										
**10_5H7O_conf_02**	AB	5H7O	0,98	2,82	-98,45	-92,45	-4,10	-0,28										
**10_5JVD_15**	AB	5JVD	0,97	1,94					-10,25	-11,15	-11,17	0,02	-0,97	0,89	-0,97	2	-10,2	61
**11**	0.144	0.130	0.115	0.122	0.137	0.141	0.244	6.48										
**11_5H7O_conf_01**	AB	5H7O	1,00	3,05	-103,40	-95,40	-4,14	-0,28										
**11_5JVD_11**	AB	5JVD	1,00	2,16					-10,55	-11,74	-11,76	0,02	-1,03	1,19	-1,03	1	-10,45	94
**14**	0.207	0.210	0.131	0.127	0.220	0.219	0.211	7.01										
**14_5H7O_conf_01**	AB	5H7O	1,00	3,25	-105,24	-97,24	-4,05	-0,27										
**14_5JVD_79**	AB	5JVD	1,00	2,71					-10,88	-12,08	-11,97	-0,11	-0,94	1,19	-0,94	1	-10,81	82

**15**	0.220	0.215	0.155	0.181	0.229	0.227	0.238	7.25									
**15_5H7O_conf_01**	AB	5H7O	1,00	3,31	-108,48	-98,48	-4,02	-0,27									
**15_5JVD_57**	AB	5JVD	1,00	2,84					-12,66	-12,55	-0,11	-1,07	1,49	-1,07	1	-11,03	78
**18E**	0.131	0.123	0.093	0.102	0.127	0.115	0.152	6.13									
**18E_5JVD_57**	AB	5JVD	1,00	2,76					-13,14	-13,06	-0,08	-0,88	1,49	-0,88	1,00	-11,57	79,00
**18E_5H7O_conf_01**	AB	5H7O	1,00	3,41	-112,31	-102,31	-4,16	-0,28									
**18Z**	0.131	0.123	0.093	0.102	0.127	0.115	0.152	6.13									
**18Z_5H7O_conf_01**	AB	5H7O	1,00	3,41	-101,12	-91,12	-3,75	-0,25									
**18Z_5JVD_88**	AB	5JVD	0,99	2,99					-13,17	-13,02	-0,15	-1,00	1,49	-1,00	1	-11,61	55
**19E**	0.307	0.339	0.275	0.294	0.341	0.333	0.355	8.31									
**19E_5JVD_20**	AB	5JVD	1,00	2,69					-13,83	-13,72	-0,11	-1,05	1,79	-1,05	1	-11,89	86
**19E_5H7O_conf_01**	AB	5H7O	1,00	3,39	-114,63	-102,63	-4,09	-0,28									
**19Z**	0.307	0.339	0.275	0.294	0.341	0.333	0.355	8.31									
**19Z_5H7O_conf_01**	AB	5H7O	1,00	3,44	-105,00	-93,00	-3,75	-0,26									
**19Z_5JVD_67**	AB	5JVD	1,00	2,97					-13,74	-13,59	-0,15	-1,10	1,79	-1,10	1	-11,81	55
**22**	0.625	0.631	0.522	0.555	0.634	0.628	0.679	>10									
**22_5H7O_conf_01**	AB	5H7O	1,00	3,48	-102,41	-96,41	-4,10	-0,28	9,82								
**22_5H7O_6**	AB	5H7O	1,00	2,30					-11,68	-11,70	0,02	-0,83	0,60	-0,83	1	-11,08	98
**23**	0.722	0.701	0.634	0.689	0.740	0.705	0.749	>10									
**23_5H7O_conf_01**	AB	5H7O	1,00	2,90	-103,44	-95,44	-3,98	-0,28									
**23_5JVD_82**	AB	5JVD	1.00	2,27					-11,63	-11,64	0,01	-0,76	0,89	-0,76	1	-10,74	78

Supplementary Table S2. Selected docking data for every compound. Compounds followed by letter represent different isomers. Pose: Ligand pose. Pockets: Occupied subpockets. PDB ID: Selected protein. Norm: Normalized Energy values (from 0 to 1). Z: Z scores for the Energy values. E_PL: Energy of PLANTs. EPenRot: PLANTs Energy with a penalty for rotatable bonds. E/N: PLANTs Energy divided by the number of heavy atoms. E/PM: PLANTs Energy divided by the molecular weight of the compound. E_AD: Autodock Energy. Einterm: Autodock Intermolecular Energy. E VdW Hbond: Autodock Energy for the van der Waals interactions. Eelctr: Autodock Electrostatic Energy. Eint: Autodock Internal Energy. Etors: Autodock Torsional Energy. Eunb: Autodock Energy of the unbound ligand. Rank: Autodock Ranking of the pose. AvE: Autodock Average Energy for the ligand. ClSize: Number of representatives in the cluster (out of 100).
